# Mutating both *relA* and *spoT* of enteropathogenic *Escherichia coli* E2348/69 attenuates its virulence and induces interleukin 6 *in vivo*

**DOI:** 10.3389/fmicb.2023.1121715

**Published:** 2023-03-02

**Authors:** Jun Bong Lee, Se Kye Kim, Dalmuri Han, Jang Won Yoon

**Affiliations:** College of Veterinary Medicine, Institute of Veterinary Science, Kangwon National University, Chuncheon, Gangwon, Republic of Korea

**Keywords:** enteropathogenic *Escherichia coli*, stringent response, guanosine tetraphosphate, virulence, interleukin 6

## Abstract

Here, we report for the first time that disrupting both *relA* and *spoT* genes in enteropathogenic *Escherichia coli* E2348/69 can attenuate its virulence and significantly induce interleukin 6 (IL-6) *in vivo*. Our experimental analyses demonstrated that an E2348/69 Δ*relA*Δ*spoT* double mutant strain derepressed the expression of type IV bundle forming pilus (BFP) and repressed the expression of glutamate decarboxylase (GAD) and locus of enterocyte effacement (LEE). Whole genome-scale transcriptomic analysis revealed that 1,564 EPEC genes were differentially expressed in the Δ*relA*Δ*spoT* double mutant strain (cut-off > two-fold). Such depletion of *relA* and *spoT* attenuated the virulence of E2348/69 in a *Caenorhabditis elegans* infection model. Surprisingly, IL-6 was highly induced in porcine macrophages infected with the Δ*relA*Δ*spoT* double mutant strain compared to those with its wildtype strain. Coinciding with these *in vitro* results, *in vivo* murine peritoneal challenge assays showed high increase of IL-6 and improved bacterial clearance in response to infection by the Δ*relA*Δ*spoT* double mutant strain. Taken together, our data suggest that *relA* and *spoT* play an essential role in regulating biological processes during EPEC pathogenesis and that their depletion can affect host immune responses by inducing IL-6.

## 1. Introduction

Enteropathogenic *Escherichia coli* (EPEC) is a zoonotic bacterial pathogen that can cause gastrointestinal diseases such as diarrhea and vomiting (Nataro and Kaper, 1998). Hallmark of EPEC infection is the formation of attaching and effacing (A/E) lesion on small intestine (Moon et al., 1983). There are two major virulence factors responsible for A/E lesion: type IV bundle forming pilus (BFP) and the locus of enterocyte effacement (LEE). EPEC can produce BFP as an initial adhesin to colonize at the small intestine, recruiting individual cells into aggregates referred to as microcolony (Cleary et al., 2004). After dispersal of microcolonies, EPEC can express LEE that encodes type III secretion system (T3SS), ultimately inducing an A/E lesion (Deng et al., 2004).

In general, virulence factors in pathogenic bacteria are tightly regulated. Their expressions are triggered by a different set of cues, such as adaptation to nutrient-limited environment created in the host system (Fang et al., 2016). Sophisticated regulation of gene transcription in response to environmental stresses enables the pathogen to thrive in the host system. One of the adaptive regulatory activities occurring in bacterial system under nutrient limitation is stringent response. This adaptive response is mediated by an alarmone guanosine tetraphosphate (ppGpp; Potrykus and Cashel, 2008). Nutrient starvation such as amino acid depletion could lead to formation of uncharged tRNAs to ribosome to activate RelA, a ribosome-associated ppGpp synthetase. RelA can convert GTP and ATP into ppGpp (Wendrich et al., 2002). The same can be achieved by a bifunctional synthetase-hydrolase enzyme SpoT (Xiao et al., 1991). Synthesized ppGpp molecules can bind to RNA polymerase, altering the competition between sigma factors and binding to specific promoters (Jishage et al., 2002). Alteration in RNA polymerase binding could have pleiotropic effects on bacterial gene transcription pattern, favoring expression of genes associated with stringent response, such as amino acid biosynthesis and stress-related operons (Magnusson et al., 2005).

In addition to environmental adaptation, stringent response can regulate the expression of bacterial virulence factors, although its regulatory effects vary depending on bacterial species (Erickson et al., 2004; Pizarro-Cerda and Tedin, 2004; Gaynor et al., 2005). Virulence related traits in several pathogenic *E. coli* are also known to be mediated by stringent response (Aberg et al., 2006; Nakanishi et al., 2006). As for EPEC, ppGpp binding to a global gene regulator BipA can influence its binding specificity and affect global cell response during starvation (Fan et al., 2015). Another study has suggested that deletion of *relA* can reduce transcription of *per* and *bfp* operons and impair EPEC adherence *in vitro* (Spira et al., 2014). However, the effect of a double mutation of *relA* and *spoT* on the pathogenesis of EPEC remains unknown. Moreover, very few studies have documented host immune responses against Δ*relA*Δ*spoT* bacterial pathogens. Intranasal challenge with *Burkholderia pseudomallei* K96243 Δ*relA*Δ*spoT* double mutant strain can induce a protective immune response in mice (Muller et al., 2012). Immunization with *Salmonella* Gallinarum Δ*relA*Δ*spoT* double mutant strain can induce significant antibody response and increase splenic expression of pro-inflammatory cytokines IFN-γ and TGF-β4 in chicken (Park et al., 2010). Similar results have been observed in a murine model challenged with *S*. Typhimurium (Na et al., 2006). However, host immune response toward EPEC Δ*relA*Δ*spoT* double mutant strain has not been reported yet. We hypothesized that depletion of *relA* and *spoT* could induce a global transcriptional shift in EPEC, resulting in virulence attenuation and altered host immune responses. Thus, the objective of this study was to examine virulence potentials of EPEC Δ*relA*Δ*spoT* double mutant strain and consequential effects on host immune response during EPEC infection.

## 2. Materials and methods

### 2.1. Bacterial strains, plasmids, and growth media

Bacterial strains and plasmids used in this study are listed in Supplementary Table S1. In general, bacterial strains were grown at 37°C with aeration and shaking at 250 rpm in Luria-Bertani (LB) (Becton Dickinson and Company, Franklin Lakes, NJ, United States) supplemented with appropriate antibiotics (ampicillin (Ap) at 200 μg/mL, kanamycin (Km) at 50 μg/mL, chloramphenicol (Cm) at 30 μg/mL, and tetracycline (Tc) at 5 μg/mL) when necessary. For microarray analyses, bacterial cells were grown in Dulbecco’s modified Eagle’s medium (DMEM) (Thermo Fisher Scientific, Waltham, MA, United States) under gastrointestinal tract (GIT)-mimicking condition (Donnenberg and Kaper, 1992).

Porcine alveolar macrophage cell line 3D4/31 was maintained in RPMI1640 medium supplemented with 10% fetal bovine serum (FBS), 1% penicillin–streptomycin, and non-essential amino acid (Thermo Fisher Scientific, Waltham, MA, United States) on a 100 mm × 20 mm culture dish (Corning, NY, United States) at 37°C under 5% CO_2_ atmosphere.

### 2.2. Functional inactivation of *relA* and *spoT* genes in the chromosome of enteropathogenic *Escherichia coli*

To investigate the role of ppGpp in EPEC pathogenesis, *relA* and *spoT* mutations were introduced into *E. coli* serotype O127: H6 E2348/69. Since a *spoT* deletion mutation is lethal to *E. coli* with a *relA*^+^ background (Xiao et al., 1991), the double mutant EPEC strain was constructed by consecutive allelic exchange (Miller, 1992) with combination of two conjugative plasmids, pCVD442/*relA*::*aphA-3* and pCVD442/*spoT*::*cat* in *E. coli* strain S17-1 λpir. Initially a strain carrying a single *relA* deletion mutation (*∆relA*::*aphA-3*) was constructed. Then *spoT* deletion mutation was introduced afterward (∆*relA*::*aphA-3* ∆*spoT*::*cat*). Briefly, the recombinant DNA construct pCVD442/*relA*::*aphA-3* was transferred to E2348/69 *via* conjugation and transconjugants were selected with LB agar plates containing Ap. Several Ap- and Km-resistant transconjugants were grown overnight at 30°C in the presence of 6% sucrose to eliminate the plasmid backbone by homologous recombination. Resultant exconjugants were tested for Ap-susceptible and Km-resistant phenotypes. They were further confirmed by PCR and DNA nucleotide sequencing. To create a double *relA*- and *spoT*-deleted mutant, another integration plasmid pCVD442/*spoT*::*cat* was transformed into the *relA* mutant by conjugation as described above, followed by selection with antibiotics. The resulting Ap-susceptible and Cm-resistant exoconjugants were further confirmed by PCR and DNA sequencing and designated a Δ*relA*Δ*spoT* double mutant.

For *relA* and *spoT* complementation, both multi- and low-copy plasmids expressing intact *spoT*, or both *relA* and *spoT* of EPEC were constructed. Briefly, intact *relA* and *spoT* genes were PCR amplified using gene-specific primers with indicated restriction sites. Digested PCR fragments were ligated into corresponding restriction sites in plasmid pUC19 or pACYC184 and transformed into *E. coli* DH5α. Resulting plasmids were confirmed by DNA sequencing. Mutation and complementation of *relA*/*spoT* in EPEC strains were confirmed by growing transformants on minimal glucose (MG) agar plates.

### 2.3. Electron microscopic analysis

Wildtype, Δ*relA*Δ*spoT* mutant, *relA-* and *spoT*-complemented strains were grown overnight at 37°C on LB agar plates. Cells were then collected, washed, resuspended in 2.5% glutaraldehyde in phosphate-buffered saline (PBS), and kept on ice. Samples were sent to the Korea Research Institute of Bioscience and Biotechnology (KRIBB, Daejeon, Korea) and observed under transmission electron microscopy (TEM) (JEM-1200EX, JEOL Ltd.). At least two independent sections were prepared and analyzed.

### 2.4. Two-dimensional proteomic analysis and protein identification

Samples for 2D proteomic analysis were prepared as follows. Bacterial cultures were grown in LB at 37°C for 18 h and diluted 1:100 with 30 ml of pre-warmed LB. They were further incubated at 37°C with aeration and shaking at 250 rpm until optical density at 600 nm (OD_600_) of 0.85. Cells were then placed on ice for 20 min and harvested at 4°C. Bacterial pellets were resuspended in a sample buffer consisting of 7 M urea, 2 M thiourea, 4% (w/v) CHAPS, 1% (w/v) DTT, 2% (v/v) pharmalyte (pH 3.5–10, GE Healthcare, Chicago, IL, United States), and 1 mM benzamidine. These samples were sent to Genomine Inc (Pohang, Korea) for 2D electrophoresis, normalization, and protein identification.

### 2.5. Western blotting

Bacterial cultures grown in LB at 37°C for 18 h were diluted 1:100 with 30 ml of pre-warmed LB and further incubated at 37°C with aeration and shaking at 250 rpm for 6 or 19 h. Whole cell lysates were separated on 10% acrylamide gel with sodium dodecyl sulfate-polyacrylamide gel electrophoresis (SDS-PAGE). Separated proteins were transferred onto Immobilon-P polyvinylidene difluoride (PVDF) membranes (MilliporeSigma, Burlington, MA, United States). Membranes were then blocked with 5% (w/v) non-fat dried milk in PBS plus 0.5% (v/v) Tween-20, incubated with antibodies targeting specific proteins, including affinity-purified rabbit α1 bundlin antiserum (a kind gift from M. Donnenberg at School of Medicine, University of Maryland, United States), anti-GadA (MyBioSource, San Diego, CA, United States), and anti-DnaK (Enzo Life Sciences Inc., Farmingdale, NY, United States) at a dilution of 1:20,000 in PBS containing 1% (w/v) non-fat dried milk and 0.05% (v/v) Tween-20, and reacted with horseradish peroxidase-conjugated anti-rabbit or mouse IgG antibody (Bethyl Laboratories, Montgomery, TX, United States) at a dilution of 1:10,000. Blots were developed with an Enhanced Peroxidase Detection kit (AbClon, Seoul, Korea) following the manufacturer’s instructions.

### 2.6. Glutamate-dependent acid resistance assay

Glutamate-dependent acid resistance assay was performed as previously described (Lee et al., 2018). Briefly, bacterial cultures grown in LB with 0.4% glucose (to repress stationary sigma factor) at 37°C for 19 h were diluted 1:100 with 40 ml of pre-warmed E minimal media containing 0.4% glucose (EG) medium and further incubated at 37°C with aeration and shaking (250 rpm) for 2 h. Bacterial survival rates were determined by colony forming units (CFU) before and after acid shock. All data are shown as means ± standard error of the mean (SEM) from three independent experiments. Statistical significance was analyzed by Student’s *t*-test using SPSS 24 software (SPSS Inc., Chicago, IL, United States).

### 2.7. Analysis of T3SS-dependent secreted protein profile in enteropathogenic *Escherichia coli*

Bacterial cultures grown in LB at 37°C for 18 h were diluted 1:100 with 5 ml of pre-warmed LB and further incubated at 37°C with aeration and shaking (250 rpm) until OD_600_ reached 0.85. Bacterial cultures were kept on ice for 1 min and centrifuged at 13,000 rpm for 5 min. The supernatant was filtered through a 0.45 μM syringe filter to remove any bacterial cells or debris. The filtrate was concentrated with a Vivaspin-500 (MWCO 10 kDa; Sartorius, Goettingen, Germany) by centrifugation at 15,000 rpm for 20 min at 4°C. Protein concentration was determined using a BCA protein assay kit (Thermo Fisher Scientific, Waltham, MA, United States). Concentrated samples were subjected to 10% SDS–PAGE and stained with Coomassie Brilliant Blue.

### 2.8. RNA works and quantitative real-time PCR

RNA extraction was performed as follows. For bacterial RNA extraction, overnight LB cultures were diluted 1:100 in pre-warmed LB containing appropriate antibiotics and incubated at 37°C for 6 h with shaking (250 rpm). Bacterial total RNAs were extracted using a HiYield™ Total RNA Mini Kit (Real Biotech Corporation, Taipei, Taiwan). For 3D4/31 RNA extraction, total RNA was extracted from 3D4/31 cells resuspended in Trizol reagent using a RNeasy mini kit (QIAGEN, Hilden, Germany) in accordance with the manufacturer’s instructions. Residual genomic DNA in crude total RNA was eliminated by DNase I (Ambion, Austin, TX, United States) treatment.

For cDNA synthesis, 3 μg of total RNA was reverse transcribed using 25 μg/mL oligo dT or random primers, 0.5 mM deoxynucleotide triphosphate (Promega, Fitchburg, WI, United States), RNase inhibitor, and PrimeScript™ reverse transcriptase (TaKaRa BIO, Kusatsu, Shiga, Japan) in accordance with the manufacturer’s instructions. For qRT-PCR reaction, each well contained 10 μL 2 × FastStart DNA Master SYBR Green I (Roche, Basel, Switzerland), 2 pM primers, 0.5 μL cDNA, and distilled water. Primers used in this study are listed in Supplementary Table S2. A housekeeping gene *TBP1* was used an internal reference gene for 3D4/31 qRT-PCR (Nygard et al., 2007) and the *rpoB* gene was used as internal control for bacterial qRT-PCR (Leverton and Kaper, 2005; Crane et al., 2007). PCR was performed using a LightCycler® 96 system (Roche, Basel, Switzerland). Thermal conditions consisted of a pre-incubation at 95°C for 10 min, followed by 45 cycles with denaturing at 95°C for 10 s, annealing at 60°C for 10 s, and elongation at 72°C for 10 s. To confirm a single PCR product, melting curves were generated after final elongation by increasing temperature from 65°C to 95°C. Gene expression levels were analyzed using the comparative delta–delta Ct (2^−ΔΔCt^) method (Livak and Schmittgen, 2001). All data are shown as means ± SEM from three independent experiments. Statistical significance was analyzed by Student’s *t*-test using SPSS 24 software.

### 2.9. Nematode killing assay

Germ line-defective and temperature-sensitive *Caenorhabditis elegans* strain SS104 was obtained from the Caenorhabditis Genetic Center at the University of Minnesota. *C. elegans* were maintained at 15°C on nematode growth media (NGM) (0.3% NaCl, 0.25% peptone, 5 mg/ml cholesterol, 1 M CaCl_2_, 1 M MgSO_4_, 1 M potassium phosphate buffer, 2.5% agar) plates and propagated on a non-pathogenic feeder strain *E. coli* OP50. In brief, the *C. elegans* strain N2 was propagated on NGM plates at 15°C and synchronized. Synchronized worms were fed on a lawn of the non-pathogenic feeder strain *E. coli* OP50 for 72 h until they reached Larva 4 stage known to be hypersensitive to pathogens. These nematodes were transferred onto NGM plates containing 10 μl of EPEC O127:H6 or *E. coli* OP50 strains pre-cultured in LB media at 37°C overnight with shaking at 150 rpm and then incubated at 25°C. Live worms were scored every 24 h. Worms were considered dead if they did not respond to tapping with a platinum wire pick. Three independent experiments were performed. Data were recorded as the mean survival percentage ± standard deviation. Biological significance between wild-type and mutant *E. coli* strains in killing *C. elegans* was statistically determined by repeated measures Analysis of Variance using SPSS 24 software.

### 2.10. Whole genome-scale transcriptomic analysis

For microarray analyses, bacterial cultures grown in LB at 37°C for 18 h were diluted 1:100 with 30 ml of pre-warmed DMEM and further incubated at 37°C with shaking (250 rpm) until OD_600_ reached 0.85. Cultures were immediately mixed with five volumes of RNAlater (Thermo Fisher Scientific, Waltham, MA, United States) to stabilize RNA and stored at 4°C until further use. Total RNA was extracted using RiboPure™-Bacteria Kit (Thermo Fisher Scientific, Waltham, MA, United States) according to the manufacturer’s instructions. The quality of each RNA sample was confirmed by electrophoresis. Two independent sets of RNA samples were sent to Macrogen (Daejeon, Korea) for microarray analysis using a 35-mer Genomic Microarray for *E. coli* O127:H6 strain E2348/69 (CombiMatrix Prokaryotic 12 K Platform; CombiMatrix, San Francisco, CA, United States).

For macrophage transcriptome analyses, 1 × 10^6^ CFU of pre-cultured bacteria were inoculated into a pre-warmed LB broth and incubated at 37°C with vigorous shaking for 19 h. Cultured bacterial cells were pelleted, washed, and resuspended in RPMI1640 medium (Thermo Fisher Scientific, Waltham, MA, United States). 3D4/31 cells were cultured in a 100 mm × 20 mm dishes until reaching 80% confluency. They were then subcultured in 6-well cell culture plates (SPL Life Sciences, Pocheon, Korea) at density of 1 × 10^6^ cells per well and incubated at 37°C under 5% CO_2_ atmosphere for 16 h. EPEC strains were then inoculated into each well with multiplicity of infection (MOI) of 50. Cells were then incubated at 37°C under 5% CO_2_ atmosphere for 3 h. Culture medium containing uninfected bacterial cells in each well was aspirated. Attached macrophage cells were washed, treated with 1 ml TRIzol® (Ambion, Austin, TX, United States), and stored at −80°C until further use. Total RNA from 3D4/31 was isolated using a RNeasy mini kit (QIAGEN, Hilden, Germany) in accordance with the manufacturer’s instructions. Potential genomic DNA contaminations in total RNA samples were eliminated by treatment with DNase I (Ambion, Austin, TX, United States). RNA-sequencing (RNA-seq) library construction, sequencing, normalization, and functional analysis were conducted by Macrogen Inc (Daejeon, Korea).

### 2.11. Animal challenge and bacterial clearance

All experimental and animal care procedures were approved by Kangwon National University Institutional Animal Care and Use Committees (KW-180227-1) and performed in compliance with the standard guidelines. Female C57BL/6 N mice at 8 weeks old were purchased from Koatech Inc (Pyeongtaek, Korea), acclimated for 5 days, and challenged intraperitoneally with 1.0 × 10^8^ CFU of one of the three EPEC strains: wildtype, Δ*relA*Δ*spoT* mutant, or *relA-* and *spoT*-complemented mutant strain. At 24 and 48 h post infection, blood samples were collected from retro-orbital plexus. After euthanization, peritoneal lavage was performed, and fluid samples were collected with 4 ml of PBS.

To calculate the number of EPEC survived in peritoneal cavity, samples were serially diluted, plated onto LB agar supplemented with Tc, and incubated at 37°C for 19 h. The number of viable colonies was counted and statistical significance between groups was obtained with Student’s *t*-test using SPSS 24 software.

### 2.12. Cytokine quantification by enzyme-linked immunosorbent assay

Cytokine samples from 3D4/31 cells were prepared as follows. Macrophages were infected with EPEC strains as aforementioned. Culture supernatants were transferred to 1.5 mL tubes, centrifuged to remove bacterial pellets and debris, and stored at −80°C until further use. Levels of porcine interleukin 6 (IL-6) and interleukin 8 (IL-8) in cell culture supernatants were quantified with pig IL-6 and IL-8 Quantikine ELISA kit (R&D systems, Minneapolis, MN, United States) using instructions provided by the manufacturer.

Cytokine samples from murine challenge experiments were prepared as follows. Blood samples collected from retro-orbital plexus were centrifuged at 6,000 rpm for 15 min. The upper layer containing the serum was transferred to a 1.5-ml tube and stored at −80°C until further use. Concentrations of murine IL-6 and monocyte chemoattractant protein-1 (MCP-1) in the serum and peritoneal lavage fluid were determined with mouse IL-6 and MCP-1 ELISA kits (Invitrogen, Carlsbad, CA, United States) following instructions provided by the manufacturer.

### 2.13. Statistics analysis

At least three independent experiments were performed in this study. All quantitative data are presented as mean ± SEM. Statistical analysis was performed *via* Student’s t-test using SPSS 24 software.

## 3. Results

### 3.1. Construction of a Δ*relA*Δ*spoT* double mutant enteropathogenic *Escherichia coli* strain

**Figure 1:**
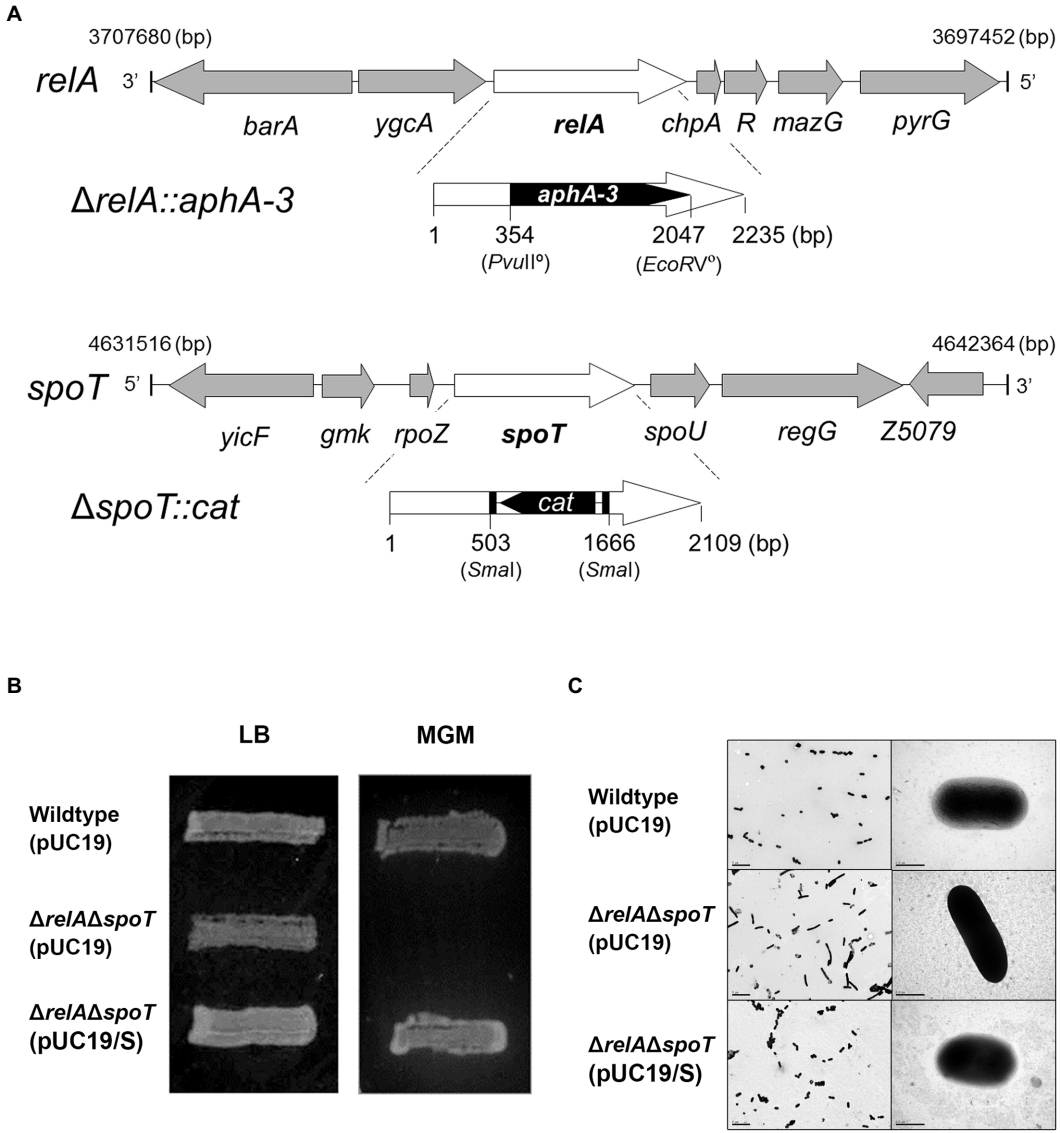
Defined mutation of both *relA* and *spoT* genes encoding a ppGpp synthetase in EPEC. **(A)** Mutation of the two ppGpp synthetase genes, *relA* and *spoT*, in EPEC O127:H6 chromosome. The mutated *ΔrelA::aphA-3* and *ΔspoT::cat* were introduced into EPEC O127:H6 strain E2348/69. **(B)** The ppGpp-dependent growth on a minimal glucose (MG) medium. Cells were streaked on an MG agar plate and grown at 37°C for 19 h. Wildtype (pUC19), E2348/69 strain carrying pUC19; Δ*relA*Δ*spoT* (pUC19), E2348/69 Δ*relA*Δ*spoT* mutant carrying pUC19; Δ*relA*Δ*spoT* (pUC19/S), E2348/69 Δ*relA*Δ*spoT* mutant carrying pUC19/S. **(C)** The morphological comparison between EPEC strains by using transmissible electron microscopy.

In this study, both *relA* and *spoT* genes on the chromosome of EPEC E2348/69 strain were mutated by a sequential conjugation. Because a single *spoT* deletion is known to be lethal, a single Δ*relA* mutation was initially introduced, followed by Δ*spoT* mutation to create a Δ*relA*Δ*spoT* double mutant strain (Figure 1A). Functional inactivation was confirmed by assessing growth defect on a MG agar medium as a previous report showed that *relA* and *spoT* were essential for EPEC growth on MG medium (Jin et al., 2012). As expected, Δ*relA*Δ*spoT* mutant strain was unable to grow on MG medium at 37°C (Figure 1B). Complementation of *spoT* with plasmid pUC/spoT restored its growth, confirming functional inactivation of *relA* and *spoT*. Since the Δ*relA*Δ*spoT* double mutant could not acquire a coccoid form (Magnusson et al., 2007), we further validated Δ*relA*Δ*spoT* mutation by observing morphological changes using TEM. As a result, Δ*relA*Δ*spoT* mutant strain showed a more elongated morphology than its wildtype strain and a *spoT*-complemented mutant strain (Figure 1C). Collectively, a Δ*relA*Δ*spoT* double mutant was constructed and verified by matching previously reported phenotypes.

### 3.2. Expressions of key virulence factors are altered in Δ*relA*Δ*spoT* enteropathogenic *Escherichia coli*

**Figure 2:**
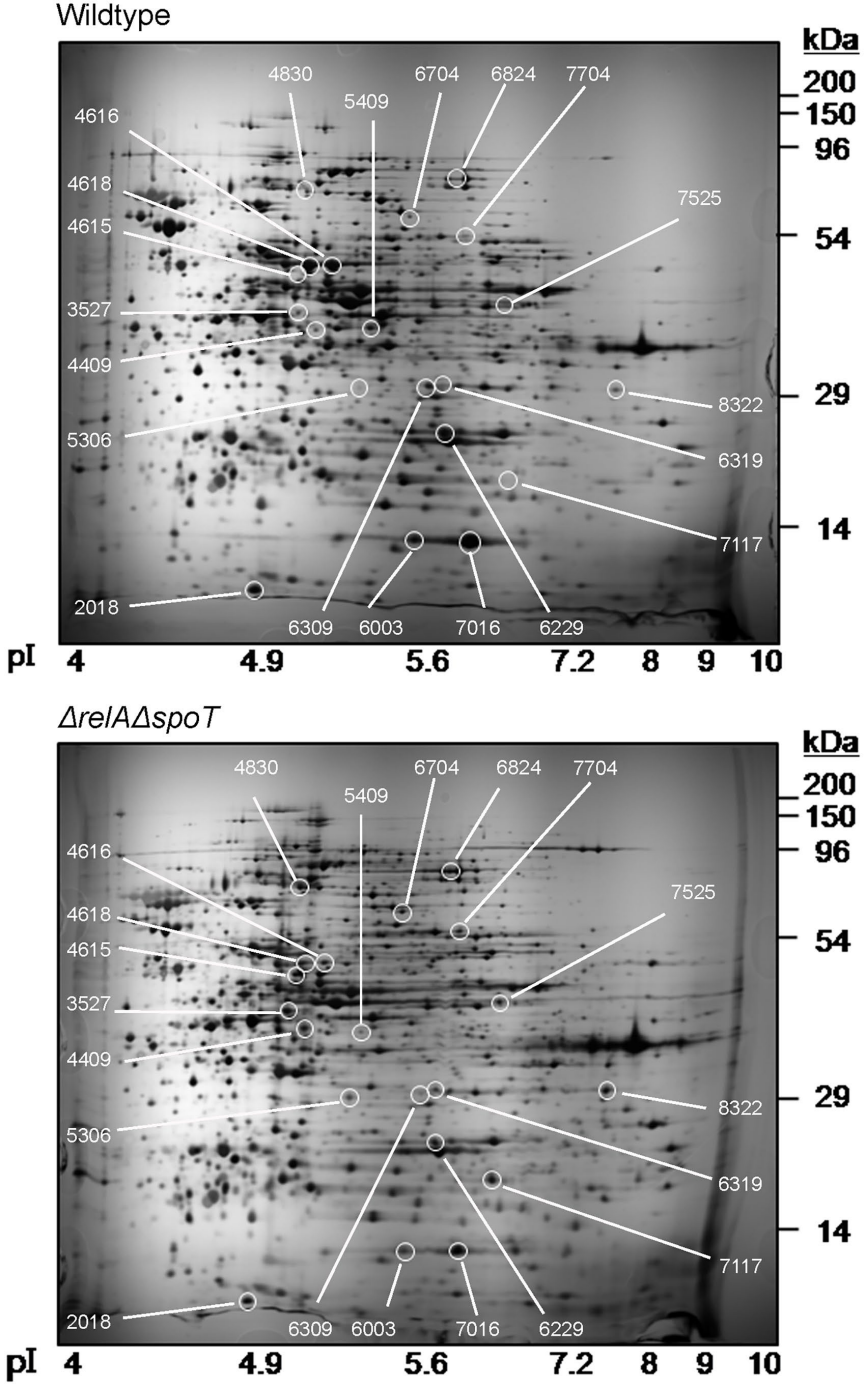
Two-dimensional proteomic analysis of wildtype and Δ*relA*Δ*spoT* mutant strains. Wildtype and mutant strains grown in Luria-Bertani (LB) at 37°C for 18 h were diluted in pre-warmed LB and further incubated at 37°C. Equal amounts (200 μg) of proteins were obtained from each strain and subjected to two-dimensional proteomic analysis. Top, wildtype, E2348/69 strain; bottom, Δ*relA*Δ*spoT*, E2348/69 Δ*relA*Δ*spoT* mutant.

We then performed a 2D proteomic analysis using whole bacterial proteins to identify EPEC proteins differentially expressed in the Δ*relA*Δ*spoT* double mutant grown in a rich LB medium. Our experimental analyses from two independent trials revealed that 153 out of 1,600 protein spots were consistently up- or down-regulated in the Δ*relA*Δ*spoT* double mutant than in its parental strain (Figure 2). Top 20 protein spots with significant differences in their fold changes were then subjected to matrix-assisted laser desorption/ionization time-of-flight (MALDI-TOF) analysis for identification (Tables 1, 2). Notably, the expression level of BfpF, the eighth gene in the *bfp* cluster which encoded BFP, was significantly increased (>726-fold) in the Δ*relA*Δ*spoT* double mutant. Repression of three proteins, a universal stress protein UspA and *gad* operon proteins GadA and GadB, isoforms of glutamate decarboxylase (Gad), was also observed (0.6, 0.2, and 0.1, respectively). Overall, our 2D proteomic analysis showed that *relA* and *spoT* depletion affected expression patterns of various proteins including virulence factors in EPEC (Supplementary Figure S1).

**Table 1:** Identification of EPEC proteins upregulated in the Δ*relA*Δ*spoT* EPEC (>1.5-fold).

No.	SSP	MR	PI	Fold difference (Δ*relA*Δ*spoT*/WT)		PMF spot identity	Gene ID	Reference^a^
				First	Second			
1	7,117	18.27	6.54	2184.2	2167.1	Enhanced serine sensitivity protein, SseB		|ref.|NP_289079.1
2	3,527	39.46	4.98	2132.5	1112.7	Elongation factor Tu (EF-Tu-GDP), TufA	E2348C_4287	|pdb|1EFM
3	6,319	28.83	5.81	726.3	966.8	Bundle forming pili, BfpF	E2348_P1_010	|emb|CAA92333.1
4	4,615	45.06	5.02	609.4	862.5	Porphobilinogen Synthase, HemB	E2348C_0309	|pdb|1I8J
5	5,306	28.43	5.28	8.1	13.4	Homoserine kinase, ThrB	E2348C_0003	|gb|AAN06428.1
6	4,409	37.03	5.07	7.9	4.3	Porphobilinogen synthase, HemC	E2348C_4103	|ref.|YP_002331562.1
7	6,824	73.98	5.97	4.5	2.3	Formate acetyltransferase 1, TdcE		|ref.|NP_286778.1
8	7,525	40.19	6.64	3.4	1.7	Carbamoyl phosphate synthetase large subunit, CarB	E2348C_0033	|pdb|1 T36
9	6,704	58.41	5.52	2.9	2.0	Aspartyl-tRNA synthetase, AspS		|ref.|NP_754172.1
10	7,704	52.54	6.08	2.6	6.0	*Escherichia coli* hypothetical ATP binding protein	E2348C_1234	|emb|CAA78294.1
11	8,322	28.15	8.05	2.5	2.4	3-deoxy-D-manno-octulosonic acid 8-phosphate synthetase, KdsA	E2348C_1338	|emb|CAA29067.1
12	4,830	68.40	5.04	2.2	20.0	EFACTOR_GTP; similar to elongation factor G, BipA	E2348C_4177	|gb|AAB03005.1

^a^Reference ID numbers were obtained from BLAST homology search.

**Table 2:** Identification of EPEC proteins downregulated in the Δ*relA*Δ*spoT* EPEC (>1.5-fold).

No.	SSP	MR	PI	Fold difference (Δ*relA*Δ*spoT*/WT)		PMF spot identity	Gene ID	Reference^a^
				First	Second			
1	2018	9.80	4.80	0.6	0.4	Universal stress protein, UspA	E2348C_3732	|ref.|NP_290066.1
2	6,003	12.70	5.54	0.5	0.4	DNA starvation/stationary phase protection protein, Dps	E2348C_0764	|ref.|NP_286576.1
3	6,229	22.35	5.81	0.4	0.4	Uridine phosphorylase, Udp		|ref.|NP_290463.1
4	7,016	12.60	6.14	0.4	0.2	DNA binding protein with protective role during starvation, PexB	E2348C_0764	|gb|AAA21855.1
5	5,409	36.30	5.33	0.2	0.4	Amino-methyltransferase 1, GcvT	E2348C_3157	|ref.|NP_417381.1
6	6,309	28.77	5.61	0.2	0.2	*Escherichia coli* Heat Shock Protein, HchA	E2348C_2078	|pdb|1ONS
7	4,618	46.83	5.05	0.2	0.1	Glutamate decarboxylase alpha, GadA	E2348C_3759	|ref.|NP_756190.1
8	4,616	46.71	5.16	0.1	0.3	Glutamate decarboxylase beta, GadB	E2348C_1620	|sp.|Q8FHG5

^a^Reference ID numbers were obtained from BLAST homology search.

**Figure 3:**
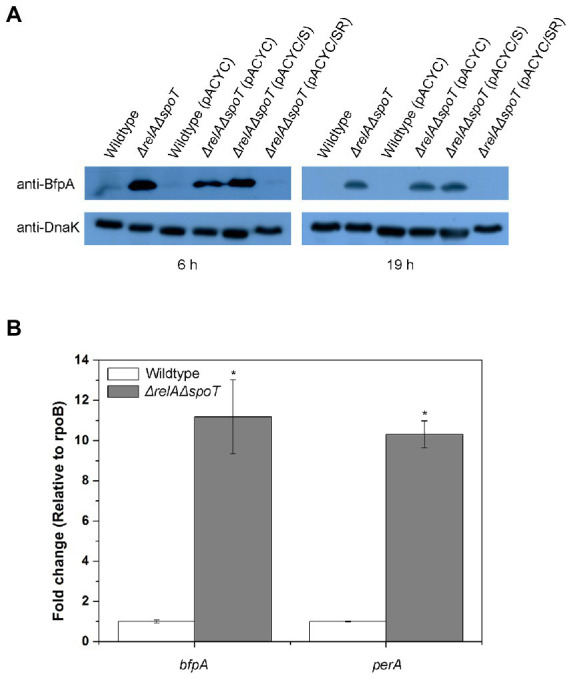
Derepression of type IV bundlin in the Δ*relA*Δ*spoT* EPEC. Wildtype and mutant strains grown in Luria-Bertani (LB) at 37°C for 18 h were diluted with pre-warmed LB and further incubated at 37°C for 6 or 19 h. **(A)** Equal amounts (200 μg) of proteins were obtained from each strain and analyzed by western blotting to detect bundlin. DnaK was used as an internal control. Wildtype, E2348/69 strain; Δ*relA*Δ*spoT*, E2348/69 Δ*relA*Δ*spoT* mutant; wildtype (pACYC), E2348/69 carrying pACYC184; Δ*relA*Δ*spoT* (pACYC), E2348/69 Δ*relA*Δ*spoT* carrying pACYC184; Δ*relA*Δ*spoT* (pACYC/S), E2348/69 Δ*relA*Δ*spoT* carrying pACYC/S; Δ*relA*Δ*spoT* (pACYC/SR), E2348/69 Δ*relA*Δ*spoT* carrying pACYC/SR. **(B)** Expression patterns of *bfpA* and *perA* were analyzed by quantitative real-time PCR. Values are presented as mean ± standard error of the mean (*n* = 3). Statistical significance was obtained by Student’s *t*-test (*p < 0.05).

As we identified that one of the *bfp* operon genes was induced in the Δ*relA*Δ*spoT* mutant, we speculated that other genes in the *bfp* operon might also be induced. To confirm this, we examined protein level of BfpA in the Δ*relA*Δ*spoT* mutant by western blotting. When grown in LB, the level of BfpA in Δ*relA*Δ*spoT* mutant was significantly increased, whereas it was not detectable in the parent strain (Figure 3A). Interestingly, the BfpA level in the *spoT*-complemented mutant strain was similar to that in the Δ*relA*Δ*spoT* mutant. However, it was not detectable in *relA-* and *spoT*-complemented mutant strain, coinciding with that in the wildtype strain. This observation suggests that ppGpp can negatively regulate BFP expression in EPEC and that partial restoration of ppGpp synthesis is insufficient to regulate BFP expression. We also assessed transcriptional levels of *bfp* operon genes by qRT-PCR. Notably, *bfpA* and *perA* genes were significantly up-regulated (approximately 10-fold) in the Δ*relA*Δ*spoT* mutant than in the wildtype (Figure 3B). This suggests that intracellular ppGpp can negatively affect the expression of both PerA (an activator of the *bfp* operon) and BfpA.

**Figure 4:**
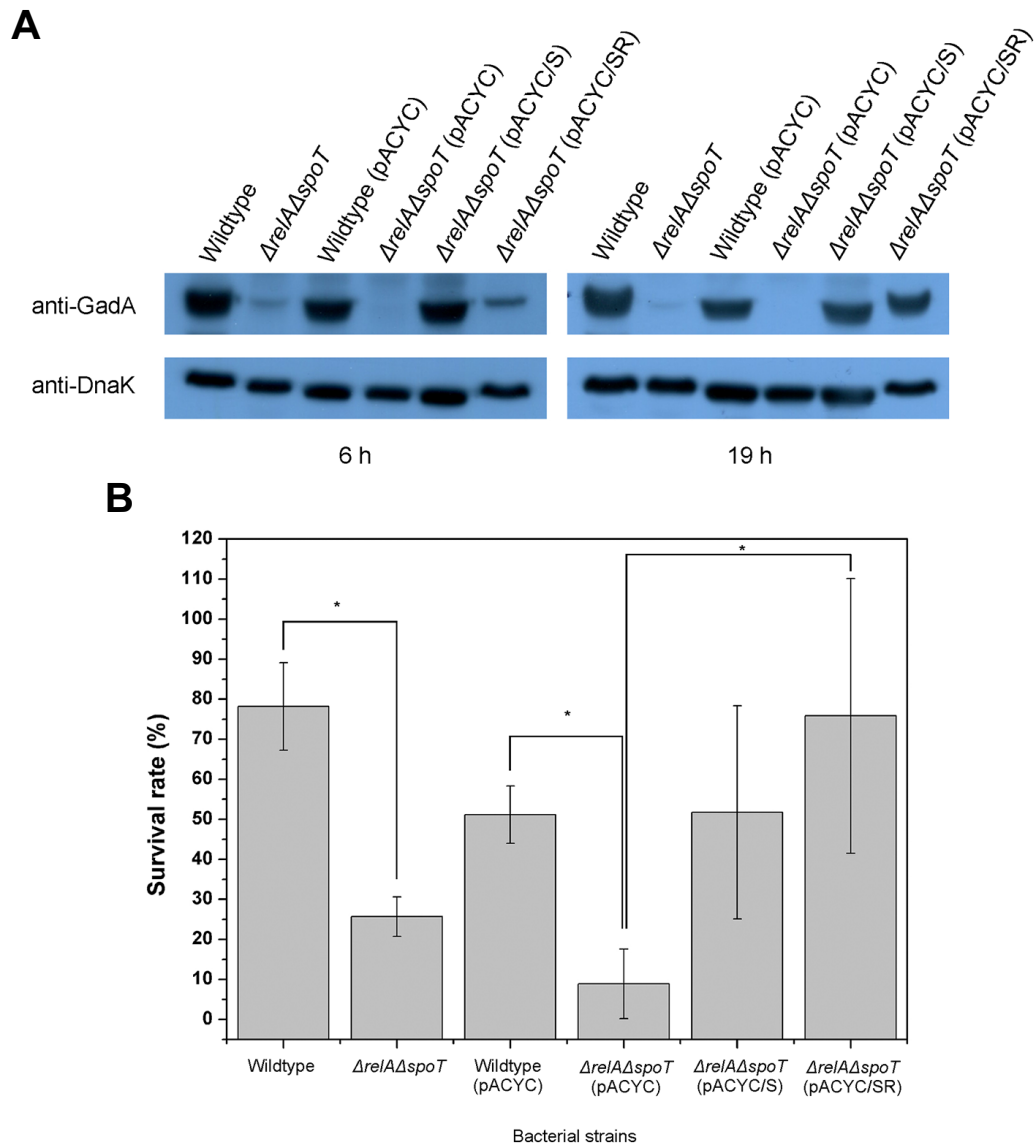
Reduced glutamate decarboxylase (GAD) expression and acid resistance in Δ*relA*Δ*spoT* EPEC. **(A)** Expression of GAD protein. Wildtype and mutant strains grown in Luria-Bertani (LB) at 37°C for 18 h were diluted with pre-warmed LB and further incubated at 37°C for 6 or 19 h. Equal amounts (200 μg) of proteins were obtained from each strain and analyzed by western blotting to detect GadA. DnaK was used as an internal control. Wildtype, E2348/69 strain; Δ*relA*Δ*spoT*, E2348/69 Δ*relA*Δ*spoT* mutant; wildtype (pACYC), E2348/69 carrying pACYC184; Δ*relA*Δ*spoT* (pACYC), E2348/69 Δ*relA*Δ*spoT* carrying pACYC184; Δ*relA*Δ*spoT* (pACYC/S), E2348/69 Δ*relA*Δ*spoT* carrying pACYC/S; Δ*relA*Δ*spoT* (pACYC/SR), E2348/69 Δ*relA*Δ*spoT* carrying pACYC/SR. **(B)** Survival rate of wildtype and mutant strains in E minimal glucose medium (pH 3.0; 1.5 mM glutamate) after incubating at 37°C with shaking for 2 h. Bacterial survival rate was determined by comparing colony forming units before and after acid shock. Values are presented as mean ± standard error of the mean (*n* = 3). Statistical significance was obtained by Student’s *t*-test (*p < 0.05).

As seen in our 2D proteomic analysis, Gad proteins in EPEC were significantly downregulated by *relA* and *spoT* depletion. This finding was also confirmed by western blotting using anti-GadAB antibody (Figure 4A). GadAB proteins play a vital role in GDAR. They also contribute to the extremely low infectious dose of pathogenic *E. coli*, including EHEC O157:H7 (Lin et al., 1996). Decreased expression of GadAB could result in disruption of GDAR responses and increased susceptibility of the bacterium to acid insults. To confirm this, we performed acid resistance assay using EG medium (pH 3.0; 1.5 mM glutamate). As a result, Δ*relA*Δ*spoT* mutant was more susceptible to acid stress than the wildtype or the complemented mutant strains (Figure 4B). These results imply that ppGpp-mediated signaling is required for proper Gad expression to enhance bacterial survival in an acidic environment *via* GDAR.

**Figure 5:**
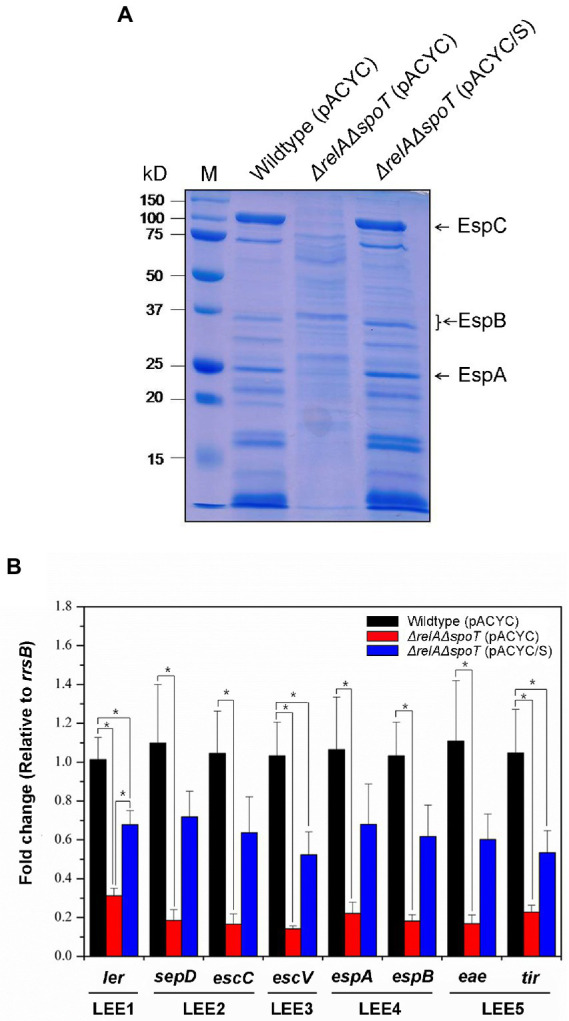
The *relA* and *spoT* dependent expression of LEE pathogenicity island (LPI) genes of EPEC proteins. Wildtype, Δ*relA*Δ*spoT* mutant, and *spoT*-complemented mutant strains grown in Luria-Bertani (LB) at 37°C for 18 h were diluted with pre-warmed LB and further incubated at 37°C. **(A)** Secreted protein profiles of the wild type and its Δ*relA*Δ*spoT* mutant strain. Secreted proteins were harvested, concentrated, and analyzed using 10% sodium dodecyl sulfate–polyacrylamide gel electrophoresis. Lane M, Protein ladder. Wildtype (pACYC), E2348/69 strain carrying pACYC184; Δ*relA*Δ*spoT* (pACYC), E2348/69 Δ*relA*Δ*spoT* mutant carrying pACYC184; Δ*relA*Δ*spoT* (pACYC/S), E2348/69 Δ*relA*Δ*spoT* mutant carrying pACYC/S. **(B)** Transcriptional repression of LPI genes by ppGpp. Expression patterns of major virulence associated genes including *ler*, *sepD*, *escC*, *escV*, *espA*, *espB*, *eae*, and *tir* were analyzed using quantitative real-time PCR. Values are presented as mean ± standard error of the mean (*n* = 3). Statistical significance was obtained by Student’s *t*-test (*p < 0.05).

As aforementioned, ppGpp can regulate various virulence factors in pathogenic bacteria. Since some virulence factors are readily secreted, we surmised that ppGpp might affect the overall EPEC protein secretion. To confirm this, we compared secreteomic profiles of wildtype, Δ*relA*Δ*spoT* mutant, and *spoT*-complemented mutant strain using SDS-PAGE. As shown in Figure 5A, general secretion abilities of the Δ*relA*Δ*spoT* mutant were significantly reduced compared to those of other strains. Interestingly, sizes of several hypo-secreted proteins in the Δ*relA*Δ*spoT* mutant corresponded to those of T3SS components such as EspA, EspB, and EspC (25, 35 and 110 kDa, respectively). T3SS encoded in the LEE pathogenicity island (LPI) plays a central role in the virulence of EPEC (Sekiya et al., 2001). We then performed qRT-PCR to further examine transcriptional patterns of LPI virulence factors, including *ler* (encoded in LEE1), *sepD* (LEE2), *escC* (LEE2), *escV* (LEE3), *espA* (LEE4), *espB* (LEE4), *eae* (LEE5), and *tir* (LEE5). Transcriptional levels of all T3SS genes were significantly decreased in the Δ*relA*Δ*spoT* mutant in comparison with those in its parental strain (Figure 5B), coinciding with secretome results. These results indicate that ppGpp can up-regulate the expression of LPI genes.

Our work demonstrated that loss of ppGpp-mediated signaling altered protein expression not normally observed when growing in a rich medium. Previous EPEC studies used DMEM to mimic a host environment. To address the effect of Δ*relA*Δ*spoT* mutation on transcriptional activities in EPEC under a host-like condition, we performed two independent microarray analyses using wildtype and Δ*relA*Δ*spoT* mutant grown in DMEM. A total of 1,564 genes were differentially transcribed in the Δ*relA*Δ*spoT* mutant compared to the wildtype, including 824 upregulated genes and 740 downregulated genes (Supplementary Table S3). Consistent with results of our experiments, *bfp* genes (such as *bfpA*, *bfpC*, *bfpF*, and *bfpI*) and their regulator *perABC* were activated in the Δ*relA*Δ*spoT* mutant, whereas genes encoding T3SS structural proteins (*escC*, *escD*, *escR*, *escS*, and *escV*) and GDAR genes (*gadA* and *gadBC*) were repressed (Table 3). Collectively, our findings address the importance of ppGpp-mediated signaling in the regulation of major virulence genes (e.g., LEE, BFP, and GAD) in EPEC.

**Table 3:** Virulence associated genes regulated by ppGpp in EPEC.

Ontology	Gene ID	Fold (Δ*relA*Δ*spoT*/WT)	*P*-value (Δ*relA*Δ*spoT*/WT)
Bundle forming pili	*bfpA*; major pilin structural unit bundlin	4.564	0.001
	*bfpC*; hypothetical protein	2.083	0.010
	*bfpF*; nucleotide binding protein	2.374	0.002
	*bfpI*; prepilin	2.703	0.000
Regulator of *bfp* and *ler*	*perA*; transcriptional activator of the *bfp* operon	6.800	0.000
	*perB*; transcriptional regulator	4.115	0.000
	*perC*; transcriptional regulator	8.265	0.000
LEE pathogenicity island	*escC*; TTSS structure protein EscC	−2.219	0.004
	*escD*; TTSS structure protein EscD	−5.302	0.000
	*escR*; TTSS structure protein EscR	−2.969	0.000
	*escS*; TTSS structure protein EscS	−3.023	0.000
	*escV*; translocator EscV	−2.250	0.001
Acid resistance system	*gadA*; glutamate decarboxylase A, PLP-dependent	−9.294	0.000
	*gadB*; glutamate decarboxylase B, PLP-dependent	−13.065	0.000
	*gadC*; predicted glutamate: gamma-aminobutyric acid antiporter	−8.760	0.000

### 3.3. Depletion of *relA* and *spoT* leads to virulence attenuation of enteropathogenic *Escherichia coli* in a nematode model

**Figure 6:**
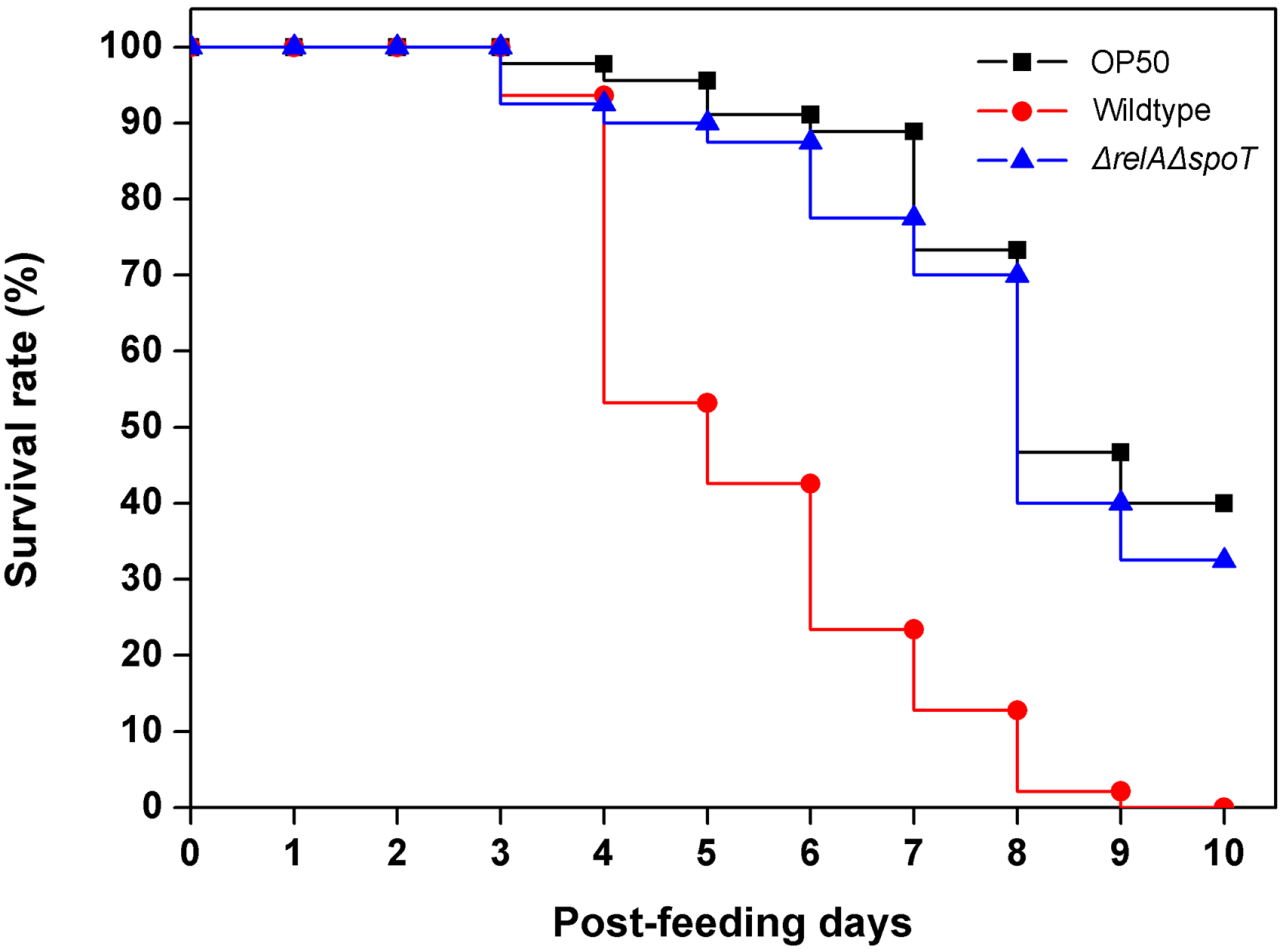
Attenuation of virulence in Δ*relA*Δ*spoT* EPEC. Virulence attenuation of the Δ*relA*Δ*spoT* mutant strain was analyzed by nematode killing assay. The wildtype (red circle) and Δ*relA*Δ*spoT* mutant (blue triangle) strains were seeded with synchronized *Caenorhabditis elegans*. Survival rates of *C. elegans* were then monitored for 10 days post-feeding. *E. coli* OP50 strain was used as a control (filled square).

To further assess the effect of ppGpp depletion on EPEC virulence, we designed an *in vivo* infection assay using *Caenorhabditis elegans*. Although there are no definitive animal models available for EPEC infection, a previous article has used *C. elegans* as an infection model for pathogenic bacteria (Couillault and Ewbank, 2002). Because other numerous bacterial infection studies have also used nematode as an infection host (Tan et al., 1999; Labrousse et al., 2000), we selected this organism as our host system. *C. elegans* were fed with *E. coli* OP50, a non-pathogenic strain known as a food source for nematodes, wildtype, and Δ*relA*Δ*spoT* mutant, respectively. Their survival ratios were then monitored up to 10 days. Approximately 50% of *C. elegans* fed with the wildtype survived on the fourth day. However, all died on the last day of observation. On the other hand, the death rate was significantly delayed in the group fed with the Δ*relA*Δ*spoT* mutant, similar to that in the group fed with OP50 (Figure 6). This result suggests that loss of ppGpp signal can significantly attenuate EPEC pathogenicity *in vivo*.

### 3.4. Infection by Δ*relA*Δ*spoT* enteropathogenic *Escherichia coli* stimulates expression of IL-6 in the host

After constructing the Δ*relA*Δ*spoT* mutant and assessing its effect on EPEC pathogenicity, we then aimed to identify host cell genes differentially expressed upon exposure to the Δ*relA*Δ*spoT* mutant. A porcine alveolar macrophage cell line 3D4/31 was infected with wildtype or Δ*relA*Δ*spoT* mutant and subjected to RNA-seq to analyze differentially expressed genes. Genes with their fold-change more than 2.0-fold and their normalized FPKM values over 30 were considered differentially expressed. The results revealed that 82 genes were differentially expressed in 3D4/31 infected with Δ*relA*Δ*spoT* mutant as compared to those in 3D4/31 infected with the wildtype. Among these genes, 77 genes were up-regulated, and five genes were down-regulated. Differentially expressed genes were categorized according to their functions. Notably, transcription factors and cytokine related genes accounted for a large proportion of up-regulated genes (Supplementary Table S4). To validate our transcriptome results, 11 genes were randomly selected and subjected to qRT-PCR. Expression patterns of 10 genes matched RNA-seq results (*value of p* <0.05; Supplementary Table S5), indicating that transcriptomic analyses derived from this work were highly valid. The datasets presented in this study can be found in the NCBI Sequence Read Archive^1^ under accession number PRJNA917101. Raw reads of the RNA-seq from two samples were deposited in the NCBI SRA^2^ under accession numbers SRR23329177 and SRR23329178.

RNA-seq analyses revealed increased expression levels of five proinflammatory cytokine genes (*IL-6*, *IL-8*, *GM-CSF*, *MCP-1*, and *MIP2-Α*) in 3D4/31 exposed to Δ*relA*Δ*spoT* mutant as compared to those in 3D4/31 exposed to the wildtype (Supplementary Table S6). Notably, IL-6 and IL-8 showed the highest fold increases (2.8- and 5.4-fold, respectively). Their expression levels were further assessed using qRT-PCR and ELISA (Table 4). Results showed that IL-6 and IL-8 expression at both transcription and protein levels were significantly increased in 3D4/31 cells infected with the Δ*relA*Δ*spoT* mutant when compared with those in 3D4/31 cells infected with the wild-type strain or the *relA-* and *spoT*-complemented mutant strain (Table 4).

**Table 4:** Increased expression levels of IL-6 and IL-8 in 3D4/31 infected with Δ*relA*Δ*spoT* EPEC.

Strain	Expression level of cytokines (mean ± SEM)			
	qRT-PCR (fold)		ELISA (pg/mL)	
	IL-6	IL-8	IL-6	IL-8
Mock control	1.0 ± 0.0	1.0 ± 0.0	70.5 ± 6.0	93.8 ± 6.7
Wildtype (pACYC)	3.8 ± 0.3	7.4 ± 0.3	112.2 ± 16.0	288.0 ± 42.3
Δ*relA*Δ*spoT* (pACYC)	27.9 ± 3.5^a^	69.4 ± 6.8^a^	641.5 ± 26.5^a^	1249.0 ± 141.2^a^
Δ*relA*Δ*spoT* (pACYC/SR)	1.7 ± 0.2	2.9 ± 0.0	104.6 ± 18.1	166.6 ± 14.9

a Values are significantly different from the wildtype and complemented groups (*p* < 0.001).

**Figure 7:**
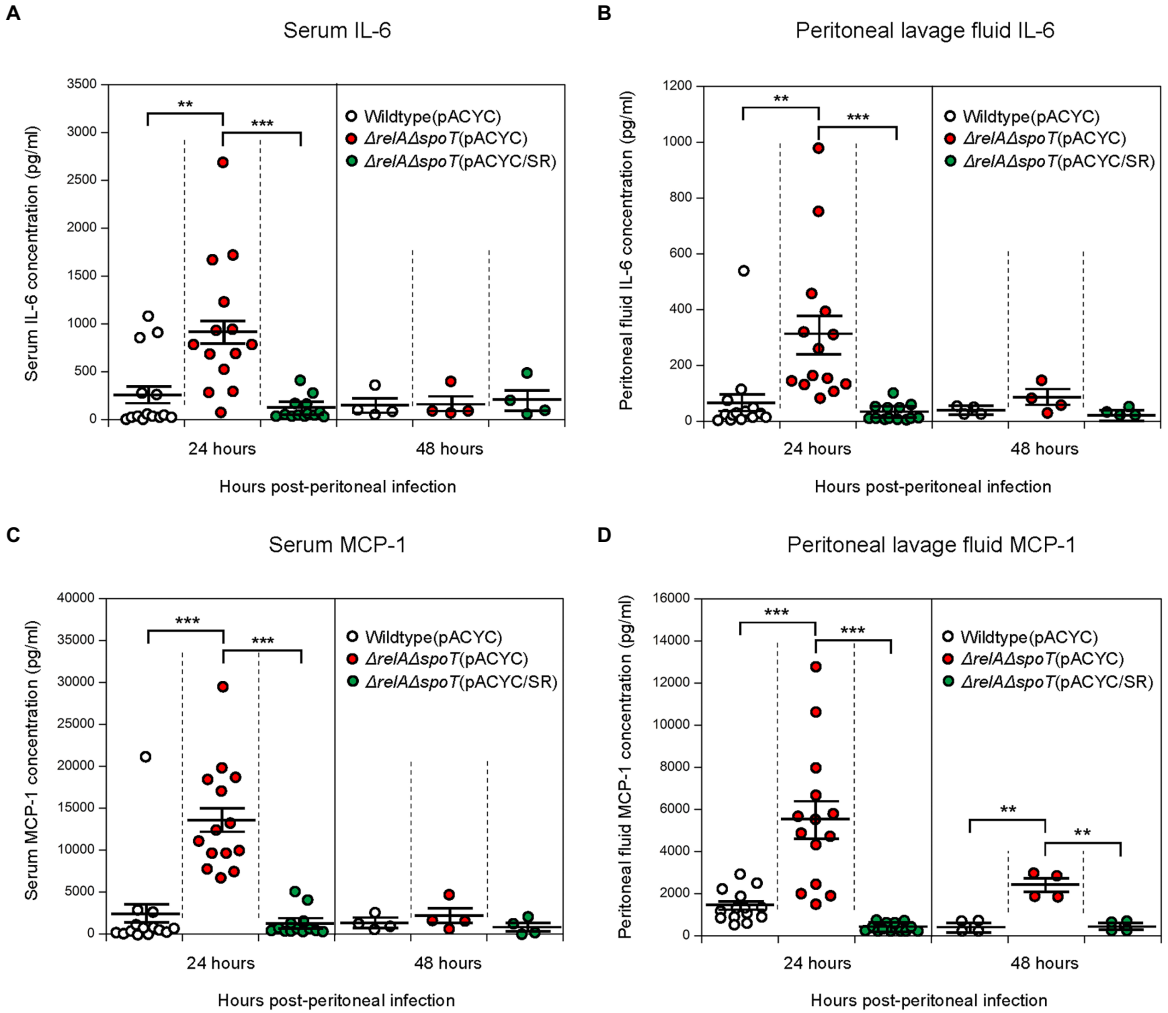
Increased expression of interleukin-6 (IL-6) and monocyte chemoattractant protein-1 (MCP-1) in C57BL/6 mice infected with Δ*relA*Δ*spoT* EPEC. C57BL/6 mice were challenged with wildtype (white), Δ*relA*Δ*spoT* mutant (red), or *relA-* and *spoT*-complemented mutant strain (green) *via* peritoneal injection. Samples were collected at 24 and 48 h post infection. **(A,B)** IL-6 concentration was quantified by enzyme-linked immunosorbent assay (ELISA) for blood serum **(A)** and lavage fluid **(B)** samples. Wildtype (pACYC), E2348/69 strain carrying pACYC184; Δ*relA*Δ*spoT* (pACYC), E2348/69 Δ*relA*Δ*spoT* mutant carrying pACYC184; Δ*relA*Δ*spoT* (pACYC/SR), E2348/69 Δ*relA*Δ*spoT* mutant carrying pACYC/SR. **(C,D)** MCP-1 concentration was also quantified by ELISA for serum **(C)** and lavage fluid **(D)** samples. Circles represent individual mice. Horizontal bars represent means ± standard errors of means. Statistical significance was obtained by Student’s *t*-test (**p < 0.01; ***p < 0.001).

We speculated that infection with the Δ*relA*Δ*spoT* mutant could also induce cytokine expression *in vivo*. To confirm this, we analyzed cytokine expression levels after EPEC infection using a murine peritoneal infection model. A single dose of 1 × 10^8^ CFU of wildtype, Δ*relA*Δ*spoT* mutant, or *relA-* and *spoT*-complemented mutant was injected intraperitoneally into each C57BL/6 N mouse. Blood and peritoneal lavage fluid samples were then taken at 24- and 48-h post infection and subjected to ELISA analysis using either IL-6 or MCP-1 antibody. At 24 h post infection, mice infected with the Δ*relA*Δ*spoT* mutant showed increased levels of IL-6 in both serum (Figure 7A) and lavage fluid (Figure 7B) samples in comparison with those infected with wildtype or complemented strain. At 48 h post infection, all groups had low levels of IL-6. The same expression patterns were observed for MCP-1 known to be controlled by IL-6 (Romano et al., 1997; Biswas et al., 1998) (Figures 7C,D). Collectively, these results suggest that infection with Δ*relA*Δ*spoT* EPEC can induce IL-6 expression both *in vitro* and *in vivo*.

### 3.5. Bacterial clearance is enhanced in mice infected with Δ*relA*Δ*spoT* enteropathogenic *Escherichia coli*

**Figure 8:**
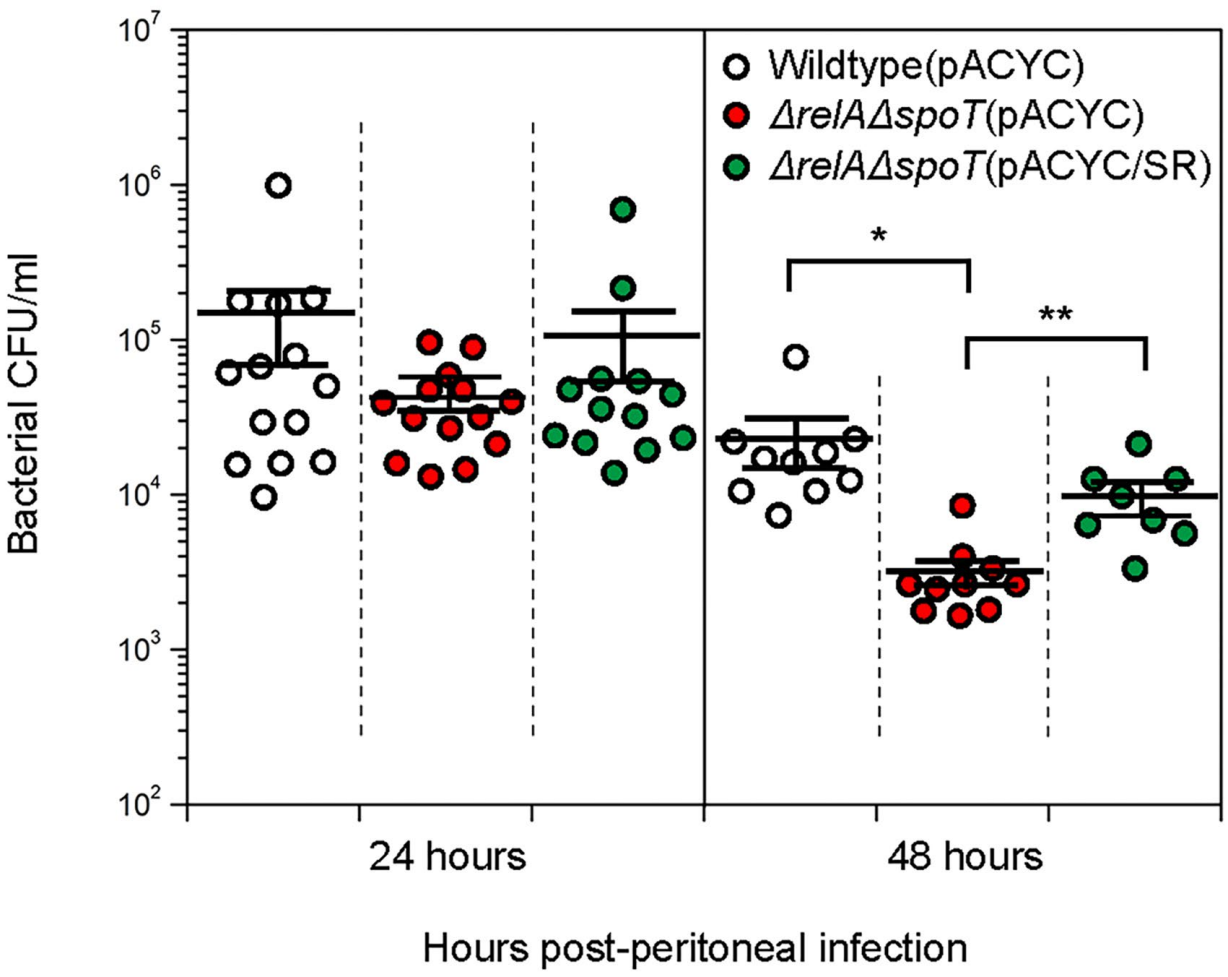
Improved bacterial clearance in C57BL/6 mice infected with Δ*relA*Δ*spoT* EPEC. C57BL/6 mice were challenged with wildtype (white), Δ*relA*Δ*spoT* mutant (red), or *relA-* and *spoT*-complemented mutant strain (green) *via* peritoneal injection. Bacterial colony forming units in peritoneal lavage fluids collected from C57BL/6 mice at 24 and 48 h post infection were counted by plating on Luria-Bertani agar plates supplemented with 5 μg/mL tetracycline. Circles represent individual mice. Horizontal bars represent means ± standard errors of means. Statistical significance was obtained by Student’s *t*-test (*p < 0.05; **p < 0.01). Wildtype (pACYC), E2348/69 strain carrying pACYC184; Δ*relA*Δ*spoT* (pACYC), E2348/69 Δ*relA*Δ*spoT* mutant carrying pACYC184; Δ*relA*Δ*spoT* (pACYC/SR), E2348/69 Δ*relA*Δ*spoT* mutant carrying pACYC/SR.

Cytokines play a vital role in early innate immune response. IL-6 and MCP-1 are known to contribute to bacterial clearance by inducing chemotaxis of neutrophils and macrophages (Itakura et al., 2017). We thus speculated that the number of Δ*relA*Δ*spoT* mutant survived in peritoneal lavage fluid would be significantly lower than that of wildtype or complemented strain *in vivo* due to increased levels of cytokines. To confirm this, we compared bacterial CFU in peritoneal lavage fluid samples by plating diluents on LB agar plates. At the 24 h timepoint, all EPEC strains showed similar numbers of survived cells. At the 48 h timepoint, however, the number of Δ*relA*Δ*spoT* mutant cells (3.2 ± 0.6 × 10^3^) was significantly reduced as compared to that of the wildtype (2.1 ± 0.7 × 10^4^) or the complemented strain (9.7 ± 2.1 × 10^3^) (Figure 8). This clearly indicates that bacterial clearance is favored in a host system that is exposed to Δ*relA*Δ*spoT* EPEC.

## 4. Discussion

Bacterial pathogens have developed various survival strategies that would enable them to thrive in a nutrient-limiting host system. Stringent response mediated by an alarmone ppGpp facilitates not only nutrient metabolism and scavenging, but also pathogenesis of infectious bacteria. Previous studies have shown effects of ppGpp deficiency on bacterial virulence factor production, metabolic dysfunctions, and virulence attenuation. However, little is known concerning changes in EPEC with Δ*relA*Δ*spoT* mutation. Here, we attempted to analyze the effect of ppGpp deficiency on EPEC pathogenesis during host infection.

One of the notable changes in EPEC by Δ*relA*Δ*spoT* mutation is its attenuated virulence. Both transcriptional and proteomic analyses revealed significant changes in type IV pilus assembly, acid resistance, and virulence factor secretion. BFP genes are involved in type IV pilus assembly. They are essential for initial adherence and microcolony formation of EPEC (Cleary et al., 2004). Our findings indicate that ppGpp can represses BFP expression. Such ppGpp-mediated negative effect on type IV pilus has also been observed in a plant pathogen, *Xanthomonas citri* (Zhang et al., 2019). Other studies have demonstrated that a poor nutrient condition known to induce ppGpp synthesis in bacteria can represses type IV pilus assembly. For example, *Pseudomonas aeruginosa* cells in the absence of nutrients can repress subcellular localization of FimX, which is required for type IV pilus assembly (Ni et al., 2016). This phenomenon allows nutrient-poor *P. aeruginosa* cells to remain dispersed, whereas nutrient-rich cells can form microcolonies on their niche spaces. Based on these results, we surmised that EPEC could reduce intracellular ppGpp levels on the small intestine where nutrients are sufficient, thereby increasing BFP expression. However, Spira et al. (2014) have reported that ppGpp favors BFP expression and that disrupting *relA* (but not *spoT*) would hinder type IV pilus assembly of EPEC. We do not have a clear explanation for such contradiction between ours and their study. However, we speculate that such difference might be due to serotype difference as we used O127:H6 E2348/69, whereas the previous report used O111ab:H2. Previous studies have shown that EPEC serotypes are different in their adherence pattern, EAF plasmid weight, and other phenotypes (Scotland et al., 1989; Okeke et al., 2001). Unknown factors derived from serotypic difference might have affected the outcome. Nonetheless, overall effects derived from ppGpp depletion suggest that ppGpp is deeply involved in early steps of EPEC colonization.

In contrast to BFP, LPI-encoded T3SS genes were repressed by Δ*relA*Δ*spoT* mutation, consistent with a previous report addressing the positive regulatory role of ppGpp in EHEC T3SS expression (Nakanishi et al., 2006). T3SS genes in both EPEC and EHEC are involved in intimate bacterial attachment on the intestinal surface (Galán and Collmer, 1999). For intimate attachment, EPEC can retract its type IV pilus to separate individual cells from microcolonies on the small intestine (Zahavi et al., 2011). Such dissociation of microcolonies permits intimate attachment, allowing translocation of effector proteins *via* T3SS. Since nutrient depletion caused by dense cell population in microcolonies can trigger ppGpp synthesis in bacteria (Mansour et al., 2016), we postulate that ppGpp could act as molecular switches to toggle between BFP and T3SS for the next step of the infectious process. In microcolonies, nutrient-poor EPEC cells could promote ppGpp synthesis and repress BFP expression, resulting the dispersal of cell aggregates. Subsequently, ppGpp-mediated T3SS expression allows EPEC cells to adhere onto intestinal epithelium intimately, creating an A/E lesion.

In addition to LPI-encoded T3SS genes, ppGpp is essential for expression of Pch, a transcriptional activator of LPI genes in EHEC (Iyoda and Watanabe, 2005). However, our observation showed that expression level of Per, an EPEC homologue of Pch (Mellies et al., 1999), was increased in Δ*relA*Δ*spoT* mutation. This result implies that activation of T3SS genes by ppGpp in EPEC can be induced by a Per-independent pathway. This coincides with Nakanishi’s work showing that Ler activation by ppGpp in EHEC is not *via* the expression of Pch (Nakanishi et al., 2006). Because ppGpp signaling induces a global transcriptional shift, we surmise that ppGpp could modulate unidentified factors to mediate Per-independent Ler activation and ultimately increase the expression of LPI genes in EPEC.

Other notable changes identified in this study include reduced expression of GadAB and decreased acid resistance of Δ*relA*Δ*spoT* EPEC. GadAB-mediated GDAR system is important for bacterial survival during host infection as EPEC must pass through acidic stomach to reach the small intestine (Hersh et al., 1996). Previously, Wells and Gaynor (2006) have reported that low pH condition could induce ppGpp accumulation in *Helicobacter pylori*. Based on these finding, we postulate that ppGpp is involved in pH adaptation of EPEC *via* the Gad system when exposed to an acidic environment.

In this study, we observed virulence attenuation of EPEC in *C. elegans* by Δ*relA*Δ*spoT* mutation. We also confirmed that virulence attenuation was not due to change in lipopolysaccharide (LPS) O-antigen structure (Supplementary Figure S2), a virulence determinant in case of *Salmonella* infection in *C. elegan*s (Aballay et al., 2003). Because the occurrence of virulence expression of EPEC is multifactorial (Chen and Frankel, 2005) and ppGpp-mediated regulation occurs in a global scale, it is difficult to pinpoint what might have caused the attenuation. One can argue that the attenuation is caused by decreased expression of LPI-encoded genes in the Δ*relA*Δ*spoT* EPEC. However, Mellies and his group suggested otherwise, as *Δler* mutant EPEC strain had similar *C. elegans* killing ability in comparison with the wildtype EPEC strain (Mellies et al., 2006). Likewise, both *ΔbfpA* mutant strain and EAF plasmid-cured strain caused similar lethality in *C. elegans* compared to wildtype strain, indicating that BFP and LEE were not related with EPEC-mediated *C. elegans* killing (Mellies et al., 2006). In addition to control of virulence factors from EPEC, ppGpp depletion might have triggered a protective immune response from the host organism. Since *C. elegans* has innate immune systems (Kim et al., 2002; Aballay et al., 2003), a Δ*relA*Δ*spoT* EPEC can boost immune response in *C. elegans*, ultimately improving its survival against the bacterial pathogen.

Many previous studies have demonstrated that EPEC can modulate host immune responses for its full virulence (Santos and Finlay, 2015). With its tissue tropism, EPEC can colonize the small intestine, specifically the duodenum, terminal ileum, and Peyer’s patches (Cantey and Inman, 1981; Fitzhenry et al., 2002). Since Peyer’s patches are surrounded by host immune cells, EPEC can induce acute immune responses as early as 12 h post infection (Inman and Cantey, 1984). However, it is unclear if ppGpp-mediated signaling in EPEC can affect host immune responses. In this study, both *in vitro* and *in vivo* immune responses towards wildtype and Δ*relA*Δ*spoT* mutant of EPEC were compared. Transcriptional analyses using 3D4/31 cells revealed that genes closely related to early immune response were significantly upregulated by Δ*relA*Δ*spoT* EPEC infection. These genes not only included pro-inflammatory cytokines, but also included transcription factors involved in immune responses. The most notable change was the boosting of a pro-inflammatory cytokine IL-6 in 3D4/31 cell when infected with Δ*relA*Δ*spoT* EPEC. Coinciding with results of *in vitro* analyses, *in vivo* murine infection tests also proved that IL-6 was induced for a short period and rapidly diminished when the host was challenged with Δ*relA*Δ*spoT* mutant. Bacterial clearance then followed afterward. IL-6 is considered as one of the acute-phase proteins whose concentrations are generally high on admission, but decrease rapidly after admission (Castell et al., 1989). A number of studies have shown the induction of IL-6 and its role during the early infection phase (Tanaka et al., 2014; Rose-John et al., 2017). Despite their vital roles in early innate immunity, prolonged expression of acute-phase proteins has adverse effects on host system, ultimately causing inflammatory damage. It can also exacerbate diseases and other bacterial inflictions. Some pathogens can take advantage of hypercytokinemia (Tisoncik et al., 2012). Therefore, anti-inflammatory regulation is critical for protecting the host system and defending against bacterial infection (Egan and Carding, 2000; Giamarellos-Bourboulis and Raftogiannis, 2012; Zhang and Wang, 2014). This ‘short’ period of IL-6 expression in C57BL/6 by Δ*relA*Δ*spoT* EPEC infection might reflect controlled inflammatory responses, combining both pro- and anti-inflammatory regulation to protect and maintain homeostasis in the host system, unlike the wild-type or the complemented strain where no apparent immune response was detected.

**Figure 9:**
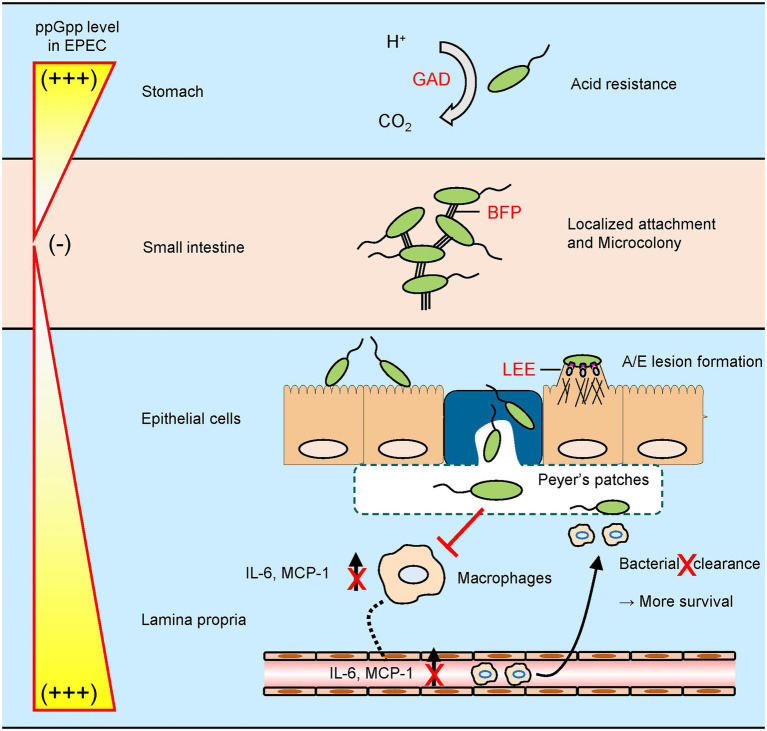
A schematic model for stringent control by EPEC during a host gastrointestinal tract infection. EPEC exhibits its virulence by controlling intracellular guanosine tetraphosphate (ppGpp) levels in response to host environments. (i) To survive in an acidic stomach, EPEC synthesizes ppGpp to induce glutamate decarboxylase-dependent acid resistance. (ii) After entering a nutrient-rich small intestine, EPEC degrades ppGpp to facilitate microcolony formation by type IV bundle forming pilus. (iii) Nutrient limitation caused by dense cell population in microcolony promotes ppGpp synthesis and dispersal of EPEC cell aggregates. Subsequently, ppGpp-mediated locus of enterocyte effacement expression allows EPEC cells to adhere onto intestinal epithelium intimately, creating attaching and effacing lesions. (iv) When exposed to macrophages in Peyer’s patches, EPEC deploys its ppGpp signaling to block both cytokine induction and subsequent bacterial clearance.

Based on findings of this study, we can postulate a schematic model to depict ppGpp-mediated signaling networks during EPEC pathogenesis in a host system (Figure 9). In general, EPEC cells enter host system *via* consumption of contaminated water and food and then colonize in human small intestine enterocytes. Upon arriving at the stomach, transcription of the *gad* operon would be activated by the increased ppGpp level in response to acid stress. This greatly enhances acid resistance. After passing through the stomach and entering the small intestine, nutrient-rich environment would prevent ppGpp accumulation and favor ppGpp degradation. Transcription of *perABC* and *bfp* operons would be derepressed. A chain of events would lead to successful EPEC attachment to the brush border. EPEC cells then aggregate to form microcolony due to increased BFP expression. The EPEC microcolony is broken as dense cell aggregates create a famine niche, favoring ppGpp synthesis. As intracellular ppGpp level rises, LEE operon genes are expressed, enabling EPEC cells to attach to the intestinal epithelium, creating A/E lesions. EPEC cells that are internalized through microfold cells can be recognized by macrophages residing in Peyer’s patches. In a general case, the interaction would not elicit a strong immune response as EPEC deploys its ppGpp-mediated signaling to evade host immune system. We believe that the stringent response could enable EPEC to successfully survive and thrive in the dynamic gastrointestinal tract environment. Some questions remain to be elucidated, such as characterization of unidentified factors enhancing IL-6 expression. Future studies will provide insights to stringent response associated regulation during EPEC pathogenesis.

Collectively, our findings reveal a role of ppGpp during EPEC pathogenesis and the impact it has on host immune response. Virulence attenuation by ppGpp deficiency in bacterial pathogens suggests various therapeutic applications. One instance is utilization of the Δ*relA*Δ*spoT* mutant strain as a live vaccine for immunization. Effects of immunization with pathogens defective in ppGpp synthesis have been described previously (Na et al., 2006; Dalebroux et al., 2010; Park et al., 2010). In this work, Δ*relA*Δ*spoT* EPEC showed potential as a promising live vaccine candidate as it elicited immune response in the host organism that was not observed after infection by the wild-type strain. However, the presented iteration needs to explain which bacterial factors are involved in such immune boosting effect of Δ*relA*Δ*spoT* EPEC. Further studies including a genomic-wide scale approach for identifying Δ*relA*Δ*spoT* EPEC proteins/factors eliciting innate immunity, its *in vivo* protective effect as a vaccine, and optimization of dosage and immunization methods for immune responses induction would greatly broaden our insights to EPEC pathogenesis and stringent response in the context of host immunity.

## Data availability statement

The original contributions presented in the study are included in the article/Supplementary material, further inquiries can be directed to the corresponding author.

## Ethics statement

The animal study was reviewed and approved by Kangwon National University Institutional Animal Care and Use Committees.

## Author contributions

JWY conceived and designed experiments and reviewed the manuscript. JBL was involved with all experiments and wrote the manuscript. SKK wrote and edited the manuscript. DH performed and validated the experiments. All authors contributed to the article and approved the submitted version.

## Funding

This study was supported by grants from National Research Foundation (NRF-2017R1A2B4013056 and NRF-022R1A6A3A01087483). This study was also supported in part by grant from Animal and Plant Quarantine Agency (Z-1543081-2021-23-01).

## Conflict of interest

The authors declare that the research was conducted in the absence of any commercial or financial relationships that could be construed as a potential conflict of interest.

## Publisher’s note

All claims expressed in this article are solely those of the authors and do not necessarily represent those of their affiliated organizations, or those of the publisher, the editors and the reviewers. Any product that may be evaluated in this article, or claim that may be made by its manufacturer, is not guaranteed or endorsed by the publisher.

## Footnotes

^1^ https://www.ncbi.nlm.nih.gov/bioproject

^2^ http://www.ncbi.nlm.nih.gov/Traces/sra
