# Supplementary material for: Mutating both relA and spoT of enteropathogenic Escherichia coli E2348/69 attenuates its virulence and induces interleukin 6 in vivo
Source: Front Microbiol. 2023 Mar 2;14:1121715. doi: 10.3389/fmicb.2023.1121715 (PMC10017862; doi:10.3389/fmicb.2023.1121715)
Supplement: Supplementary file 1 [file Data_Sheet_1.DOCX]

Supplementary Material

Mutating both *relA* and *spoT* of enteropathogenic *Escherichia coli* E2348/69 attenuates its virulence and induces interleukin 6 *in vivo*

Jun Bong Lee, Se Kye Kim, Dalmuri Han, Jang Won Yoon^*^

*** Correspondence:** Jang Won Yoon: jwy706@kangwon.ac.kr

**Supplementary Materials and Methods**

**Supplementary Figure 1.** **Sodium deoxycholate-polyacrylamide gel electrophoresis (DOC-PAGE) of lipopolysaccharide (LPS) extracts from wildtype and Δ*relA*Δ*spoT*** **EPEC.** LPS was extracted from overnight bacterial cultures using a traditional hot phenol method. Extracted LPS (10 mg) was loaded into each well as noted in the figure. Bands were visualized with silver staining.

**Supplementary Figure 2. Protein-protein interaction network created in Cytoscape software using STRING analysis performed on 17 differentially expressed proteins in Δ*relA*Δ*spoT*** **EPEC identified by matrix-assisted laser desorption/ionization time-of-flight (MALDI-TOF) analysis.** Circles filled with red indicate that a protein was upregulated in Δ*relA*Δ*spoT* EPEC. Circles filled with blue represent that a protein was downregulated in Δ*relA*Δ*spoT* EPEC. The depth of color is proportional to the absolute value of Log_2_fold changes between Δ*relA*Δ*spoT* EPEC and wildtype EPEC. The thickness of line represents confidence of the bonds. Bold lines represent strong correlation between two proteins.

**Supplementary Table 1.** **Bacterial strains and plasmids used in this study.**

**Supplementary Table 2. Oligonucleotides used in this study.**

**Supplementary Table 3. Differentially expressed genes in the Δ*relA*Δ*spoT* EPEC identified from microarray analysis (>2.0-fold).**

**Supplementary Table 4. Functional categorization of differentially expressed genes in 3D4/31 infected with the Δ*relA*Δ*spoT* EPEC.**

**Supplementary Table 5. Validation of transcriptional patterns in 3D4/31 infected with the Δ*relA*Δ*spoT* EPEC.**

**Supplementary Table 6. Differentially expressed cytokine genes in 3D4/31 infected with the *relA*Δ*spoT* EPEC.**

**Supplementary Materials and Methods**

**RNA sequencing**

RNA extraction method described in the manuscript was performed on three samples: One RNA sample of uninfected 3D4/31, one RNA sample of wildtype EPEC-infected 3D4/31, and one RNA sample of Δ*relA*Δ*spoT* EPEC-infected 3D4/31. The resulting RNA samples were sent to Macrogen (Seoul, Korea) and the quality of RNAs were assessed using an Agilent 2100 Bioanalyzer (Agilent Technologies, CA, USA). Only samples with an RNA integrity number (RIN) > 7.0 were used for the library preparation for *Sus scrofa*. cDNA libraries were prepared with total RNA using the Illumina TruSeq RNA Sample Preparation Kit (Illumina, Inc., CA, USA). The libraries were amplified and sequenced using an Illumina HiSeq 4000 (Illumina, Inc., CA, USA). Quality of raw reads were checked using FastQC v0.10.0 program (<http://www.bioinformatics.babraham.ac.uk/projects/fastqc/>). The low-quality bases with quality scores <30 and the maximum length of 35 base pair were removed by Trimmomatic 0.32 program (<http://www.usadellab.org/cms/?page=trimmomatic>). Each of the three libraries from 3D4/31 generated between 73.4 and 133.1 million reads pairs. More than 95.7% of the generated reads were mapped against the genomic DNA reference (GCF_000003025.6) using HISAT2 version 2.0.5 program (https://ccb.jhu.edu/software/hisat2/index.shtml). After read alignment, the StringTie version 1.3.3b software (https://ccb.jhu.edu/software/stringtie/) was used to assemble the aligned reads into transcripts and to measures the abundance of each transcript as fragments per kilobase of transcript per million mapped reads (FPKM). For differential expression gene (DEG) analysis, the values of log2 (FPKM+1) were calculated, and then normalized by quantile normalization. Expression of the transcript that satisfied with fold changes values larger than 2.0 were considered as DE genes.

**Enzymatic digestion of proteins in two-dimensional (2-DE) gel**

For protein identification, 20 protein spots of interest were cut off from the 2-DE gel, and each gel piece was washed with 50% acetonitrile (ACN) to remove residual sodium dodecyl sulfate (SDS), organic solvent, and staining reagents. The gel was then left to dry in room temperature, reswelled with trypsin (8-10 ng/µl), and incubated at 37℃ for 8-10 hours. Protein spots were enzymatically digested into small peptides by trypsin during incubation and protein digestion was terminated by adding 5 µl of 0.5% trifluoroacetic acid (TFA). Peptides cleaved by trypsin were resuspended in aqueous solution, desalted, and concentrated using C18 reversed phase resin-filled ziptip (Millipore, Herts, U.K.) to a volume of 1-5 µl. This concentrate was mixed with an equal volume of saturated solution of α-cyano-4-hydroxycinnamic acid (CHCA) in 50% ACN, and 1 µl of mixture was loaded onto a target plate for mass spectrometry.

**Matrix-assisted laser desorption/ionization time-of-flight (MALDI-TOF) analysis**

MALDI-TOF analysis was performed using Ettan MALDI-TOF Pro (Amersham Bioscience, NJ, USA). The ions were generated with a 337 nm nitrogen laser and accelerated to 20 kV injection pulse. Spectra of each protein spot were collected from 300 laser shots per spectrum and calibrated by two trypsin autodigestion ion peaks (m/z 842.510, 2211.1046). Calibrated spectra were submitted to National Center for Biotechnology Information (NCBI) databases using the ProFound peptide mass fingerprinting (PMF) software (<http://129.85.19.192/profound_bin/WebProFound.exe>) for protein identification. The parameters for database search were (i) trypsin as cleaving enzyme; (ii) a maximum of one missed cleavage; (iii) iodoacetamide (Cys) as a complete modification; (iv) methionine as a partial modification; (v) monoisotopic masses; and (vi) a mass tolerance of ±0.1 Da.
