# Supplementary figures and images for: Mutating both relA and spoT of enteropathogenic Escherichia coli E2348/69 attenuates its virulence and induces interleukin 6 in vivo
Source: Front Microbiol. 2023 Mar 2;14:1121715. doi: 10.3389/fmicb.2023.1121715 (PMC10017862; doi:10.3389/fmicb.2023.1121715)

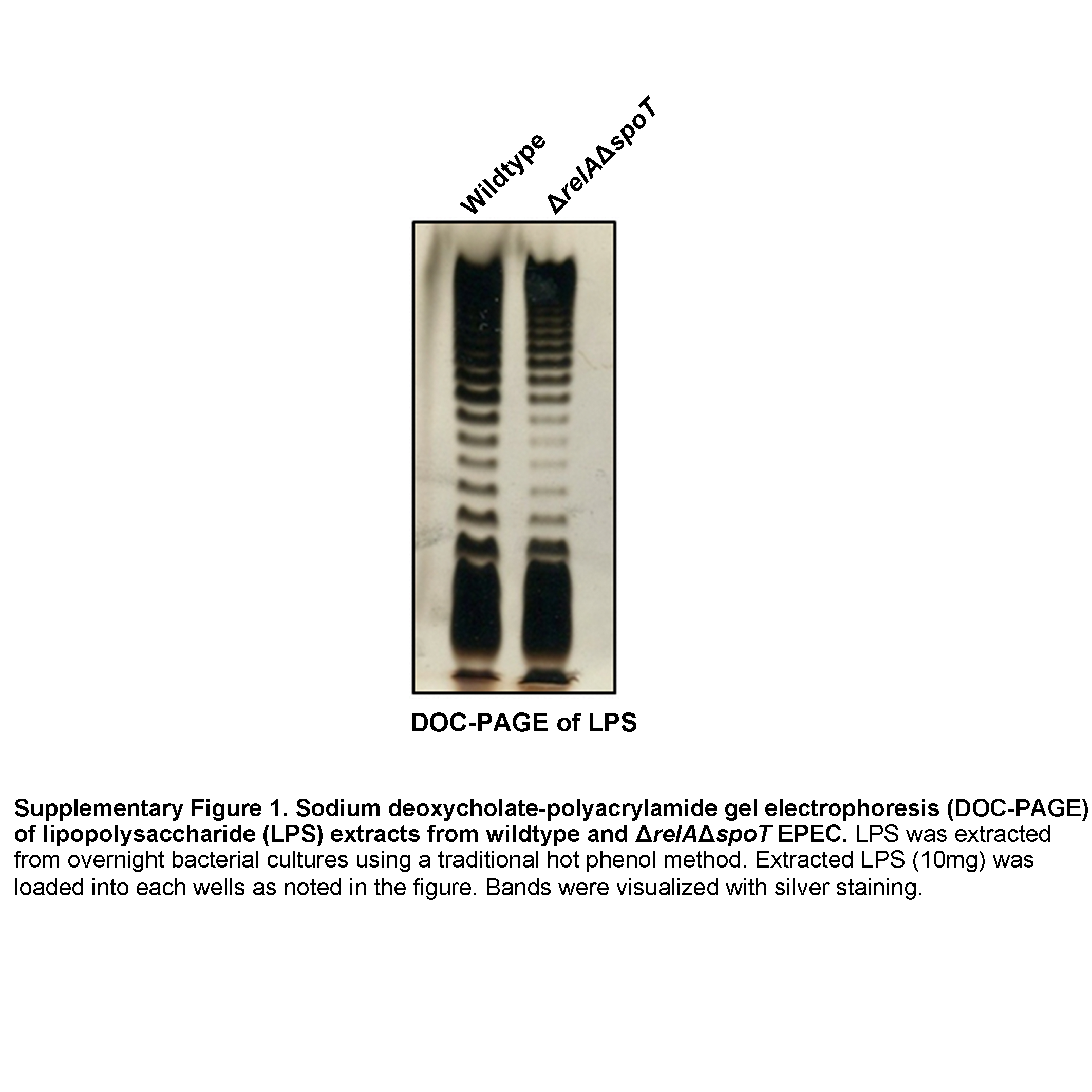

Supplement: Supplementary file 2 [file Image_1.TIFF]

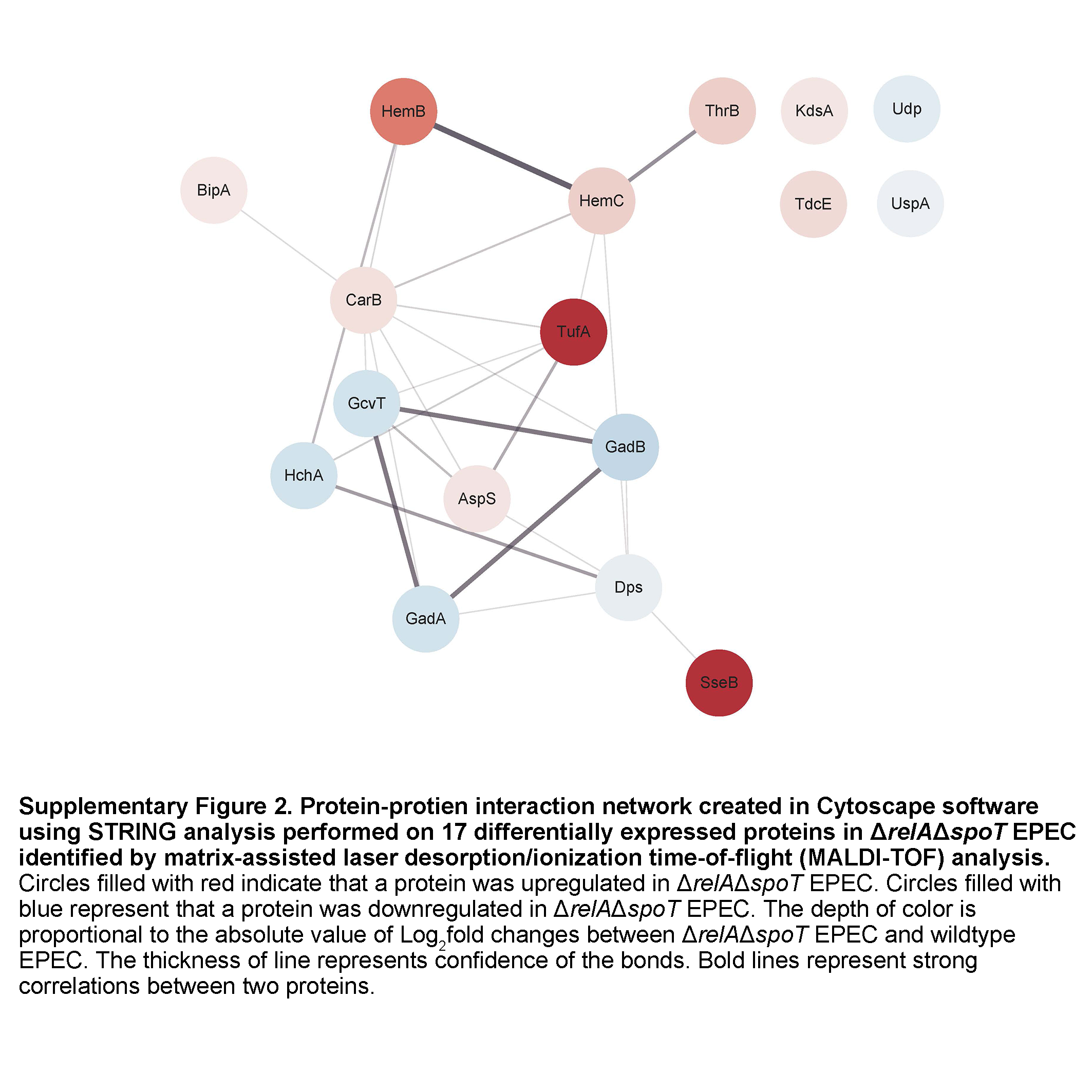

Supplement: Supplementary file 3 [file Image_2.TIFF]
