# Supplementary material for: Mutating both relA and spoT of enteropathogenic Escherichia coli E2348/69 attenuates its virulence and induces interleukin 6 in vivo
Source: Front Microbiol. 2023 Mar 2;14:1121715. doi: 10.3389/fmicb.2023.1121715 (PMC10017862; doi:10.3389/fmicb.2023.1121715)
Supplement: Supplementary file 4 [file Table_1.DOCX]

**Supplementary Table 1. Bacterial strains and plasmids used in this study.**

| Strain/plasmid | Description^a^ | Reference |
| --- | --- | --- |
| **Bacterial strains** | | |
| DH5α | *supE44* Δ*lacU169 (ф80 lacZ*Δ*M15) hsdR17 recA1 endA1 gyrA96 thi-1 recA1* | Lab stock |
| S17-1 λ*pir* | *pro* *recA* *thi* *hsdR* Hfr RP4-2 (Tc::*Mu*) (Km::Tn7) Sm^r^ Tp^r^ λ*pir* lysogen | Lab stock |
| E2348/69 | *E. coli* serotype O127:H6 E2348/69 strain | J. Kaper^b^ |
| E2348/69 Δ*relA*Δ*spoT* | E2348/69 Δ*relA::aphA-3* Δ*spoT::cat* (Km^r^ Cm^r^) | This study |
| E2348/69 (pUC19) | E2348/69 carrying pUC19 (Ap^r^) | This study |
| E2348/69 Δ*relA*Δ*spoT* (pUC19) | E2348/69 Δ*relA*Δ*spoT* carrying pUC19 (Km^r^ Cm^r^ Ap^r^) | This study |
| E2348/69 Δ*relA*Δ*spoT* (pUC19/S) | E2348/69 Δ*relA*Δ*spoT* carrying pUC19/S (Km^r^ Cm^r^ Ap^r^) | This study |
| E2348/69 (pACYC) | E2348/69 carrying pACYC184 (Cm^r^ Tc^r^) | This study |
| E2348/69 Δ*relA*Δ*spoT* (pACYC) | E2348/69 Δ*relA*Δ*spoT* carrying pACYC184 (Km^r^ Cm^r^ Tc^r^) | This study |
| E2348/69 Δ*relA*Δ*spoT* (pACYC/S) | E2348/69 Δ*relA*Δ*spoT* carrying pACYC/S (Km^r^ Cm^r^ Tc^r^) | This study |
| E2348/69 Δ*relA*Δ*spoT* (pACYC/SR) | E2348/69 Δ*relA*Δ*spoT* carrying pACYC/SR (Km^r^ Cm^r^ Tc^r^) | This study |
| **Plasmids** | | |
| pCVD442 | A conjugative suicide vector (Ap^r^) | J. Kaper^b^ |
| pUC19 | A multi-copy cloning vector (Ap^r^) | Promega |
| pACYC184 | A low copy cloning vector (Cm^r^ Tc^r^) | Lab stock |
| pUC19/S | Intact *spoT* from E2348/69 in pUC19 (Ap^r^) | This study |
| pACYC/S | Intact *spoT* from E2348/69 in pACYC184 (Tc^r^) | This study |
| pACYC/SR | Intact *spoT* and *relA* from E2348/69 in pACYC184 (Tc^r^) | This study |

^a^ Km^r^, kanamycin resistance; Ap^r^, ampicillin resistance; Cm^r^, chloramphenicol resistance; Tc^r^, tetracycline resistance; Sm^r^, streptomycin and spectinomycin resistance; Tp^r^, trimethoprim resistance.

^b^ A kind gift from Prof. J. Kaper at University of Maryland School of Medicine, U.S.A.
