# Supplementary material for: Mutating both relA and spoT of enteropathogenic Escherichia coli E2348/69 attenuates its virulence and induces interleukin 6 in vivo
Source: Front Microbiol. 2023 Mar 2;14:1121715. doi: 10.3389/fmicb.2023.1121715 (PMC10017862; doi:10.3389/fmicb.2023.1121715)
Supplement: Supplementary file 5 [file Table_2.DOCX]

**Supplementary Table 2. Oligonucleotides used in this study.**

| Target genes | Oligonucleotide sequence (5’ to 3’) | |
| --- | --- | --- |
|  | Forward primer | Reverse primer |
| *per* | GGGACATGGAAATTGTCGGAATCG | TGCATTTCATTGAGGTTCGCAGT |
| *bfpA* | TGATTGAATCTGCAATGGTG | AGCATTCTGCGACTTATTGG |
| *ler* | TGGGATATACTAATGTGCCTGATGA | ACCAGGTCTGCCCTTCTTCA |
| *sepD* | TGCAATATCCTGGCCGTTAG | AACGAGGCGAGAGTATGTTG |
| *escC* | CTCTCAGCCGTCAATTATAACCT | TCACTCTCAGCAATGTTCCG |
| *escV* | AGTGCTCGTTTTTCCCTTGA | AGCGAAGAACTTTTGCCTCA |
| *espA* | GCTGCAATTCTCATGTTTGC | GGGCAGTGGTTGACTCCTTA |
| *espB* | GCTCTGATTGGTGGTGCTAT | CCTGCCTTCTGTGCTAATTC |
| *eae* | GGCGATTACGCGAAAGATAC | GATTAACCTCTGCCGTTCCA |
| *tir* | GCAGAAGACGCTTCTCTGAATA | CCCAACTTCAGCATATGGATTA |
| *rrsB* | TGCAAGTCGAACGGTAACAG | AGTTATCCCCCTCCATCAGG |
| *TBP1* | AACAGTTCAGTAGTTATGAGCCAGA | AGATGTTCTCAAACGCTTCG |
| *IL-6* | AATGCTCTTCACCTCTCC | TCACACTTCTCATACTTCTCA |
| *IL-8* | CAGTTCTGGCAAGAGTAAGT | CCACTCTCAATCACTCTCAG |
| *GM-CSF* | CTGAGCCTTCTAAACAACAGT | CTCCTGGGGGTCAAACATT |
| *MCP-1* | TCACCTGCTGCTATACACTT | TCTTTAGGACACTTGCTGCT |
| *MIP-2A* | AAACGGAAGTCATAGCCACT | GTTGGCACTGCTCTTGTTTA |
| *HSPA8* | CCACGGAACAGGTCAGCAT | TCCAGCACTCAGGCCAGTAT |
| *PLAU* | ATCCCGCTCCATACAGAC | AGCTGCTCTGGATAGAGGTA |
| *AREG* | CCTCCAGAAGTGAGATGTCC | TATGTGCGGTTCGTTATCGT |
| *F3* | AAATGCTTCCACACGACAGAC | GTACGTCTCCTTCACGTTCCT |
| *MT-2B* | GCCTGAAGTTGGGGAGACC | TAGCAAACGGGTCAGGTTGTAT |
| *CEBPB* | TACAAGATCCGGCGTGAG | CAGCTGCTTGAACAAGTTCC |
| *SDC4* | AACATCTTCGAGAGGACAGAG | TTCTTCATCCGATACACCAGC |
| *ICAM1* | ATAATGCTGGCAGACGAGAA | GGCACTCTATGCTCACTGTA |
