# Supplementary material for: Mutating both relA and spoT of enteropathogenic Escherichia coli E2348/69 attenuates its virulence and induces interleukin 6 in vivo
Source: Front Microbiol. 2023 Mar 2;14:1121715. doi: 10.3389/fmicb.2023.1121715 (PMC10017862; doi:10.3389/fmicb.2023.1121715)
Supplement: Supplementary file 6 [file Table_3.PDF]

Supplementary Table 3. Differentially expressed genes in the ΔrelAΔspoT EPEC identified from microarray analysis (> 2.0-fold).

Probe ID	Name	Probe Comment	p-value (ΔrelAΔspoT vs. WT)	Fold changes (ΔrelAΔspoT vs. WT)	Standard deviation (ΔrelAΔspoT vs. WT)
9	215274587	-;probable plasmid replication initiation protein	0.03	-2.68	0.32
11	215276194	bfpA;major pilin structural unit	0.00	4.56	1.10
14	215276197	bfpC;hypothetical protein	0.01	2.08	0.44
18	215276201	bfpF;nucleotide binding protein	0.00	2.37	0.28
21	215276204	bfpI;prepilin	0.00	2.70	0.35
23	215276206	bfpK;prepilin	0.00	-3.99	0.77
25	215276210	perA;transcriptional activator of the bfp operon	0.00	6.80	2.16
26	215276211	perB;transcriptional regulator	0.00	4.11	1.54
27	215276212	perC;transcriptional regulator	0.00	8.26	0.95
29	215276214	-;hypothetical protein	0.01	2.27	0.13
31	215276216	copB;regulation of plasmid copy number	0.00	3.32	1.27
40	215276226	traT;lipoprotein	0.00	6.94	0.43
41	215276227	traS;membrane protein	0.00	5.12	0.67
47	215276233	traQ;pilin chaperone	0.02	-2.01	0.32
50	215276236	traF;putative disulfide-bond-forming protein	0.00	-2.39	0.35
51	215276237	trbE;hypothetical protein	0.01	-2.08	0.68
53	215276239	trbC;pilus assembly protein	0.00	-5.15	0.50
59	215276245	traV;pilus assembly lipoprotein	0.00	-2.83	0.22
63	215276249	traB;pilus assembly protein	0.02	-2.04	1.05
64	215276250	traK;pilus assembly protein	0.00	-2.28	0.36
67	215276253	traA;sex pilin subunit	0.01	-2.46	0.26
70	215276256	traM;putative signal transduction protein	0.00	2.84	0.46
72	215276258	-;hypothetical protein	0.00	-2.43	0.33
80	215276268	-;putative glutamate racemase	0.00	-13.12	0.92
81	215276269	-;transposase Orf1 of IS1N	0.00	2.35	0.90
83	215276271	stbB;plasmid stability protein	0.00	2.49	0.45
93	215276281	-;hypothetical protein	0.00	-2.41	0.20
95	215485162	thrL;thr operon leader peptide	0.01	-2.23	0.58
96	215485163	thrA;fused aspartokinase I/homoserine dehydrogenase I	0.01	-2.04	0.62
103	215485170	mog;predicted	0.00	3.34	0.30
107	215485175	dnaK;chaperone Hsp70, co-chaperone with DnaJ	0.01	-2.35	1.13
108	215485176	dnaJ;chaperone Hsp40, co-chaperone with DnaK	0.00	-3.56	0.04
109	215485177	mokC1;regulatory protein MokC for HokC 1	0.00	5.55	0.86
113	215485181	-;hypothetical protein	0.00	-2.10	0.53
114	215485182	nhaA;sodium-proton antiporter	0.00	-77.26	0.80
115	215485183	nhaR;DNA-binding transcriptional activator	0.00	-6.79	0.16
116	215485184	rpsT;30S ribosomal subunit	0.00	14.86	0.73
119	215485187	ileS;isoleucyl-tRNA synthetase	0.01	-2.25	0.13
125	215485193	carA;carbamoyl phosphate synthetase small subunit, glutamine amidotransferase	0.01	-2.15	0.87
128	215485196	caiF;DNA-binding transcriptional activator	0.00	-3.19	0.63
129	215485197	caiE;predicted acyl transferase	0.00	-2.28	0.21
140	215485208	kefF;flavoprotein subunit for the KefC potassium efflux system	0.00	-3.38	0.42
143	215485211	-;CcdA-like protein	0.00	-117.98	0.54
144	215485212	-;CcdB-like protein	0.00	-31.59	0.66
148	215485216	pdxA;4-hydroxy-L-threonine phosphate dehydrogenase,	0.01	-2.14	0.78
150	215485218	imp;exported protein required for envelope biosynthesis and	0.00	-3.86	0.31
153	215485221	hepA;RNA polymerase-associated helicase protein (ATPase and RNA polymerase	0.00	-4.72	0.51
159	215485227	-;predicted ribokinase	0.02	-2.58	0.78

160	215485228	–;predicted dihydrodipicolinate synthetase	0.00	−4.03	0.55
161	215485229	–;hypothetical protein	0.03	−3.51	0.96
162	215485230	–;hypothetical protein	0.00	−32.70	0.50
163	215485231	–;hypothetical protein	0.00	−11.52	0.84
164	215485232	yabI;conserved inner membrane	0.00	−13.86	1.03
176	215485244	ilvH;acetolactate synthase III, thiamin–dependent, small	0.00	−5.13	0.66
179	215485247	mraZ;hypothetical protein	0.00	2.23	0.26
180	215485248	mraW;S–adenosyl–dependent methyltransferase activity on membrane–located substrates	0.00	4.40	0.20
181	215485249	ftsL;membrane–bound cell division protein at septum containing leucine zipper motif	0.00	−2.92	0.73
183	215485251	murE;UDP–N–acetylmuramoyl–L–alanyl–D–glutamate: meso–diaminopimelate ligase	0.00	−2.38	0.38
184	215485252	murF;UDP–N–acetylmuramoyl–tripeptide: D–alanyl–D–alanine	0.00	−3.47	0.74
185	215485253	mraY;phospho–N–acetylmuramoyl–pentapeptide	0.00	−2.57	0.57
186	215485254	murD;UDP–N–acetylmuramoyl–L–alanine: D–glutamateligase	0.00	−5.25	0.25
188	215485256	murG;N–acetylglucosaminyl	0.00	−2.40	0.60
189	215485257	murC;UDP–N–acetylmuramate: L–alanine ligase	0.00	−2.19	0.78
192	215485260	ftsA;ATP–binding cell division protein FtsA involved in recruitment of FtsK to Z ring	0.00	2.40	0.32
193	215485261	ftsZ;GTP–binding tubulin–like cell division protein FtsZ	0.00	2.51	0.42
195	215485263	secM;regulator of SecA	0.00	2.84	0.41
199	215485267	yacF;hypothetical protein	0.00	4.05	0.29
200	215485268	coaE;dephospho–CoA kinase	0.00	2.53	0.18
201	215485269	guaC;GMP reductase	0.00	7.30	0.09
205	215485273	nadC;quinolinate phosphoribosyltransferase	0.00	2.61	0.14
206	215485274	ampD;N–acetyl–anhydromuranmyl–L–alanine	0.00	3.27	0.23
208	215485276	aroP;aromatic amino acid	0.00	−2.26	0.15
209	215485277	pdhR;DNA–binding transcriptional dual regulator	0.00	2.18	0.24
212	215485280	lpd;lipoamide dehydrogenase, E3 component is part of three enzyme complexes	0.00	2.28	0.46
218	215485286	yacC;hypothetical protein	0.00	3.55	0.94
220	215485288	gcd;glucose dehydrogenase	0.00	−4.27	0.34
222	215485290	can;carbonic anhydrase	0.00	7.92	0.38
224	215485292	yadH;predicted transporter subunit: membrane component of ABC superfamily	0.00	−2.59	0.07
225	215485293	yadI;predicted PTS Enzyme IIA	0.00	5.41	0.26
227	215485295	panD;aspartate 1–decarboxylase	0.00	9.11	0.54
228	215485296	yadD;predicted transposase	0.00	3.72	0.50
229	215485297	panC;pantothenate synthetase	0.01	−2.20	0.61
230	215485298	panB;3–methyl–2–oxobutanoate hydroxymethyltransferase	0.00	3.97	0.43
231	215485299	yadK;predicted fimbrial protein	0.00	−2.39	0.23
232	215485300	htrE;predicted outer membrane usher protein	0.00	−2.61	0.59
233	215485301	yadN;predicted fimbrial–like	0.00	−2.75	0.23
242	215485310	–;predicted fimbrial protein	0.00	2.81	0.43
245	215485313	fhuA;ferrichrome outer membrane transporter	0.00	−2.44	0.45
246	215485314	fhuC;iron–hydroxamate transporter subunit	0.00	3.32	0.58
249	215485317	hemL;glutamate–1–semialdehyde aminotransferase	0.00	3.03	0.52
251	215485319	yadR;hypothetical protein	0.00	4.88	0.58

252	215485320	yadS;conserved inner membrane protein	0.00	2.15	0.29
254	215485322	pfs;5'-methylthioadenosine/S-adenosylhomocysteine nucleosidase Pfs	0.00	3.89	0.25
257	215485325	cdaR;DNA-binding transcriptional activator	0.00	5.78	0.15
261	215485329	map;methionine aminopeptidase	0.00	4.37	0.31
265	215485333	frr;ribosome recycling factor	0.00	3.21	0.17
266	215485334	dxr;1-deoxy-D-xylulose 5-phosphate reductoisomerase	0.00	3.19	0.47
267	215485335	ispU;undecaprenyl pyrophosphate synthase	0.00	3.66	0.18
269	215485337	yaeL;zinc metalloprotease	0.01	2.18	0.19
277	215485345	dnaE;DNA polymerase III, alpha subunit	0.00	-3.01	0.28
278	215485346	accA;acetyl-CoA carboxylase, carboxytransferase, alpha	0.00	5.89	0.32
279	215485347	ldcC;lysine decarboxylase 2, constitutive	0.00	-2.24	0.04
281	215485349	tilS;tRNA(Ile)-lysidine synthetase	0.00	3.29	0.70
282	215485350	rof;modulator of Rho-dependent transcription termination	0.00	3.69	0.61
283	215485351	yaeP;hypothetical protein	0.01	2.13	0.45
284	215485352	yaeQ;hypothetical protein	0.00	3.98	0.17
285	215485353	yaeJ;hypothetical protein	0.00	3.70	0.19
290	215485358	rcsF;predicted outer membrane protein RcsF	0.00	6.22	0.34
293	215485361	metN;DL-methionine transporter subunit MetN, ATP-binding component of ABC superfamily	0.00	-2.46	0.29
294	215485362	gmhB;D,D-heptose 1,7-bisphosphate phosphatase	0.00	2.49	0.38
298	215485366	yafE;predicted S-adenosyl-L-methionine-dependent methyltransferase	0.01	-2.02	0.26
299	215485367	mltD;predicted membrane-bound lytic murein	0.00	7.28	0.80
300	215485368	gloB;predicted hydroxyacylglutathione hydrolase	0.00	2.57	0.44
302	215485370	rnhA;ribonuclease HI, degrades RNA of DNA-RNA hybrids	0.00	4.10	0.31
305	215485373	ivy;inhibitor of vertebrate C-	0.00	3.09	0.44
311	215485379	-;hypothetical protein	0.01	2.08	0.79
313	215485381	dinB;DNA polymerase IV	0.00	5.09	0.34
314	215485382	yafP;predicted acyltransferase with acyl-CoAN-acyltransferase	0.00	-2.78	0.42
315	215485383	-;hypothetical protein	0.00	-2.38	0.57
317	215485385	pepD;aminoacyl-histidine dipeptidase (peptidase D)	0.00	-2.26	0.52
318	215485386	gpt;guanine-hypoxanthine phosphoribosyltransferase	0.00	8.79	0.28
320	215485388	crl;DNA-binding transcriptional regulator	0.00	2.38	0.56
325	215485394	yagU;conserved inner membrane protein	0.00	-3.49	0.19
326	215485395	ykgJ;predicted ferredoxin	0.00	6.23	0.33
331	215485400	ecpA;fimbrillin precursor	0.01	2.06	0.26
332	215485401	ecpR;predicted regulator	0.00	3.35	0.99
338	215485407	-;predicted transcriptional	0.00	-2.06	0.38
340	215485409	-;predicted aldo/keto reductase	0.00	-2.27	0.38
347	215485416	ykgC;predicted oxidoreductase with FAD/NAD(P)-binding domain and dimerization domain	0.00	-4.39	0.62
348	215485417	ykgD;predicted DNA-binding transcriptional regulator	0.00	2.65	0.35
353	215485422	betA;choline dehydrogenase, flavoprotein	0.00	-3.33	0.88
354	215485423	betB;betaine aldehyde dehydrogenase, NAD-dependent	0.00	-2.56	0.65
356	215485425	betT;choline transporter of high	0.00	-2.69	0.47

362	215485431	yahF;predicted acyl-CoA synthetase with NAD(P)-binding domain and succinyl-CoA	0.00	-5.03	0.48
365	215485434	yahI;predicted carbamate kinase-like protein	0.01	-2.40	0.88
368	215485437	yahO;hypothetical protein	0.00	-2.55	0.73
370	215485439	prpB;2-methylisocitrate lyase	0.00	-4.91	1.30
371	215485440	prpC;2-methylcitrate synthase	0.01	-2.14	1.13
372	215485441	prpD;2-methylcitrate	0.00	-5.72	0.56
373	215485442	prpE;predicted propionyl-CoA synthetase with ATPase domain	0.00	-5.11	1.35
380	215485449	yaiL;nucleoprotein/polynucleotide-associated enzyme	0.00	2.21	0.34
382	215485451	frmA;alcohol dehydrogenase classIII/glutathione-dependent formaldehyde dehydrogenase	0.00	-3.31	0.32
384	215485453	tauA;taurine transporter subunit	0.00	-2.14	0.19
386	215485455	tauC;taurine transporter subunit TauC, membrane component of ABC superfamily	0.00	-2.24	1.00
388	215485457	hemB;porphobilinogen synthase	0.00	3.85	0.07
390	215485459	ampH;beta-lactamase/D-alanine carboxypeptidase	0.00	3.33	0.06
391	215485460	sbmA;predicted transporter	0.00	3.53	0.18
392	215485461	yaiY;predicted inner membrane	0.00	3.52	0.14
393	215485462	yaiZ;predicted inner membrane	0.00	2.36	0.76
394	215485463	ddlA;D-alanine-D-alanine ligase	0.00	2.16	0.13
395	215485464	iraP;hypothetical protein	0.00	3.31	1.18
399	215485468	proC;pyrroline-5-carboxylate reductase, NAD(P)-binding	0.00	3.10	0.50
404	215485473	yaiE;hypothetical protein	0.01	2.03	0.85
407	215485476	mak;manno (fructo) kinase	0.01	2.03	0.43
410	215485479	sbcD;ATP-dependent dsDNA exonuclease SbcD	0.02	2.04	0.53
413	215485482	brnQ;predicted branched chain amino acid transporter (LIV-II)	0.01	-2.06	0.37
416	215485485	acpH;hypothetical protein	0.00	2.50	0.28
418	215485487	tgt;tRNA-guanine	0.00	3.36	0.38
419	215485488	yajC;SecYEG protein translocase auxillary subunit	0.00	2.69	0.24
421	215485490	secF;SecYEG protein translocase auxillary subunit SecD	0.00	-2.45	0.07
422	215485491	yajD;hypothetical protein	0.00	2.37	0.51
423	215485492	tsx;nucleoside channel, receptor of phage T6 and colicin K	0.00	5.54	0.19
424	215485493	yajI;predicted lipoprotein	0.00	5.16	0.55
425	215485494	nrdR;hypothetical protein	0.01	2.21	0.70
426	215485495	ribD;fused diaminohydroxyphosphoribosylaminopyrimidine deaminase and 5-amino-6-(5-	0.00	2.28	0.37
428	215485497	nusB;transcription antitermination protein NusB	0.00	3.98	0.15
434	215485503	xseB;exonuclease VII small subunit XseB	0.00	5.18	0.41
439	215485508	yajR;predicted transporter	0.00	2.28	0.09
444	215485513	cyoA;cytochrome o ubiquinol oxidase subunit II	0.00	2.70	0.49
446	215485515	yajG;predicted lipoprotein	0.00	10.23	0.37
451	215485520	lon;DNA-binding ATP-dependent protease La	0.00	-2.33	0.15
452	215485521	hupB;HU, DNA-binding transcriptional regulator, beta	0.00	4.47	0.98
455	215485524	ybaW;hypothetical protein	0.00	3.25	0.76
456	215485525	queC;predicted aluminum resistance protein	0.00	3.71	0.32
458	215485527	cof;thiamin pyrimidine pyrophosphate hydrolase	0.00	-2.19	0.46
459	215485528	ybaO;predicted DNA-binding transcriptional regulator	0.00	2.48	0.28

461	215485530	mdlB;fused predicted multidrug transporter subunits of ABC superfamily: ATP-binding	0.01	-2.26	0.40
469	215485539	ylaC;predicted inner membrane	0.01	2.11	1.10
470	215485540	hha;modulator of gene expression, with H-NS	0.01	2.08	0.50
473	215485543	acrA;multidrug efflux system	0.00	3.05	0.13
474	215485544	acrR;DNA-binding transcriptional repressor	0.00	2.57	1.01
477	215485547	priC;primosomal replication	0.00	2.68	0.20
481	215485551	ybaB;hypothetical protein	0.00	-2.57	0.28
482	215485552	recR;gap repair protein RecR	0.01	-2.17	0.35
483	215485553	htpG;molecular chaperone HSP90 family	0.00	-2.85	0.73
484	215485554	adk;adenylate kinase	0.00	5.71	0.36
486	215485556	aes;acetyl esterase Aes	0.00	2.24	0.33
487	215485557	gsk;inosine/guanosine kinase	0.00	2.96	0.46
488	215485558	ybaL;predicted transporter with NAD(P)-binding Rossmann-fold domain	0.01	-2.19	0.95
491	215485561	ybaK;hypothetical protein	0.00	3.76	0.36
492	215485562	ybaP;hypothetical protein	0.00	2.31	0.74
494	215485564	copA;copper transporter	0.00	-3.36	0.38
495	215485565	ybaS;predicted glutaminase	0.00	-5.69	0.34
496	215485566	ybaT;predicted transporter	0.00	-2.96	0.60
499	215485569	-;predicted autotransporter adhesin, STEC autoagglutinating adhesin (Saa)-like protein	0.00	-2.28	0.86
501	215485571	ybbJ;conserved inner membrane protein	0.01	-2.14	0.33
502	215485572	ybbK;predicted protease, membrane anchored	0.00	-2.71	0.37
508	215485578	ybbA;predicted transporter subunit: ATP-binding component of ABC superfamily	0.00	-2.43	0.08
509	215485579	ybbP;predicted inner membrane	0.00	-3.55	0.69
510	215485581	ybbB;tRNA 2-selenouridine synthase, selenophosphate-	0.00	-3.77	0.61
512	215485583	allA;ureidoglycolate hydrolase	0.00	2.58	0.41
515	215485586	hyi;hydroxypyruvate isomerase	0.00	-5.03	0.35
516	215485587	glxR;tartronate semialdehyde reductase, NADH-dependent	0.00	-2.05	0.07
517	215485588	ybbW;predicted allantoin	0.01	-2.02	0.43
518	215485589	allB;allantoinase	0.00	-2.37	0.19
523	215485594	allD;ureidoglycolate	0.00	-3.91	0.30
528	215485599	purK;N5-carboxyaminoimidazole ribonucleotide synthase	0.00	-2.04	0.47
531	215485602	ppiB;peptidyl-prolyl cis-trans isomerase B (rotamase B)	0.01	2.06	0.49
532	215485603	cysS;cysteinyl-tRNA synthetase	0.00	2.71	0.74
534	215485605	ybcJ;predicted RNA-binding	0.00	3.99	0.33
536	215485607	-;hypothetical protein	0.00	2.47	0.25
538	215485609	ybcH;hypothetical protein	0.01	2.25	0.12
539	215485610	nfrA;bacteriophage N4 receptor, outer membrane subunit NfrA	0.00	-2.96	0.33
544	215485615	cusF;periplasmic copper-binding protein	0.01	-2.13	0.12
550	215485621	ybdF;hypothetical protein	0.00	2.88	0.18
551	215485622	ybdJ;predicted inner membrane	0.00	-3.00	0.33
557	215485628	fes;enterobactin/ferric enterobactin esterase	0.00	2.24	0.43
558	215485629	ybdZ;hypothetical protein	0.00	-3.39	1.06
559	215485630	entF;enterobactin synthase multienzyme complex component EntF, ATP-	0.00	-3.69	1.23
560	215485631	fepC;iron-enterobactin transporter subunit	0.00	3.91	1.16
561	215485632	fepG;ferric enterobactin transport protein FepG; membrane component of ABC superfamily	0.00	2.40	0.31
565	215485636	entC;isochorismate synthase 1	0.00	5.33	0.57

569	215485640	ybdB;hypothetical protein	0.00	-2.15	0.25
620	215485649	ahpC;alkyl hydroperoxide reductase, C22 subunit	0.00	-2.83	0.67
623	215485652	rnk;regulator of nucleoside diphosphate kinase	0.00	3.75	0.44
626	215485655	citG;triphosphoribosyl-dephospho-CoA transferase	0.00	-4.37	0.45
627	215485656	citX;apo-citrate lyase phosphoribosyl-dephospho-CoA	0.00	-4.55	0.18
633	215485662	citB;DNA-binding response regulator CitB in two-component regulatory system with CitA	0.02	2.15	0.48
636	215485665	cspE;DNA-binding transcriptional repressor	0.00	2.45	0.80
638	215485667	ybeM;predicted amidase	0.00	2.30	0.22
639	215485668	tatE;TatABCE protein translocation system subunit	0.01	2.20	0.89
640	215485669	lipA;lipoate synthase	0.00	2.59	0.21
641	215485670	lipB;lipoyl-protein ligase	0.00	3.22	0.24
653	215485682	leuS;leucyl-tRNA synthetase	0.00	-2.76	0.24
655	215485684	rihA;ribonucleoside hydrolase 1	0.00	3.54	0.28
663	215485692	ybeY;hypothetical protein	0.00	-3.91	0.40
664	215485693	ybeZ;conserved predicted protein with nucleoside triphosphate hydrolase domain	0.00	-3.28	0.37
667	215485696	asnB;asparagine synthetase B	0.01	-2.01	0.29
669	215485698	nagC;DNA-binding transcriptional dual regulator, repressor of the N-	0.00	-4.04	0.44
677	215485706	-;dihydrodipicolinate synthase family protein	0.00	-4.75	0.69
685	215485714	fur;DNA-binding transcriptional dual regulator of siderophore biosynthesis and transport	0.00	3.60	0.60
686	215485715	uof;ryhB-regulted fur leader	0.00	2.59	0.57
687	215485716	fldA;flavodoxin 1	0.00	6.51	0.27
688	215485717	ybfE;lexA-regulated predicted	0.00	10.17	0.65
690	215485719	seqA;regulatory protein for replication initiation SeqA	0.00	6.70	0.16
693	215485722	speF;ornithine decarboxylase isozyme SpeF, inducible	0.00	-5.91	0.50
696	215485725	kdpC;potassium translocating ATPase, subunit C	0.01	2.08	0.11
702	215485731	phr;deoxyribodipyrimidine photolyase, FAD-binding	0.00	-2.08	0.27
709	215485738	abrB;predicted regulator	0.00	-2.93	0.74
710	215485739	gltA;citrate synthase	0.01	2.10	0.19
711	215485740	sdhC;succinate dehydrogenase, membrane subunit, binds cytochrome b556	0.00	4.21	0.53
712	215485741	sdhD;succinate dehydrogenase, membrane subunit, binds cytochrome b556	0.00	4.71	0.33
714	215485743	sdhB;succinate dehydrogenase, FeS subunit	0.00	2.24	0.89
720	215485750	-;predicted glutamate mutase S	0.01	-2.17	0.17
723	215485754	cydA;cytochrome d terminal oxidase, subunit I	0.01	-2.29	0.76
724	215485755	cydB;cytochrome d terminal oxidase, subunit II	0.01	-2.28	0.36
727	215485758	ybgC;predicted acyl-CoA	0.00	7.18	0.47
728	215485759	tolQ;membrane spanning protein TolQ in TolA-TolQ-TolR complex	0.00	4.66	0.18
729	215485760	tolR;membrane spanning protein TolR in TolA-TolQ-TolR complex	0.00	3.21	0.26
738	215485769	aroG;3-deoxy-D-arabino-heptulosonate-7-phosphate synthase, phenylalanine	0.00	-2.63	0.27
743	215485774	-;UDP-galactose-4-epimerase	0.00	3.91	0.82
750	215485781	ybhA;predicted hydrolase	0.00	7.71	0.27
754	215485785	ybhI;predicted transporter	0.00	-2.33	0.78

756	215485787	ybhC;predicted pectin esterase	0.00	7.15	0.35
757	215485788	-;predicted integrase	0.00	4.58	0.14
758	215485790	-;hypothetical protein	0.00	7.25	0.54
759	215485791	-;hypothetical protein	0.00	5.46	0.27
760	215485793	-;hypothetical protein	0.00	3.27	0.19
761	215485795	-;hypothetical protein	0.00	5.88	0.31
762	215485798	-;hypothetical protein	0.00	6.21	0.29
763	215485799	-;predicted anti-RecBCD protein	0.00	6.77	0.21
764	215485804	-;hypothetical protein	0.01	2.08	0.16
765	215485806	-;hypothetical protein	0.00	2.95	0.23
766	215485807	-;hypothetical protein	0.00	5.30	0.29
767	215485808	-;predicted early gene regulator	0.00	6.52	0.40
768	215485809	-;hypothetical protein	0.00	3.37	0.40
770	215485811	-;predicted antirepressor protein	0.00	9.74	0.74
771	215485812	-;predicted regulatory protein	0.03	2.33	0.64
772	215485813	-;predicted replication protein	0.00	6.15	0.10
773	215485814	-;predicted replication protein	0.00	2.87	0.65
775	215485816	-;NinE	0.00	-2.73	0.48
776	215485817	-;NinF	0.00	4.75	0.25
777	215485818	-;predicted endonuclease	0.00	2.46	0.43
778	215485819	-;hypothetical protein	0.00	2.47	0.54
779	215485820	-;hypothetical protein	0.00	5.74	0.28
781	215485822	-;SfpA (systemic factor protein A)-like protein	0.00	2.83	0.41
782	215485823	-;predicted late gene regulator	0.01	2.06	0.98
783	215485825	-;hypothetical protein	0.00	2.85	0.87
790	215485833	-;predicted terminase small	0.02	2.08	0.22
792	215485836	-;predicted portal protein	0.00	-2.18	0.21
793	215485837	-;predicted head protein/prohead protease	0.00	-3.80	0.59
820	215485868	ybhK;predicted transferase with NAD(P)-binding Rossmann-fold domain	0.00	2.42	0.11
821	215485869	moaA;molybdopterin biosynthesis protein A	0.00	4.77	0.63
827	215485875	ybhM;conserved inner membrane protein	0.00	-3.87	0.47
829	215485877	ybhO;cardiolipin synthase 2	0.00	-3.81	0.09
831	215485879	ybhQ;predicted inner membrane	0.00	4.84	0.76
834	215485882	ybhF;fused predicted transporter subunits of ABC superfamily: ATP-binding components	0.00	-2.10	0.38
837	215485885	rhlE;RNA helicase	0.03	-2.05	1.19
845	215485893	ybiM;hypothetical protein	0.00	-3.36	0.82
847	215485895	ybiO;predicted mechanosensitive channel	0.00	-2.84	0.11
848	215485896	glnQ;glutamine transporter	0.00	4.05	0.38
849	215485897	glnP;glutamine transporter	0.00	-2.65	0.23
850	215485898	glnH;glutamine transporter	0.00	4.13	0.74
855	215485904	mntR;DNA-binding transcriptional regulator of mntH	0.00	2.79	0.19
856	215485905	ybiR;predicted transporter	0.00	2.54	0.30
868	215485917	gsiC;predicted peptide transporter subunit: membrane component of ABC superfamily	0.01	-2.24	0.50
871	215485920	yliF;predicted diguanylate	0.01	-2.26	0.59
884	215485933	-;predicted integrase	0.00	2.78	0.32
887	215485936	-;predicted repressor protein	0.00	3.77	0.81
892	215485941	-;hypothetical protein	0.00	-2.58	0.34
900	215485949	-;hypothetical protein	0.00	3.75	0.31
903	215485952	-;predicted terminase, ATPase	0.02	-2.44	0.47
906	215485955	-;predicted terminase, endonuclease subunit	0.00	-2.25	0.69
910	215485959	-;predicted lysozyme	0.01	-2.02	0.43
911	215485960	-;predicted membrane protein	0.00	-2.80	0.95
912	215485961	-;predicted regulatory protein	0.00	-2.81	0.22
914	215485963	-;predicted variable tail fibre	0.01	-2.01	0.26
915	215485964	-;predicted tail protein	0.02	-2.43	0.37
920	215485969	-;predicted DNA invertase	0.00	3.15	0.07
925	215485974	-;predicted tail tape measure	0.00	-2.11	0.17

931	215485980	grxA;glutaredoxin 1, redox coenzyme forribonucleotide	0.00	5.18	0.76
932	215485981	ybjC;predicted inner membrane	0.00	2.84	0.94
933	215485982	nfsA;nitroreductase A, NADPH-dependent, FMN-dependent	0.00	2.22	0.18
935	215485984	ybjN;predicted oxidoreductase	0.00	4.36	0.35
936	215485985	potF;putrescine transporter subunit PotF: periplasmic-binding component of ABC	0.00	-2.64	0.54
937	215485986	potG;putrescine transporter subunit PotG: ATP-binding component of ABC superfamily	0.00	-2.39	0.64
938	215485987	potH;putrescine transporter subunit PotH: membrane component of ABC superfamily	0.00	-3.89	0.08
949	215485998	ybjR;predicted amidase and	0.00	-3.12	0.50
952	215486001	ltaE;L-allo-threonine aldolase, PLP-dependent	0.00	-3.60	0.17
953	215486002	poxB;pyruvate dehydrogenase (pyruvate oxidase), thiamin-dependent, FAD-binding	0.00	-3.61	0.14
954	215486003	hcr;HCP oxidoreductase, NADH-dependent	0.00	-4.67	0.57
960	215486009	macA;macrolide transporter subunit, membrane fusionprotein (MFP) component	0.00	-2.15	0.15
961	215486010	macB;fused macrolide transporter subunits of ABC superfamily: ATP-binding component/membrane	0.00	-4.69	0.46
964	215486013	clpA;ATPase and specificity subunit of ClpA-ClpP ATP-dependent serine protease,	0.00	-2.45	0.33
965	215486014	infA;translation initiation factor	0.00	10.64	1.30
969	215486018	trxB;thioredoxin reductase, FAD/NAD(P)-binding	0.00	3.82	0.20
971	215486020	ftsK;DNA-binding membrane protein FtsK required for chromosome resolution and	0.00	-6.88	0.57
973	215486022	rarA;recombination protein	0.00	-2.46	0.33
976	215486025	dmsC;dimethyl sulfoxide reductase, anaerobic, subunit C	0.00	-2.27	0.21
978	215486027	pflA;pyruvate formate lyase activating enzyme 1	0.01	2.05	0.58
979	215486028	pflB;pyruvate formate lyase I	0.00	-3.23	0.78
981	215486030	ycaO;hypothetical protein	0.00	-3.01	0.76
982	215486031	ycaP;conserved inner membrane protein	0.00	3.21	0.38
984	215486033	aroA;5-enolpyruvylshikimate-3-phosphate synthetase	0.00	-3.08	0.20
985	215486034	ycaL;predicted peptidase with chaperone function	0.00	2.73	0.71
986	215486035	cmk;cytidylate kinase	0.00	4.72	0.48
994	215486043	kdsB;3-deoxy-manno-octulosonate cytidylyltransferase	0.00	2.24	0.29
995	215486044	ycbJ;hypothetical protein	0.00	-2.24	0.17
997	215486046	smtA;predicted S-adenosyl-L-methionine-dependent methyltransferase	0.00	2.69	0.58
998	215486047	mukF;MukK protein involved in chromosome partioning, Ca2+ binding protein	0.00	-3.03	0.20
1001	215486050	ycbB;predicted carboxypeptidase	0.00	-2.71	0.36
1002	215486051	ycbK;hypothetical protein	0.00	2.61	0.62
1003	215486052	ycbL;predicted metal-binding	0.00	4.35	0.32
1004	215486053	aspC;aspartate aminotransferase, PLP-	0.00	6.61	0.06
1007	215486056	pncB;nicotinate phosphoribosyltransferase	0.00	4.16	0.69
1008	215486057	pepN;aminopeptidase N	0.00	-5.26	0.79

1010	215486059	ssuC;alkanesulfonate transporter subunit, membrane component of ABC superfamily	0.00	-2.25	0.20
1011	215486060	ssuD;alkanesulfonate monooxygenase, FMNH(2)-	0.03	-2.07	0.36
1015	215486064	ycbW;hypothetical protein	0.00	2.72	0.82
1021	215486070	ymbA;hypothetical protein	0.00	2.17	0.23
1023	215486072	fabA;beta-hydroxydecanoyl thioester dehydrase	0.00	3.93	0.35
1024	215486073	ycbZ;predicted peptidase	0.00	-2.51	0.48
1025	215486074	ycbG;hypothetical protein	0.00	2.87	0.95
1028	215486077	yccR;hypothetical protein	0.00	2.74	0.88
1030	215486079	yccF;conserved inner membrane protein	0.00	2.71	0.58
1031	215486080	helD;DNA helicase IV	0.00	-2.27	0.19
1032	215486081	mgsA;methylglyoxal synthase	0.00	9.34	0.45
1033	215486082	yccT;hypothetical protein	0.00	-2.97	0.42
1038	215486087	yccK;predicted sulfite reductase subunit	0.00	4.08	0.62
1040	215486089	hyaA;hydrogenase 1, small	0.01	-2.13	0.45
1041	215486090	hyaB;hydrogenase 1, large	0.00	-2.86	0.55
1042	215486091	hyaC;hydrogenase 1, b-type cytochrome subunit	0.00	-2.87	0.18
1043	215486092	hyaD;HyaD protein involved in processing of HyaA and HyaB	0.00	-3.59	0.58
1044	215486093	hyaE;HyaE protein involved in processing of HyaA and HyaB	0.01	-2.03	0.26
1046	215486095	appC;cytochrome bd-II oxidase, subunit I	0.00	-3.53	0.11
1047	215486096	appB;cytochrome bd-II oxidase, subunit II	0.00	-5.03	0.28
1052	215486101	gfcD;hypothetical protein	0.01	-2.32	0.21
1054	215486103	gfcB;predicted outer membrane lipoprotein	0.00	4.53	0.29
1056	215486105	cspH;stress protein, member of the CspA-family	0.00	5.96	1.14
1058	215486107	ymcE;Sfa protein suppresses fabA and ts growth mutation	0.00	-2.95	0.75
1059	215486108	gnsA;predicted regulator of phosphatidylethanolamine	0.00	2.49	1.01
1063	215486112	-;predicted excisionase	0.00	3.19	0.63
1064	215486113	-;hypothetical protein	0.00	2.20	0.30
1067	215486116	-;hypothetical protein	0.01	2.11	0.57
1068	215486117	-;predicted anti-RecBCD protein	0.01	2.14	0.17
1070	215486120	-;hypothetical protein	0.00	2.37	0.29
1072	215486124	-;predicted single-stranded DNA binding protein	0.00	3.25	0.56
1080	215486132	-;predicted replication protein	0.01	-2.20	0.12
1083	215486135	-;NinB	0.01	2.18	0.88
1086	215486138	-;NinF	0.00	3.03	0.02
1092	215486144	-;predicted membrane protein	0.00	2.33	0.30
1093	215486145	-;hypothetical protein	0.00	-3.39	0.31
1094	215486146	-;predicted holin protein	0.00	-2.40	0.54
1098	215486150	-;hypothetical protein	0.00	-3.45	0.36
1100	215486152	-;predicted terminase large	0.00	-3.72	0.29
1107	215486159	-;predicted minor tail protein	0.00	-4.15	0.34
1118	215486171	-;T3SS effector-like protein NleB homolog	0.00	2.90	0.90
1119	215486172	-;T3SS secreted effector NleC	0.00	2.31	0.34
1126	215486179	cbpM;modulator of CbpA co-chaperone	0.00	-3.66	0.70
1128	215486181	agp;glucose-1-phosphatase/inositol	0.00	-4.57	0.63
1129	215486183	yccJ;hypothetical protein	0.02	-2.06	0.26
1130	215486184	wrbA;predicted flavoprotein in Trp regulation	0.00	-2.82	0.19
1132	215486186	rutG;predicted transporter	0.00	-5.71	0.92
1137	215486191	rutB;predicted enzyme	0.01	-2.06	1.16
1141	215486195	putP;proline: sodium symporter	0.01	2.03	0.32
1142	215486196	efeU;predicted high-affinity Fe2+/Pb2+ permease	0.01	2.03	0.51

1147	215486201	–;predicted integrase	0.00	2.16	0.22
1149	215486203	–;hypothetical protein	0.00	–7.03	0.81
1150	215486204	–;hypothetical protein	0.00	–3.13	0.42
1151	215486205	–;hypothetical protein	0.00	–2.26	0.25
1152	215486206	–;Efa1/LifA–like protein	0.02	–2.08	0.57
1154	215486208	–;predicted transposase	0.01	–2.03	0.12
1155	215486211	–;transposase Orf1 of IS100	0.02	–2.06	0.40
1156	215486212	–;transposase Orf2 of IS100	0.01	2.22	0.39
1157	215486213	–;hypothetical protein	0.01	–2.29	0.21
1158	215486214	–;predicted type I site–specific deoxyribonuclease, HsdR family	0.00	–3.08	0.53
1159	215486215	–;hypothetical protein	0.00	–2.95	0.44
1161	215486217	–;predicted type I restriction–modification enzyme, S subunit	0.00	–2.96	0.41
1164	215486220	–;hypothetical protein	0.00	4.12	0.56
1168	215486224	–;transposase Orf2 of ISEc17	0.00	–5.06	1.31
1172	215486228	–;transposase Orf1 of IS629	0.00	2.48	0.26
1173	215486229	–;hypothetical protein	0.00	–5.66	0.16
1174	215486230	–;hypothetical protein	0.00	–5.88	0.26
1175	215486231	–;hypothetical protein	0.00	–2.33	1.07
1179	215486235	–;hypothetical protein	0.00	–2.59	0.33
1182	215486238	–;hypothetical protein	0.05	–2.17	0.81
1186	215486242	ghrA;2–ketoacid reductase	0.00	4.13	0.42
1187	215486243	ycdX;predicted zinc–binding	0.00	2.84	0.14
1189	215486245	ycdZ;predicted inner membrane	0.00	2.31	0.45
1190	215486246	csgG;outer membrane	0.00	–3.58	0.34
1203	215486259	mdoH;glucan biosynthesis MdoH: glycosyl transferase	0.00	–6.85	0.26
1205	215486261	msyB;acidic protein MsyB	0.00	2.78	0.31
1208	215486264	yceA;hypothetical protein	0.00	7.46	0.34
1212	215486268	solA;N–methyltryptophan oxidase, FAD–binding	0.01	–2.15	0.59
1213	215486269	bssS;conserved predicted protein that regulates biofilm	0.02	2.01	1.05
1214	215486270	dinI;DNA damage–inducible	0.00	2.49	0.99
1215	215486271	pyrC;dihydro–orotase	0.00	3.59	0.50
1216	215486272	yceB;predicted lipoprotein	0.00	5.20	0.17
1217	215486273	grxB;glutaredoxin 2 (Grx2)	0.00	–8.53	0.32
1233	215486289	flgI;predicted flagellar basal body protein	0.01	–2.02	0.33
1239	215486295	rne;fused ribonuclease E: endoribonuclease/RNA–binding protein/RNA degradosome binding protein	0.00	–2.86	0.37
1242	215486298	yceF;hypothetical protein	0.00	2.79	0.13
1255	215486311	ycfH;predicted metallodependent hydrolase	0.00	2.42	0.52
1264	215486320	ndh;respiratory NADH dehydrogenase	0.00	2.44	0.61
1265	215486321	ycfJ;hypothetical protein	0.00	2.72	0.70
1266	215486322	ycfQ;predicted DNA–binding transcriptional regulator	0.00	2.66	0.66
1269	215486325	mfd;transcription–repair coupling	0.00	–2.52	0.35
1272	215486328	lolD;outer membrane–specific lipoprotein transporter subunit	0.00	–3.05	0.46
1276	215486332	ycfZ;predicted inner membrane	0.01	–2.18	0.13
1279	215486335	potC;polyamine transporter subunit PotC	0.00	4.21	0.45
1280	215486336	–;predicted integrase	0.00	2.19	0.40
1295	215486351	–;predicted ATP–binding protein	0.00	–3.39	0.15
1296	215486352	–;predicted HNH endonuclease	0.01	–2.06	1.66
1298	215486354	–;hypothetical protein	0.00	4.62	0.86
1299	215486355	–;hypothetical protein	0.01	2.15	0.43
1301	215486357	–;predicted crossover junction endodeoxyribonuclease	0.00	–2.96	0.79
1304	215486360	–;predicted DNA methylase	0.00	–2.27	0.20
1305	215486361	–;hypothetical protein	0.00	2.40	1.02
1309	215486367	–;predicted endopeptidase	0.03	2.06	0.45
1318	215486376	–;predicted outer membrane precursor Lom	0.02	–2.03	0.18
1320	215486378	–;hypothetical protein	0.01	2.03	0.60

1324	215486382	ycfD;hypothetical protein	0.00	3.94	0.32
1325	215486383	phoQ;sensory histidine kinase in two-component regulatory system with PhoP	0.00	-3.95	0.65
1329	215486387	mnmA;tRNA (5-methylaminomethyl-2-	0.00	4.06	0.24
1331	215486389	rluE;23S rRNA pseudouridine	0.00	3.37	0.31
1334	215486392	ycgE;predicted DNA-binding transcriptional regulator	0.00	3.95	0.57
1337	215486395	ymgA;hypothetical protein	0.00	-3.71	0.91
1340	215486398	ymgF;hypothetical protein	0.00	4.10	0.98
1341	215486399	ymgD;hypothetical protein	0.00	4.29	0.91
1342	215486400	ymgG;hypothetical protein	0.00	3.42	0.14
1343	215486401	ymgI;hypothetical protein	0.00	5.96	0.43
1344	215486402	ymgJ;coserved predicted protein	0.00	-3.02	0.45
1345	215486403	minE;cell division topological specificity factor MinE	0.00	2.58	0.46
1346	215486404	minD;membrane ATPase MinD of the MinC-MinD-MinE system	0.00	2.66	0.35
1347	215486405	minC;cell division inhibitor MinC	0.00	2.88	0.48
1348	215486406	ycgJ;hypothetical protein	0.00	2.65	0.67
1353	215486411	umuD;DNA polymerase V,	0.00	2.57	0.16
1355	215486413	dsbB;oxidoreductase that catalyzes reoxidation of DsbA protein disulfide isomerase I	0.00	3.58	0.46
1357	215486415	fadR;DNA-binding transcriptional dual regulator of fatty acid	0.00	3.66	0.15
1358	215486416	ycgB;hypothetical protein	0.00	-4.59	0.45
1362	215486420	ldcA;L,D-carboxypeptidase A	0.00	2.78	0.49
1363	215486421	emtA;lytic murein endotransglycosylase E	0.00	2.23	0.49
1365	215486423	ymgE;predicted inner membrane	0.00	-2.33	0.78
1367	215486425	-;molybdenum transport protein	0.01	-2.05	0.48
1369	215486427	-;predicted ferric enterobactin transport ATP-binding protein	0.00	-2.59	1.04
1377	215486435	ychF;predicted GTP-binding	0.00	3.69	0.30
1378	215486436	pth;peptidyl-tRNA hydrolase	0.00	5.63	0.20
1381	215486439	prs;phosphoribosylpyrophosphate synthase	0.00	3.04	0.63
1384	215486442	hemA;glutamyl tRNA reductase	0.00	2.93	0.09
1388	215486446	ychA;predicted transcriptional	0.00	4.30	0.05
1392	215486450	chaB;predicted cation regulator	0.01	-2.36	0.26
1395	215486453	ychO;predicted invasin	0.00	-2.64	1.19
1396	215486454	narL;DNA-binding response regulator in two-component regulatory system with NarX (or	0.01	-2.16	0.23
1402	215486460	narI;nitrate reductase 1, gamma (cytochrome b(NR)) subunit	0.00	-2.52	0.46
1404	215486462	-;predicted dehydrogenase	0.00	-2.79	0.54
1405	215486463	-;hypothetical protein	0.00	-2.45	0.35
1409	215486467	-;hypothetical protein	0.00	-3.68	0.38
1410	215486468	purU;formyltetrahydrofolate	0.00	2.34	0.34
1411	215486469	ychJ;hypothetical protein	0.00	5.95	0.72
1412	215486470	rssA;hypothetical protein	0.00	2.49	0.26
1416	215486474	tdk;thymidine kinase/deoxyuridine kinase	0.00	4.75	0.67
1417	215486475	adhE;fused acetaldehyde-CoA dehydrogenase/iron-dependent alcohol dehydrogenase/pyruvate-	0.00	-5.20	0.40
1420	215486478	oppB;oligopeptide transporter subunit OppB	0.00	-2.94	0.47
1421	215486479	oppC;oligopeptide transporter subunit OppC	0.01	-2.08	0.28
1426	215486484	yciY;hypothetical protein	0.00	13.91	1.16
1428	215486486	yciI;predicted enzyme	0.00	2.90	0.71
1429	215486487	tonB;membrane spanning protein TonB in TonB-ExbB-	0.00	5.55	0.16
1430	215486488	yciA;predicted hydrolase	0.00	2.59	0.38
1431	215486489	yciB;predicted inner membrane	0.00	2.58	0.11
1448	215486506	-;predicted antitermination	0.01	-2.36	0.74

1459	215486518	−;predicted holin protein	0.02	−2.57	0.51
1461	215486520	−;predicted endolysin	0.00	−3.96	0.39
1463	215486522	−;hypothetical protein	0.00	−3.24	0.47
1464	215486523	−;predicted endopeptidase	0.00	−2.22	0.64
1470	215486529	−;predicted terminase large	0.01	−2.00	0.15
1471	215486530	−;predicted head protein/prohead protease	0.00	−2.06	0.45
1473	215486532	−;predicted portal protein	0.00	−4.21	0.37
1476	215486535	−;predicted minor tail protein	0.03	−2.03	1.74
1480	215486539	−;predicted minor tail protein	0.00	−2.74	0.61
1487	215486546	−;predicted outer membrane precursor Lom	0.00	−2.41	1.76
1490	215486549	−;hypothetical protein	0.00	2.45	0.79
1492	215486551	−;T3SS secreted effector NleH	0.00	−3.44	0.25
1493	215486552	−;T3SS secreted effector NleF	0.00	−8.05	0.30
1495	215486556	trpB;tryptophan synthase, beta	0.00	−2.11	0.75
1496	215486557	trpC;fused indole−3−glycerolphosphate synthetase/N−(5−phosphoribosyl) anthranilate	0.00	−2.43	0.09
1501	215486562	yciO;hypothetical protein	0.00	3.32	0.38
1503	215486564	rluB;23S rRNA pseudouridy late synthase	0.00	2.33	0.21
1505	215486566	yciK;predicted oxoacyl−(acyl carrier protein) reductase, EmrKY−TolC system	0.00	4.19	0.14
1507	215486568	yciN;hypothetical protein	0.00	3.06	0.45
1508	215486569	topA;DNA topoisomerase I, omega subunit	0.00	−2.70	0.23
1509	215486570	cysB;DNA−binding transcriptional dual regulator, O−acetyl−L−	0.00	−2.43	0.22
1511	215486572	yciX;hypothetical protein	0.00	2.23	0.98
1512	215486573	acnA;aconitate hydratase 1	0.00	−7.90	0.60
1513	215486574	ribA;GTP cyclohydrolase II	0.00	3.09	0.12
1515	215486576	yciS;conserved inner membrane protein	0.02	2.02	0.55
1518	215486579	yciH;hypothetical protein	0.00	2.56	0.09
1520	215486581	yciT;predicted DNA−binding transcriptional regulator	0.00	−2.27	0.18
1522	215486583	gmr;modulator of Rnase II	0.00	−2.75	0.21
1531	215486594	sapF;predicted ATP−binding protein SapF of peptide transport	0.00	−2.42	0.49
1532	215486595	sapD;predicted ATP−binding protein SapD of peptide	0.00	−3.25	0.30
1533	215486596	sapC;predicted peptide transport permease protein SapC; membrane component of ABC	0.00	−2.33	0.20
1534	215486597	sapB;predicted peptide transport permease protein SapB; membrane component of ABC	0.00	−2.63	0.11
1535	215486598	sapA;predicted peptide transport periplasmic protein SapA; periplasmic−binding component	0.00	−2.87	0.42
1542	215486605	pspE;thiosulfate: cyanide sulfurtransferase (rhodanese)	0.00	2.97	0.16
1550	215486614	ycjU;predicted beta−phosphoglucomutase	0.00	−2.51	0.40
1561	215486625	ycjY;predicted hydrolase	0.00	−2.29	0.74
1566	215486630	uspE;stress−induced protein	0.00	−4.01	0.40
1570	215486634	−;predicted multidrug resistance efflux pump, HlyD family	0.00	−2.51	0.51
1575	215486639	uspF;stress−induced protein, ATP−binding protein	0.00	3.48	0.58
1576	215486640	ompN;outer membrane pore protein N, non−specific	0.01	−2.45	0.26
1577	215486641	ydbK;fused predicted pyruvate−flavodoxin oxidoreductase: conserved protein/conserved protein/FeS binding protein	0.00	−2.29	0.94
1578	215486642	ydbJ;hypothetical protein	0.00	2.40	0.27
1579	215486643	hslJ;heat−inducible protein HslJ	0.00	3.17	0.29

1580	215486644	ldhA;fermentative D-lactate dehydrogenase, NAD-dependent	0.00	-3.28	0.42
1586	215486651	hrpA;ATP-dependent helicase	0.00	-8.05	0.36
1594	215486661	ydcI;predicted DNA-binding transcriptional regulator	0.00	4.71	0.08
1606	215486673	yncJ;hypothetical protein	0.00	5.93	0.35
1610	215486677	ydcU;predicted spermidine/putrescine	0.01	-2.25	0.29
1611	215486678	ydcV;predicted spermidine/putrescine transporter subunit, membrane	0.01	-2.05	0.32
1612	215486679	ydcW;medium chain aldehyde dehydrogenase	0.00	-3.22	0.54
1613	215486680	yncL;hypothetical protein	0.00	2.50	1.40
1614	215486681	ydcX;predicted inner membrane	0.00	3.89	0.96
1615	215486682	ydcY;hypothetical protein	0.00	5.43	0.83
1619	215486686	yncE;hypothetical protein	0.00	5.87	0.25
1620	215486687	ansP;L-asparagine transporter	0.00	-2.53	0.36
1627	215486694	narV;nitrate reductase 2 (NRZ), gamma subunit	0.00	-2.98	0.24
1628	215486695	narW;nitrate reductase 2 (NRZ), delta subunit (assembly subunit)	0.00	-3.27	0.13
1629	215486696	narY;nitrate reductase 2 (NRZ), beta subunit	0.00	-4.40	0.19
1631	215486698	narU;nitrate/nitrite transporter	0.00	-2.06	0.03
1632	215486699	yddG;predicted methyl viologen efflux pump	0.00	2.18	0.12
1637	215486704	adhP;alcohol dehydrogenase, 1-propanol preferring	0.00	-4.00	0.13
1638	215486705	maeA;malate dehydrogenase, (decarboxylating, NAD-requiring) (malic enzyme)	0.01	-2.15	0.67
1640	215486707	bdm;biofilm-dependent modulation protein	0.00	14.98	0.69
1641	215486708	osmC;osmotically inducible, stress-inducible membrane	0.00	-2.82	0.14
1642	215486709	dos;cAMP phosphodiesterase, heme-regulated	0.00	-2.88	0.53
1643	215486710	yddW;predicted liprotein	0.01	2.13	0.51
1644	215486711	gadC;predicted glutamate:gamma-aminobutyric acid	0.00	-8.76	0.59
1645	215486712	gadB;glutamate decarboxylase B, PLP-dependent	0.00	-13.07	0.95
1648	215486715	yddA;fused predicted multidrug transporter subunits of ABC superfamily: membrane component/ATP-binding	0.00	-3.60	0.47
1656	215486723	ydeS;predicted fimbrial protein-like protein	0.00	-3.25	0.24
1658	215486725	-;predicted fimbrial chaperone protein precursor	0.03	-2.09	0.50
1659	215486726	-;predicted type 1 fimbrial protein precursor	0.00	-9.93	0.50
1665	215486732	tam;trans-aconitate	0.01	2.10	0.43
1670	215486737	yneH;predicted glutaminase	0.01	2.07	0.10
1671	215486738	yneI;predicted aldehyde dehydrogenase	0.00	-2.40	0.15
1674	215486741	marC;predicted transporter	0.00	3.19	0.23
1676	215486743	marA;DNA-binding transcriptional dual activator of multiple antibiotic resistance	0.00	4.26	0.08
1677	215486744	marB;hypothetical protein	0.00	4.88	0.25
1680	215486747	yneM;hypothetical protein	0.00	-5.86	0.91
1686	215486753	ydfI;predicted mannonate dehydrogenase	0.03	-2.17	0.44
1691	215486758	ynfB;hypothetical protein	0.00	3.98	0.86
1692	215486759	speG;spermidine N1-	0.00	4.83	0.59
1695	215486762	ynfE;oxidoreductase subunit	0.00	-2.88	0.80
1697	215486764	ynfG;oxidoreductase, Fe-S	0.00	-4.97	0.81
1708	215486775	mdtI;multidrug efflux system protein MdtI	0.00	7.04	0.70

1710	215486777	tqsA;predicted inner membrane	0.00	2.28	0.37
1713	215486780	ydgH;hypothetical protein	0.00	3.73	0.25
1714	215486781	ydgI;predicted arginine/ornithine antiporter	0.03	-2.17	0.25
1715	215486782	folM;dihydrofolate reductase	0.00	-2.31	0.19
1718	215486785	rstB;sensory histidine kinase in two-component regulatory	0.00	-2.24	0.40
1719	215486786	tus;inhibitor of replication at Ter, DNA-binding protein	0.00	-3.58	0.72
1721	215486788	fumA;fumarate hydratase (fumarase A), aerobic ClassI	0.00	2.79	0.28
1723	215486790	ydgA;hypothetical protein	0.00	-3.37	0.25
1727	215486794	hdhA;7-alpha-hydroxysteroid dehydrogenase, NAD-dependent	0.02	-2.07	0.66
1729	215486796	malX;fused maltose and glucose-specific PTS enzymes: IIB component, IIC component	0.00	-2.77	0.55
1731	215486798	add;adenosine deaminase	0.00	-2.44	0.35
1732	215486800	ydgJ;predicted oxidoreductase	0.00	2.45	0.26
1733	215486801	blr;beta-lactam resistance membrane protein Blr	0.00	5.16	0.79
1736	215486804	rsxA;predicted inner membrane	0.00	5.35	0.37
1737	215486805	rsxB;predicted iron-sulfur protein	0.00	5.11	0.18
1745	215486813	pdxY;pyridoxal kinase 2/pyridoxine kinase	0.00	-3.34	0.37
1748	215486816	ydhA;predicted lipoprotein	0.00	5.40	0.52
1751	215486819	slyA;DNA-binding transcriptional activator	0.00	4.29	0.22
1755	215486823	sodC;superoxide dismutase, Cu,	0.01	-2.15	0.47
1756	215486824	ydhF;predicted oxidoreductase	0.00	-2.72	0.87
1758	215486826	ydhM;predicted DNA-binding transcriptional regulator	0.00	3.02	0.40
1761	215486829	rnt;ribonuclease T (RNase T)	0.01	3.40	0.42
1763	215486831	ydhO;predicted lipoprotein	0.00	10.16	0.33
1769	215486837	ydhC;predicted transporter	0.00	-2.56	0.32
1770	215486838	cfa;cyclopropane fatty acyl phospholipid synthase (unsaturated-phospholipid	0.02	-2.12	0.33
1771	215486839	ribC;riboflavin synthase, alpha	0.00	3.41	0.68
1774	215486842	ydhR;hypothetical protein	0.02	-2.04	0.91
1775	215486843	ydhS;conserved protein with FAD/NAD(P)-binding domain	0.00	-14.69	0.72
1782	215486850	ydhZ;hypothetical protein	0.00	2.92	1.30
1783	215486851	pykF;pyruvate kinase I	0.00	-2.49	0.78
1786	215486854	sufE;sulfur acceptor protein	0.00	-5.00	0.12
1787	215486855	sufS;selenocysteine lyase, PLP-dependent	0.00	-5.94	0.14
1788	215486856	sufD;component of SufBCD	0.00	-5.30	0.54
1789	215486857	sufC;component of SufBCD complex, ATP-binding component of ABC superfamily	0.00	-2.90	0.51
1790	215486858	sufB;component of SufBCD	0.00	-3.96	0.61
1795	215486863	ydiK;predicted inner membrane	0.00	-2.84	0.39
1801	215486869	ydiF;fused predicted acetyl-CoA: acetoacetyl-CoA transferase: alpha subunit/beta subunit	0.00	-2.36	0.86
1810	215486878	ydiA;hypothetical protein	0.00	3.03	0.46
1812	215486880	ydiE;hypothetical protein	0.00	6.95	0.95
1814	215486882	ydiV;hypothetical protein	0.00	2.61	0.99
1815	215486883	nlpC;predicted lipoprotein	0.00	3.69	0.42
1817	215486885	btuE;predicted glutathione	0.00	3.72	0.27
1820	215486888	-;predicted repressor protein	0.00	2.23	0.83
1821	215486889	-;hypothetical protein	0.01	-2.02	0.33
1826	215486894	-;hypothetical protein	0.01	-2.18	0.57
1831	215486899	-;predicted ATPase, AAA-	0.00	-6.50	0.46
1832	215486900	-;predicted proteases	0.00	-4.31	0.33
1836	215486904	-;predicted major capsid protein	0.00	-3.45	0.73
1837	215486905	-;predicted terminase, endonuclease subunit	0.00	-2.33	0.15
1840	215486908	-;predicted holin protein	0.00	-2.18	0.35
1841	215486909	-;predicted endolysin	0.00	-2.69	0.37

1842	215486910	-;hypothetical protein	0.03	-2.07	0.71
1843	215486911	-;predicted tail protein	0.00	-3.18	0.40
1844	215486912	-;predicted tail protein	0.01	-2.84	0.36
1845	215486913	-;predicted baseplate assembly	0.01	-2.15	0.82
1848	215486916	-;predicted tail protein	0.00	-2.50	0.37
1856	215486924	-;predicted tail protein	0.01	-2.04	0.16
1857	215486925	-;predicted tail protein	0.02	-2.01	0.13
1858	215486926	-;predicted tail tube protein	0.00	-4.70	0.22
1859	215486927	-;predicted tail sheath protein	0.00	-4.01	0.31
1860	215486928	-;predicted late gene regulator	0.00	-2.53	0.09
1862	215486930	-;predicted regulatory protein	0.00	3.07	0.29
1867	215486936	rpmI;50S ribosomal subunit	0.01	-2.32	0.67
1870	215486939	yniD;hypothetical protein	0.01	2.01	0.67
1873	215486942	ydiZ;hypothetical protein	0.00	-3.04	0.44
1874	215486943	yniA;predicted phosphotransferase/kinase	0.03	-2.02	0.48
1880	215486949	katE;hydroperoxidase HPII (III) (catalase)	0.00	-5.44	0.24
1883	215486952	chbR;DNA-binding transcriptional dual regulator	0.00	-2.31	0.09
1886	215486955	chbB;N,N'-diacetylchitobiose-specific enzyme IIB component	0.00	2.95	0.45
1890	215486959	vse;hypothetical protein	0.00	-2.14	0.94
1891	215486960	spy;envelope stress induced periplasmic protein	0.00	-3.81	0.39
1892	215486961	astE;succinylglutamate	0.00	-3.30	0.09
1893	215486962	astB;succinylarginine dihydrolase	0.00	-6.77	0.57
1894	215486963	astD;succinylglutamic semialdehyde dehydrogenase	0.00	-2.88	0.42
1896	215486965	astC;succinylornithine transaminase AstC, PLP-	0.00	2.97	0.84
1897	215486966	xthA;exonuclease III	0.01	2.01	0.20
1900	215486969	ydjZ;conserved inner membrane protein	0.00	-2.71	0.27
1908	215486977	ynjH;hypothetical protein	0.00	2.34	0.64
1916	215486985	ydjE;predicted transporter	0.01	-2.06	0.12
1917	215486986	ydjF;predicted DNA-binding transcriptional regulator	0.00	-2.94	0.22
1918	215486987	ydjG;predicted oxidoreductase	0.01	-2.17	0.21
1919	215486988	ydjH;predicted kinase	0.00	-3.15	0.21
1924	215486993	yeaC;hypothetical protein	0.00	3.76	0.26
1929	215486998	mipA;scaffolding protein for murein synthesizing machinery	0.00	2.98	0.23
1930	215486999	yeaG;conserved protein with nucleoside triphosphate hydrolase domain	0.00	-6.07	0.40
1931	215487000	yeaH;hypothetical protein	0.00	-11.03	0.56
1933	215487002	yeaJ;predicted diguanylate	0.01	-2.08	0.13
1934	215487003	yeaK;hypothetical protein	0.00	2.41	0.61
1939	215487008	yeaO;hypothetical protein	0.00	3.15	0.65
1941	215487010	yeaP;predicted diguanylate	0.00	2.29	0.44
1943	215487012	yoaG;hypothetical protein	0.01	2.10	0.18
1944	215487013	yeaR;hypothetical protein	0.00	-2.24	1.10
1945	215487014	leuE;neutral amino-acid efflux	0.00	2.45	0.52
1949	215487018	yeaZ;predicted peptidase	0.00	2.68	0.94
1951	215487020	yoaB;hypothetical protein	0.00	7.41	0.69
1953	215487022	yoaH;hypothetical protein	0.00	5.44	0.59
1957	215487026	yoaD;predicted	0.00	-2.92	0.31
1958	215487027	yoaE;fused predicted membrane protein/conserved protein	0.00	2.88	0.78
1959	215487028	-;hypothetical protein	0.00	4.15	0.88
1968	215487037	yebO;hypothetical protein	0.00	2.35	0.89
1969	215487038	mgrB;hypothetical protein	0.01	2.09	1.03
1970	215487039	yobH;hypothetical protein	0.00	9.34	0.93
1973	215487042	htpX;predicted endopeptidase	0.00	3.08	0.39
1975	215487044	proQ;predicted structural transport element	0.00	6.03	0.30
1976	215487045	yebR;hypothetical protein	0.00	3.86	0.30
1978	215487047	yebT;hypothetical protein	0.00	-2.24	0.67
1980	215487049	yebV;hypothetical protein	0.00	2.80	0.70
1988	215487057	exoX;DNA exonuclease X	0.01	-2.00	0.17

1990	215487059	yebE;hypothetical protein	0.00	2.79	0.29
1997	215487066	yebK;predicted DNA-binding transcriptional regulator	0.00	4.05	0.38
1999	215487068	lpxM;myristoyl-acyl carrier protein (ACP)-dependent	0.00	5.60	0.46
2001	215487070	znuA;zinc transporter subunit ZnuA: periplasmic-binding component of ABC superfamily	0.01	2.31	0.22
2005	215487074	ruvA;RuvA component of RuvABC resolvasome, regulatory	0.00	2.63	0.39
2008	215487077	yebC;hypothetical protein	0.00	-2.65	0.35
2009	215487078	nudB;dATP	0.00	-2.11	0.31
2012	215487081	yecE;hypothetical protein	0.01	-2.23	0.56
2013	215487082	yecN;predicted inner membrane	0.00	3.25	0.85
2014	215487083	cmoA;predicted	0.00	7.66	0.15
2015	215487084	cmoB;predicted S-adenosyl-L-methionine-dependent methyltransferase	0.00	4.91	0.34
2019	215487088	yecM;predicted metal-binding	0.00	-3.78	0.39
2036	215487105	otsA;trehalose-6-phosphate	0.00	-3.00	0.69
2039	215487109	araF;L-arabinose transporter	0.01	2.08	0.55
2040	215487110	ftnB;predicted ferritin-like protein	0.00	7.88	0.53
2044	215487114	yecH;hypothetical protein	0.01	-2.21	1.52
2048	215487118	uvrC;excinuclease UvrABC, endonuclease subunit	0.00	-2.30	0.30
2049	215487119	uvrY;DNA-binding response regulator UvrY in two-component regulatory system with BarA	0.01	2.10	0.61
2050	215487120	yecF;hypothetical protein	0.00	2.10	1.02
2051	215487121	sdiA;DNA-binding transcriptional activator	0.00	-2.70	0.19
2055	215487125	fliY;cystine transporter subunit	0.00	2.30	0.33
2060	215487130	fliS;flagellar protein potentiates polymerization	0.00	-2.28	0.03
2062	215487132	amyA;cytoplasmic alpha-	0.00	-3.58	0.25
2064	215487134	yedF;hypothetical protein	0.00	3.68	0.89
2073	215487143	fliK;flagellar hook-length control protein FliK	0.03	-2.38	0.43
2075	215487145	fliM;flagellar motor switching and energizing component	0.00	-3.19	0.50
2077	215487147	fliO;flagellar biosynthesis protein	0.00	-2.30	0.61
2078	215487148	fliP;flagellar biosynthesis protein	0.00	-3.01	0.34
2081	215487151	rcsA;DNA-binding transcriptional activator, co-regulator with RcsB	0.00	2.32	0.17
2084	215487154	yedP;hypothetical protein	0.01	-2.21	0.49
2085	215487155	yodC;hypothetical protein	0.00	-2.60	0.97
2087	215487157	yedA;predicted inner membrane	0.00	-2.42	0.37
2088	215487158	vsr;DNA mismatch endonuclease Vsr of very short patch repair	0.00	-2.41	0.44
2092	215487162	ysdS;hypothetical protein	0.05	2.01	0.29
2094	215487164	yedV;predicted sensory kinase in two-component regulatory system with YedW	0.01	-2.18	0.24
2095	215487165	yedW;predicted DNA-binding response regulator in two-component system with YedV	0.00	-2.54	0.15
2098	215487168	yedZ;conserved inner membrane protein	0.01	-2.15	0.27
2100	215487170	mtfA;hypothetical protein	0.00	3.11	0.14
2103	215487173	amn;AMP nucleosidase	0.00	-2.67	1.00
2104	215487174	yeeN;hypothetical protein	0.00	5.44	0.37
2108	215487178	-;H-NS-like DNA-binding	0.00	-3.46	0.46
2112	215487182	-;predicted DNA-binding protein	0.00	-2.84	0.36
2119	215487189	-;hypothetical protein	0.00	-2.50	0.47
2127	215487197	-;predicted P4-family integrase	0.00	-3.10	0.45
2128	215487198	-;hypothetical protein	0.00	-3.82	0.35
2143	215487213	pduC;propanediol dehydratase, large subunit, AdoCbl-	0.00	-3.08	0.16
2147	215487217	pduH;propanediol dehydratase reactivation protein PduH	0.00	-2.62	0.25

2148	215487218	pduJ;propanediol utilization protein PduJ	0.00	-2.73	0.23
2149	215487219	pduK;propanediol utilization protein PduK	0.00	-4.78	0.53
2156	215487226	pduS;predicted propanediol utilization protein	0.00	-2.42	0.39
2157	215487227	pduT;propanediol utilization protein PduT	0.00	-2.14	0.06
2159	215487229	pduV;predicted propanediol utilization protein	0.04	-2.09	0.72
2160	215487230	yeeX;hypothetical protein	0.01	2.09	0.57
2162	215487232	sbmC;DNA gyrase inhibitor	0.02	-2.08	0.44
2166	215487236	yeeY;predicted DNA-binding transcriptional regulator	0.00	-2.45	0.36
2170	215487240	hisL;his operon leader peptide	0.00	-3.42	0.86
2175	215487245	hisH;imidazole glycerol phosphate synthase, glutamine amidotransferase subunit with	0.03	-2.33	0.86
2177	215487247	hisF;imidazole glycerol phosphate synthase, catalytic	0.00	-2.10	0.06
2178	215487248	hisI;fused phosphoribosyl-AMP cyclohydrolase/phosphoribosyl-ATP pyrophosphatase	0.00	-2.08	0.83
2179	215487249	wzz;regulator of length of O-antigen component of lipopolysaccharide chains	0.00	6.06	0.34
2183	215487253	wbiP;predicted glycosyl	0.00	-2.39	0.63
2189	215487259	-;GDP-mannose mannosyl	0.00	-3.90	1.07
2194	215487265	gne;putative UDP-galactose 4-epimerase	0.00	5.77	1.01
2205	215487276	gmd;GDP-D-mannose dehydratase, NAD(P)-binding	0.00	-2.59	0.42
2206	215487277	wcaF;predicted acyl transferase	0.05	-2.01	1.26
2209	215487280	wcaC;predicted glycosyl transferase WcaC	0.00	-2.43	0.47
2214	215487285	wza;lipoprotein required for capsular polysaccharide translocation through the outer	0.00	-2.77	0.32
2217	215487288	dcd;2'-deoxycytidine 5'-triphosphate deaminase	0.00	2.30	0.18
2218	215487289	udk;uridine/cytidine kinase	0.00	3.67	0.40
2225	215487298	mdtB;multidrug efflux system,	0.00	-2.81	0.70
2226	215487299	mdtC;multidrug efflux system,	0.00	-2.06	0.47
2238	215487311	altR;D-arabitol repressor	0.00	2.39	0.35
2242	215487315	gatB;galactitol-specific enzyme IIB component of PTS	0.00	-2.37	0.11
2244	215487317	gatZ;D-tagatose 1,6-bisphosphate aldolase 2, subunit	0.00	-2.17	0.33
2246	215487319	fbaB;fructose-bisphosphate aldolase class I	0.00	-2.65	0.50
2250	215487323	yegW;predicted DNA-binding transcriptional regulator	0.00	4.00	0.44
2252	215487325	thiD;bifunctional hydroxy-methylpyrimidine kinase/hydroxy-	0.00	-2.66	0.43
2256	215487329	yohN;hypothetical protein	0.00	3.21	0.65
2260	215487334	yehE;hypothetical protein	0.00	3.03	0.66
2269	215487343	yehQ;hypothetical protein	0.00	-2.88	0.27
2270	215487344	yehR;hypothetical protein	0.00	3.30	0.06
2271	215487345	yehS;hypothetical protein	0.00	3.01	0.57
2274	215487348	mlrA;DNA-binding transcriptional regulator	0.00	2.76	0.21
2275	215487349	yohO;hypothetical protein	0.00	3.72	0.45
2276	215487350	yehW;predicted transporter subunit: membrane component of ABC superfamily	0.01	-2.01	0.46
2278	215487352	yehY;predicted transporter subunit: membrane component of ABC superfamily	0.01	-2.25	0.63

2279	215487353	osmF;predicted transporter subunit: periplasmic-binding component of ABC superfamily	0.00	2.30	0.06
2283	215487357	yohC;predicted inner membrane	0.00	-5.13	0.37
2288	215487362	yohJ;conserved inner membrane protein	0.00	-2.36	0.46
2297	215487371	mglB;methyl-galactoside transporter subunit	0.00	3.73	0.03
2298	215487372	galS;DNA-binding transcriptional repressor	0.00	5.89	0.44
2299	215487373	yeiB;conserved inner membrane protein	0.00	-3.90	0.19
2300	215487374	folE;GTP cyclohydrolase I	0.00	3.84	0.26
2301	215487375	yeiG;predicted esterase	0.00	-2.72	0.42
2314	215487388	fruA;fused fructose-specific PTS enzymes: IIB and IIC	0.01	-2.29	0.22
2319	215487393	yeiP;predicted elongation factor	0.00	3.79	0.20
2323	215487397	spr;predicted peptidase, outer membrane lipoprotein	0.00	10.84	1.34
2326	215487400	yejB;predicted oligopeptide transporter subunit	0.00	-5.03	0.29
2331	215487405	rsuA;16S rRNA pseudouridylate 516 synthase	0.00	3.58	0.28
2332	215487406	yejH;predicted ATP-dependet	0.00	-3.01	0.41
2333	215487407	rplY;50S ribosomal subunit	0.00	5.65	0.64
2334	215487408	yejK;nucleotide associated	0.00	3.12	0.32
2335	215487409	yejL;hypothetical protein	0.00	2.32	1.28
2337	215487411	narP;DNA-binding response regulator in two-component regulatory system with NarQ or	0.00	2.57	0.39
2339	215487413	ccmG;periplasmic thioredoxin CcmG of cytochrome c-type	0.01	-2.21	0.70
2340	215487414	ccmF;heme lyase, CcmF subunit	0.00	-2.67	0.35
2346	215487420	napC;nitrate reductase, cytochrome c-type, periplasmic	0.00	7.24	0.53
2351	215487425	napD;periplasmic nitrate reductase assembly protein	0.02	-2.11	0.31
2353	215487427	yojO;hypothetical protein	0.01	-2.05	1.03
2354	215487428	eco;ecotin, a serine protease	0.01	2.07	0.29
2357	215487431	alkB;oxidative demethylase of N1-methyladenine or N3-methylcytosine DNA lesions	0.00	-3.07	0.61
2372	215487446	yfaS;hypothetical protein	0.00	-3.07	0.70
2377	215487451	nrdA;ribonucleoside diphosphate reductase 1, alpha subunit	0.00	-2.32	0.15
2380	215487454	inaA;hypothetical protein	0.00	2.82	0.08
2382	215487456	glpT;sn-glycerol-3-phosphate transporter	0.00	-3.26	0.69
2386	215487460	yfaD;hypothetical protein	0.00	-4.66	0.86
2387	215487461	ypaA;hypothetical protein	0.00	3.94	0.29
2389	215487463	yfaV;predicted transporter	0.00	-2.95	0.29
2397	215487471	arnC;undecaprenyl phosphate-L-Ara 4FN transferase	0.00	-2.76	0.54
2398	215487472	arnA;fused UDP-L-Ara4N formyltransferase/UDP-GlcA C-4'-decarboxylase	0.00	2.25	0.30
2399	215487473	yfbH;hypothetical protein	0.00	-3.79	0.86
2403	215487477	pmrD;polymyxin resistance	0.01	2.16	1.07
2405	215487479	menC;o-succinylbenzoyl-CoA synthase MenC	0.00	-3.43	0.77
2411	215487485	elaA;predicted acyltransferase with acyl-CoA N-acyltransferase	0.01	-2.13	0.41
2417	215487491	nuoL;NADH: ubiquinone oxidoreductase, membrane	0.00	-3.97	0.55
2420	215487494	nuoI;NADH: ubiquinone oxidoreductase, chain I	0.00	-3.12	0.86
2421	215487495	nuoH;NADH: ubiquinone oxidoreductase, membrane	0.00	-2.91	0.05
2422	215487496	nuoG;NADH: ubiquinone oxidoreductase, chain G	0.01	-2.18	0.95

2423	215487497	nuoF;NADH: ubiquinone oxidoreductase, chain F	0.00	-4.39	0.64
2425	215487499	nuoC;NADH: ubiquinone oxidoreductase, chain C, D	0.00	-2.34	0.12
2426	215487500	nuoB;NADH: ubiquinone oxidoreductase, chain B	0.00	2.34	0.40
2428	215487502	lrhA;DNA-binding transcriptional repressor of flagellar, motility and chemotaxis genes	0.01	2.04	0.36
2438	215487512	yfcD;predicted NUDIX hydrolase	0.00	6.47	0.33
2439	215487513	yfcE;predicted phosphatase	0.01	2.04	0.17
2442	215487516	folX;D-erythro-7,8-dihydroneopterin triphosphate 2'-epimerase and	0.00	2.72	0.43
2443	215487517	yfcH;conserved protein with NAD(P)-binding Rossmann-fold	0.01	2.05	0.35
2450	215487524	ubiX;3-octaprenyl-4-hydroxybenzoate carboxy-lyase	0.00	5.20	0.24
2451	215487525	purF;amidophosphoribosyltransfe	0.00	-3.03	0.41
2455	215487529	accD;acetyl-CoA carboxylase, beta (carboxyltranferase) subunit	0.00	8.02	0.10
2458	215487532	usg;predicted semialdehyde dehydrogenase	0.00	2.38	0.14
2460	215487534	flk;predicted flagella assembly	0.00	2.28	0.24
2463	215487537	mnmC;fused 5-methylaminomethyl-2-thiouridine-forming enzyme methyltransferase/FAD-	0.00	2.87	0.72
2465	215487539	yfcM;hypothetical protein	0.00	2.34	0.40
2479	215487553	fadJ;fused enoyl-CoA hydratase and epimerase andisomerase/3-hydroxyacyl-CoA dehydrogenase	0.00	-3.11	0.35
2481	215487555	yfcZ;hypothetical protein	0.00	2.92	0.51
2482	215487556	fadL;long-chain fatty acid outer membrane transporter	0.00	2.86	0.88
2483	215487557	vacJ;predicted lipoprotein	0.00	2.19	0.44
2484	215487558	yfdC;predicted inner membrane	0.00	-2.46	0.20
2485	215487559	-;predicted integrase	0.02	-2.07	0.37
2502	215487576	-;hypothetical protein	0.00	-2.35	0.15
2504	215487578	-;hypothetical protein	0.00	-2.04	0.11
2505	215487579	-;hypothetical protein	0.00	-2.75	0.61
2506	215487580	-;hypothetical protein	0.00	-8.46	0.41
2508	215487582	-;hypothetical protein	0.00	-2.64	0.13
2521	215487595	-;hypothetical protein	0.00	2.51	0.25
2526	215487600	-;predicted DNA methylase	0.01	2.10	0.43
2527	215487601	-;NinB	0.01	-2.17	0.34
2538	215487612	-;hypothetical protein	0.01	-2.01	0.19
2541	215487615	-;predicted regulatory protein	0.00	-2.28	0.84
2543	215487617	-;hypothetical protein	0.00	3.64	0.14
2544	215487618	-;hypothetical protein	0.00	4.29	0.44
2545	215487619	-;hypothetical protein	0.00	8.33	0.35
2546	215487620	-;predicted single stranded DNA-binding protein	0.00	4.64	0.06
2547	215487621	-;predicted anti-RecBCD protein	0.00	7.54	0.34
2548	215487622	-;hypothetical protein	0.00	9.43	0.38
2550	215487624	-;hypothetical protein	0.00	5.56	0.08
2551	215487625	-;hypothetical protein	0.00	3.38	0.37
2556	215487630	-;sucrose operon repressor	0.00	2.15	0.73
2558	215487632	emrK;EmrKY-TolC multidrug resistance efflux pump protein K, membrane fusion protein	0.00	-2.07	0.49
2559	215487633	evgA;DNA-binding response regulator in two-component regulatory system with EvgS	0.00	3.09	0.75
2561	215487635	yfdE;predicted CoA-transferase, NAD(P)-binding	0.00	-2.38	0.20
2562	215487636	yfdV;predicted transporter	0.00	-2.93	0.27
2564	215487638	frc;formyl-CoA transferase, NAD(P)-binding	0.00	-2.63	1.00
2565	215487639	yfdX;hypothetical protein	0.00	-6.97	0.68

2568	215487642	lpxP;palmitoleoyl-acyl carrier protein (ACP)-dependent	0.00	-2.48	0.06
2572	215487646	ypdC;predicted DNA-binding	0.00	-2.25	0.26
2578	215487652	yfeO;predicted ion channel	0.00	3.93	0.16
2579	215487653	ypeC;hypothetical protein	0.00	4.82	0.12
2580	215487654	mntH;manganese/divalent cation transporter	0.00	-3.86	0.35
2581	215487655	nupC;nucleoside (except guanosine) transporter	0.00	8.60	0.06
2583	215487657	yfeC;predicted DNA-binding transcriptional regulator	0.00	2.50	0.72
2584	215487658	yfeD;predicted DNA-binding transcriptional regulator	0.00	4.40	0.24
2586	215487660	xapB;xanthosine transporter	0.00	-2.70	0.61
2588	215487662	yfeN;conserved outer membrane protein	0.00	5.20	0.88
2591	215487665	ypeB;hypothetical protein	0.00	-2.42	0.22
2592	215487666	ligA;DNA ligase, NAD(+)-	0.00	-2.61	0.14
2593	215487667	zipA;cell division protein involved in Z ring assembly	0.00	2.60	0.09
2594	215487668	cysZ;predicted inner membrane	0.00	4.93	0.25
2595	215487669	cysK;cysteine synthase A, O-acetylserine sulfhydrolase A	0.00	7.87	0.47
2597	215487671	ptsI;PEP-protein phosphotransferase of PTS	0.00	-4.80	0.60
2606	215487681	ucpA;predicted oxidoredutase, sulfate metabolism protein	0.00	2.28	0.32
2610	215487685	yfeY;hypothetical protein	0.00	2.20	0.21
2626	215487701	-;hypothetical protein	0.00	-2.91	0.46
2627	215487702	-;hypothetical protein	0.01	-2.04	0.32
2631	215487706	-;hypothetical protein	0.00	-2.31	0.21
2640	215487715	-;hypothetical protein	0.01	-2.29	0.57
2644	215487719	-;hypothetical protein	0.00	-4.06	1.01
2647	215487722	-;hypothetical protein	0.00	-2.39	0.86
2656	215487731	-;hypothetical protein	0.01	-2.13	0.20
2667	215487742	-;predicted tail protein	0.00	-4.08	0.24
2690	215487766	tktB;transketolase 2, thiamin-	0.00	-7.35	0.34
2694	215487770	narQ;sensory histidine kinase in two-component regulatory system with NarP (NarL)	0.01	-2.08	0.20
2705	215487781	nlpB;lipoprotein	0.00	4.73	0.55
2706	215487782	dapA;dihydrodipicolinate	0.00	2.69	0.18
2710	215487786	yfgC;predicted peptidase	0.00	-3.46	0.46
2712	215487788	hda;ATPase regulatory factor involved in DnaA inactivation	0.00	4.89	0.05
2714	215487790	upp;uracil	0.00	3.87	0.49
2715	215487791	purM;phosphoribosylaminoimidazole synthetase	0.00	2.39	0.33
2716	215487792	purN;phosphoribosylglycinamide formyltransferase 1	0.00	3.51	0.33
2717	215487793	ppk;polyphosphate kinase, component of RNA	0.01	-2.23	0.37
2719	215487795	yfgF;predicted inner membrane	0.02	-2.03	0.53
2731	215487807	-;hypothetical protein	0.00	3.33	1.31
2739	215487815	-;hypothetical protein	0.01	-2.20	0.51
2740	215487816	-;hypothetical protein	0.00	-3.12	0.44
2742	215487818	-;hypothetical protein	0.00	-2.29	0.18
2743	215487819	-;predicted endoprotease	0.04	-2.03	0.85
2745	215487821	-;hypothetical protein	0.00	-4.23	0.59
2747	215487823	-;hypothetical protein	0.01	-2.32	0.64
2759	215487835	-;predicted primosomal protein	0.01	2.03	0.38
2760	215487836	-;hypothetical protein	0.00	2.56	0.57
2773	215487849	guaA;GMP synthetase (glutamine aminotransferase)	0.00	-4.20	1.01
2774	215487850	guaB;IMP dehydrogenase	0.00	-5.15	1.04
2775	215487851	xseA;exonuclease VII, large	0.00	-4.55	0.46
2776	215487852	-;RatA-like protein	0.00	2.34	0.27
2779	215487855	-;hypothetical protein	0.00	2.85	0.49
2780	215487856	-;hypothetical protein	0.00	2.75	0.30
2782	215487858	-;hypothetical protein	0.00	2.60	0.53
2784	215487860	-;predicted integrase	0.00	2.40	0.39

2785	215487861	der;predicted GTP-binding	0.01	2.08	0.50
2789	215487865	ispG;1-hydroxy-2-methyl-2-(E)-butenyl 4-diphosphate	0.00	-4.04	0.76
2792	215487868	ndk;multifunctional nucleoside diphosphate kinase and apyrimidinic endonuclease and 3'-phosphodiesterase	0.00	14.33	0.86
2798	215487874	iscX;hypothetical protein	0.00	2.60	0.47
2801	215487877	hscB;DnaJ-like molecular chaperone specific for IscU	0.00	2.68	0.42
2804	215487880	iscS;cysteine desulfurase (tRNA sulfurtransferase), PLP-	0.00	7.91	0.18
2805	215487881	iscR;DNA-binding transcriptional repressor	0.00	8.08	0.73
2806	215487882	trmJ;predicted methyltransferase	0.00	2.97	0.54
2807	215487883	suhB;inositol monophosphatase	0.01	2.21	0.36
2809	215487885	csiE;stationary phase inducible	0.00	-4.27	0.41
2819	215487895	glyA;serine	0.00	3.25	0.11
2821	215487897	glnB;regulatory protein P-II for glutamine synthetase	0.00	4.76	0.55
2823	215487899	yfhG;hypothetical protein	0.00	3.20	0.35
2824	215487900	qseE;predicted sensory kinase in two-component system with	0.00	-2.29	0.07
2826	215487902	yfhD;predicted transglycosylase	0.02	2.05	0.16
2827	215487903	tadA;tRNA-specific adenosine deaminase	0.00	2.53	0.07
2829	215487905	yfhH;predicted DNA-binding transcriptional regulator	0.00	12.69	0.95
2830	215487906	yfhL;predicted 4Fe-4S cluster-containing protein	0.00	6.56	0.64
2834	215487910	era;membrane-associated, 16S rRNA-binding GTPase	0.01	2.11	0.37
2835	215487911	rnc;RNase III	0.00	2.78	0.50
2839	215487915	rseB;anti-sigma factor RseB	0.01	-2.02	0.31
2846	215487922	yfiD;pyruvate formate lyase	0.00	-2.81	0.71
2847	215487923	ung;uracil-DNA-glycosylase	0.00	5.28	0.32
2849	215487925	trxC;thioredoxin 2	0.00	3.88	0.53
2850	215487926	yfiP;hypothetical protein	0.00	-4.75	0.72
2853	215487929	yfiM;hypothetical protein	0.01	2.06	0.29
2855	215487931	clpB;protein disaggregation	0.02	-2.04	0.69
2861	215487938	tyrA;fused chorismate mutase T/prephenate dehydrogenase	0.00	-2.55	0.25
2865	215487942	yfiN;predicted diguanylate	0.01	-2.18	0.44
2870	215487947	-;predicted DNA-invertase	0.00	-3.83	0.51
2873	215487950	-;predicted tail protein	0.00	-4.91	0.31
2876	215487953	-;predicted late gene regulator	0.00	2.32	0.24
2877	215487954	-;predicted DNA-binding transcriptional regulator	0.00	6.65	1.02
2878	215487955	-;hypothetical protein	0.00	3.11	0.75
2884	215487961	ypjD;predicted inner membrane	0.00	3.45	0.16
2886	215487963	grpE;heat shock protein	0.00	2.48	0.27
2889	215487966	smpA;small membrane	0.00	10.15	0.95
2890	215487967	yfjF;hypothetical protein	0.00	3.56	0.35
2891	215487968	yfjG;hypothetical protein	0.00	5.99	0.35
2893	215487970	-;hypothetical protein	0.00	5.11	0.05
2896	215487973	-;T3SS secreted effector EspG homolog	0.01	2.35	0.06
2897	215487974	-;hypothetical protein	0.00	2.36	0.49
2902	215487979	csiD;hypothetical protein	0.00	-4.76	0.05
2903	215487980	ihgO;L-2-hydroxyglutarate	0.00	-5.47	0.49
2904	215487981	gabD;succinate-semialdehyde dehydrogenase I, NADP-	0.00	-5.90	0.11
2905	215487982	gabT;4-aminobutyrate aminotransferase, PLP-	0.00	-5.10	0.20
2906	215487983	gabP;gamma-aminobutyrate	0.00	-2.53	0.21
2908	215487985	ygaU;hypothetical protein	0.00	-2.67	0.31
2910	215487987	ygaV;predicted DNA-binding transcriptional regulator	0.00	2.34	0.21
2911	215487988	ygaP;predicted inner membrane protein with hydrolase activity	0.01	-2.01	0.15

2912	215487989	stpA;DNA binding protein, nucleoid-associated	0.00	8.10	0.88
2913	215487990	ygaW;predicted inner membrane	0.00	2.51	0.61
2914	215487991	ygaC;hypothetical protein	0.00	4.04	0.69
2918	215487995	nrdI;NrdI protein that stimulates ribonucleotide reduction	0.00	2.56	0.18
2919	215487996	nrdE;ribonucleoside-diphosphate reductase 2, alpha subunit	0.00	-2.74	0.61
2920	215487997	nrdF;ribonucleoside-diphosphate reductase 2, beta subunit,	0.00	-3.21	0.89
2921	215487998	proV;glycine betaine transporter subunit	0.00	-2.35	0.26
2922	215487999	proW;high-affinity transport system for glycine betaine and proline; membrane component	0.01	-2.04	0.35
2931	215488008	-;hypothetical protein	0.00	-2.59	0.72
2935	215488014	ygaB;predicted hydrolase	0.00	6.42	0.45
2940	215488019	ygaD;hypothetical protein	0.00	3.10	0.16
2941	215488020	mltB;membrane-bound lytic murein transglycosylase B	0.00	3.26	0.28
2942	215488021	gutQ;predicted phosphosugar-binding protein	0.00	2.42	0.14
2944	215488023	norV;flavorubredoxin	0.01	-2.17	0.68
2946	215488027	-;transposase Orf1 of IS3 family IS element	0.00	2.64	0.51
2948	215488031	-;hypothetical protein	0.00	2.29	0.67
2949	215488032	-;hypothetical protein	0.01	2.06	0.13
2950	215488033	hycI;protease involved in processing C-terminal end of	0.00	5.76	0.29
2954	215488037	hycE;hydrogenase 3, large	0.02	-2.29	0.47
2956	215488039	hycC;hydrogenase 3, membrane subunit	0.00	-2.28	0.36
2957	215488040	hycB;hydrogenase 3, Fe-S	0.00	-2.65	0.50
2959	215488042	hypA;protein involved in nickel insertion into hydrogenases 3	0.00	2.83	0.49
2963	215488046	hypE;carbamoyl phosphate phosphatase, hydrogenase 3 maturation protein HypE	0.00	-3.34	0.52
2964	215488047	fhlA;DNA-binding transcriptional activator	0.00	-2.83	0.46
2969	215488052	ygbI;predicted DNA-binding transcriptional regulator	0.00	7.80	0.43
2970	215488053	ygbJ;predicted dehydrogenase, with NAD(P)-binding Rossmann-fold domain	0.01	2.34	0.30
2976	215488059	-;predicted 4-hydroxybenzoate decarboxylase	0.00	-2.22	0.49
2978	215488061	-;transcriptional regulator (MarR-family)	0.00	4.43	0.79
2979	215488062	nlpD;predicted outer membrane lipoprotein	0.00	5.69	0.75
2980	215488063	pcm;L-isoaspartate protein carboxylmethyltransferase type II	0.00	2.91	0.22
2982	215488065	truD;pseudouridine synthase	0.00	4.46	0.30
2983	215488066	ispF;2C-methyl-D-erythritol 2,4-cyclodiphosphate synthase	0.00	2.86	0.26
2985	215488068	ftsB;cell division protein	0.01	2.24	0.46
2986	215488069	ygbE;conserved inner membrane protein	0.00	4.45	0.51
2989	215488072	cysD;sulfate adenylyltransferase, subunit 2	0.00	2.41	0.52
2990	215488073	iap;aminopeptidase in alkaline phosphatase isozymeconversion	0.00	3.08	0.37
2992	215488075	cysH;3'-phosphoadenosine 5'-phosphosulfate reductase	0.01	2.01	0.16
2993	215488076	cysI;sulfite reductase, beta subunit, NAD(P)-binding, heme-	0.00	-5.34	0.92
2995	215488078	sscR;6-pyruvoyl tetrahydrobiopterin synthase	0.00	11.98	0.34
3004	215488087	yqcE;predicted transporter	0.01	-2.04	0.21
3010	215488093	scrR;sucrose operon repressor	0.00	2.47	0.21

3012	215488095	ygcG;hypothetical protein	0.00	-2.50	0.16
3013	215488096	eno;enolase	0.01	-2.35	0.88
3015	215488098	mazG;nucleoside triphosphate pyrophosphohydrolase	0.01	2.04	0.50
3016	215488099	chpA;toxin ChpA of the ChpA-ChpR toxin-antitoxin system, endoribonuclease	0.00	3.90	0.47
3017	215488100	chpR;antitoxin ChpR of the ChpA-ChpR toxin-antitoxin	0.00	3.55	0.69
3018	215488101	relA;(p)ppGpp synthetase I/GTP pyrophosphokinase	0.00	-5.08	0.31
3023	215488106	gudP;predicted D-glucarate	0.00	-6.75	0.45
3025	215488108	truC;tRNA pseudouridine	0.00	6.23	0.05
3026	215488109	ygcC;hypothetical protein	0.00	6.52	0.50
3029	215488112	ygdH;hypothetical protein	0.00	-2.47	0.39
3032	215488115	xni;exonuclease IX (5'-3' exonuclease)	0.00	4.64	0.58
3038	215488121	fucU;L-fucose mutarotase	0.01	-2.20	0.30
3043	215488126	ygdI;hypothetical protein	0.00	-3.49	0.71
3045	215488128	ygdK;predicted Fe-S metabolism protein	0.00	2.16	0.37
3047	215488130	mltA;membrane-bound lytic murein transglycosylase A	0.00	6.89	0.28
3049	215488132	-;phosphosugar isomerase	0.00	-2.12	0.22
3057	215488140	recC;exonuclease V (RecBCD complex), gamma chain	0.00	-2.55	0.98
3059	215488142	ygdB;hypothetical protein	0.00	2.14	0.02
3063	215488146	lgt;phosphatidylglycerol-prolipoprotein diacylglyceryl	0.01	-2.19	0.19
3068	215488151	ygdQ;predicted inner membrane	0.00	2.38	0.54
3070	215488153	tas;predicted oxidoreductase, NADP(H)-dependent aldo-keto reductase	0.00	2.29	0.20
3072	215488155	aas;fused 2-acylglycerophospho-ethanolamine acyl	0.01	-2.02	0.61
3073	215488156	galR;DNA-binding transcriptional repressor	0.00	-3.23	0.55
3077	215488160	araE;arabinose transporter	0.01	-2.15	0.32
3078	215488162	kduD;2-deoxy-D-gluconate 3-dehydrogenase	0.01	-2.05	0.18
3080	215488164	yqeF;predicted acyltransferase	0.00	9.23	0.41
3082	215488166	ygeR;tetratricopeptide repeat transcriptional regulator	0.00	5.57	0.76
3084	215488168	xdhB;xanthine dehydrogenase, FAD-binding subunit	0.02	-2.10	0.83
3093	215488177	yqeC;hypothetical protein	0.04	2.04	0.37
3094	215488178	ygfJ;hypothetical protein	0.00	2.28	0.90
3095	215488180	ssnA;predicted chlorohydrolase/aminohydrolase	0.00	-4.59	0.50
3096	215488181	ygfM;predicted oxidoreductase	0.00	-3.58	0.07
3098	215488183	guaD;guanine deaminase	0.00	-2.67	1.00
3100	215488185	ygfS;predicted oxidoreductase, 4Fe-4S ferredoxin-type subunit	0.02	-2.04	0.95
3103	215488188	-;hypothetical protein	0.00	3.35	1.41
3104	215488189	idi;isopentenyl diphosphate	0.01	2.11	0.46
3109	215488194	xerD;site-specific tyrosine recombinase	0.00	2.38	0.12
3110	215488195	fldB;flavodoxin 2	0.00	3.43	0.45
3111	215488196	ygfX;hypothetical protein	0.00	6.93	0.19
3112	215488197	ygfY;hypothetical protein	0.00	3.22	0.82
3115	215488200	yqfB;hypothetical protein	0.00	2.53	0.46
3122	215488207	pepP;proline aminopeptidase P II	0.02	-2.02	0.59
3123	215488208	ygfB;hypothetical protein	0.00	3.80	0.39
3127	215488212	rpiA;ribose 5-phosphate isomerase, constitutive	0.00	5.15	0.25
3130	215488215	yggE;hypothetical protein	0.01	2.05	0.64
3135	215488220	epd;D-erythrose 4-phosphate dehydrogenase	0.00	5.79	0.20
3142	215488227	yggF;predicted hexose P	0.00	-3.21	0.14

3144	215488229	cmtA;predicted fused mannitol-specific PTS enzymes: IIB component/IIC component	0.00	-3.40	0.55
3146	215488231	-;HflC-like, SPFC domain-containing protein	0.01	2.01	0.33
3149	215488234	speB;agmatinase	0.00	-2.75	0.48
3153	215488238	yqgC;hypothetical protein	0.01	2.00	0.48
3160	215488245	gshB;glutathione synthetase	0.00	2.13	0.13
3161	215488246	yqgE;hypothetical protein	0.00	2.52	0.33
3162	215488247	yqgF;predicted Holliday junction resolvase	0.02	-2.20	0.26
3164	215488249	yggS;predicted enzyme	0.01	2.03	0.32
3165	215488250	yggT;predicted inner membrane	0.00	2.90	0.20
3166	215488251	yggU;hypothetical protein	0.01	2.01	0.08
3171	215488256	yggN;hypothetical protein	0.00	2.52	0.32
3172	215488257	yggL;hypothetical protein	0.00	2.50	0.17
3173	215488258	trmI;tRNA (m7G46) methyltransferase, SAM-	0.00	2.63	0.37
3174	215488259	mutY;adenine DNA glycosylase	0.00	2.21	0.46
3175	215488260	yggX;protein that protects iron-sulfur proteinsagainst oxidative	0.00	6.30	0.38
3179	215488264	yqgA;predicted inner membrane	0.00	-2.78	0.64
3184	215488269	-;hypothetical protein	0.00	-2.31	0.68
3193	215488278	-;predicted transposase	0.00	2.71	0.50
3196	215488281	gspK;putative type II secretion	0.00	-2.20	0.62
3199	215488284	gspH;putative type II secretion	0.00	-3.20	0.02
3202	215488287	gspE;putative type II secretion	0.00	-3.03	0.62
3205	215488290	yghG;hypothetical protein	0.00	2.79	0.47
3210	215488295	glcG;hypothetical protein	0.00	2.22	0.34
3213	215488298	glcD;glycolate oxidase subunit, FAD-linked	0.00	-3.10	0.94
3214	215488299	glcC;DNA-binding transcriptional dual regulator, glycolate-binding	0.00	2.97	0.25
3226	215488311	yghS;conserved predicted protein with nucleoside triphosphate hydrolase domain	0.00	-3.03	0.35
3227	215488312	yghT;conserved predicted protein with nucleoside triphosphate hydrolase domain	0.00	-3.05	0.74
3228	215488313	pitB;phosphate transporter	0.00	-4.54	0.08
3233	215488318	hybE;hydrogenase 2-specific chaperone HybE	0.00	-3.61	0.17
3234	215488319	hybD;predicted maturation element for hydrogenase 2	0.00	-3.57	0.16
3236	215488321	hybB;predicted hydrogenase 2 cytochrome b type component	0.00	-2.42	1.03
3239	215488324	yghW;hypothetical protein	0.00	-3.24	1.08
3242	215488328	yqhA;conserved inner membrane protein	0.00	11.99	0.44
3248	215488334	yqhC;predicted DNA-binding transcriptional regulator	0.00	2.61	0.11
3250	215488336	dkgA;2,5-diketo-D-gluconate reductase A	0.00	-5.33	0.74
3252	215488338	yqhH;predicted outer membrane lipoprotein	0.02	2.40	0.44
3254	215488340	-;predicted regulator	0.00	5.17	0.27
3255	215488341	-;predicted mannitol dehydrogenase-family protein	0.00	-3.32	0.48
3258	215488344	-;predicted TRAP-type C4-dicarboxylate transport system, periplasmic component	0.00	-5.74	0.87
3261	215488347	sufI;repressor protein for FtsI	0.00	2.25	0.42
3265	215488351	ygiV;predicted transcriptional	0.01	2.08	0.43
3270	215488356	ygiN;quinol monooxygenase	0.01	2.29	0.79
3272	215488358	-;predicted ferrichrome-binding	0.00	-3.67	0.58
3278	215488364	ygiA;predicted esterase	0.00	2.15	0.17
3279	215488365	cpdA;cyclic 3',5'-adenosine monophosphate	0.00	4.15	0.62
3281	215488367	nudF;ADP-ribose	0.00	4.53	0.55
3284	215488370	ygiB;conserved outer membrane protein	0.01	2.13	0.40

3287	215488373	-;predicted disulfide isomerase, DsbA family	0.00	-4.28	0.57
3292	215488378	ribB;3,4-dihydroxy-2-butanone-4-phosphate synthase	0.00	2.35	1.29
3293	215488379	yqiC;hypothetical protein	0.00	4.38	0.52
3294	215488380	glgS;predicted glycogen synthesis protein	0.01	-2.20	0.41
3297	215488383	rfaE;fused heptose 7-phosphate kinase/heptose1-phosphate adenyltransferase	0.01	2.04	0.30
3298	215488384	glnE;fused deadenylyltransferase/adenylyltransferase for glutamine	0.00	-2.44	1.13
3300	215488386	htrG;predicted signal transduction protein	0.00	8.63	0.10
3302	215488388	bacA;undecaprenyl pyrophosphate phosphatase	0.00	2.18	0.16
3305	215488391	ttdR;predicted DNA-binding transcriptional regulator	0.00	-5.03	0.60
3309	215488395	ygjD;predicted peptidase	0.02	2.01	0.47
3310	215488396	rpsU;30S ribosomal subunit	0.00	4.08	1.19
3313	215488399	-;hypothetical protein	0.00	-2.30	0.14
3317	215488403	ygjG;putrescine: 2-oxoglutaric acid aminotransferase, PLP-	0.00	-3.88	0.13
3318	215488404	ygjH;hypothetical protein	0.00	4.49	0.40
3324	215488410	ygjK;predicted glycosyl hydrolase	0.00	-2.83	0.17
3325	215488411	fadH;2,4-dienoyl-CoA reductase, NADH and FMN-	0.00	-2.39	0.64
3326	215488412	ygjM;predicted DNA-binding transcriptional regulator	0.00	4.68	0.10
3328	215488414	ygjP;predicted metal dependent hydrolase	0.00	6.01	0.50
3336	215488423	exuT;hexuronate transporter	0.00	-2.37	0.41
3340	215488427	yqjC;hypothetical protein	0.00	4.29	0.21
3346	215488433	yhaH;predicted inner membrane	0.01	2.17	0.94
3347	215488434	yhaJ;predicted DNA-binding transcriptional regulator	0.01	2.13	0.70
3349	215488436	yhaL;hypothetical protein	0.00	6.63	1.40
3352	215488439	tdcG;L-serine dehydratase 3	0.00	-2.16	0.61
3353	215488440	tdcF;predicted L-PSP (mRNA) endoribonuclease	0.02	2.55	0.37
3356	215488443	tdcC;L-threonine/L-serine	0.01	-2.13	0.57
3358	215488445	tdcA;DNA-binding transcriptional activator	0.00	10.02	0.60
3359	215488446	tdcR;DNA-binding transcriptional activator	0.04	4.06	1.49
3362	215488449	garL;alpha-dehydro-beta-deoxy-D-glucarate aldolase	0.00	2.32	0.15
3364	215488451	garD;(D)-galactarate	0.01	2.19	0.56
3367	215488454	agaV;N-acetylgalactosamine-specific enzyme IIB component	0.00	-3.37	0.28
3372	215488459	agaS;tagatose-6-phosphate ketose/aldose isomerase	0.00	-2.35	0.28
3373	215488460	kbaY;tagatose 6-phosphate aldolase 1, KbaY subunit	0.00	-2.70	0.45
3375	215488462	agaC;N-acetylgalactosamine-specific enzyme IIC component	0.00	-2.72	0.72
3379	215488466	yraM;hypothetical protein	0.01	-2.12	0.37
3381	215488468	diaA;DnaA initiator-associating factor for replication initiation	0.00	2.34	0.45
3382	215488469	yraP;hypothetical protein	0.00	2.24	0.14
3386	215488473	yhbQ;predicted endonuclease	0.00	3.95	0.80
3388	215488475	yhbT;predicted lipid carrier	0.00	2.76	0.13
3393	215488480	deaD;ATP-dependent RNA	0.00	-2.72	0.14
3396	215488483	rpsO;30S ribosomal subunit	0.00	6.24	0.67
3399	215488486	infB;fused protein chain initiation factor 2, IF2: membrane protein/conserved protein	0.01	-2.26	0.29
3401	215488488	yhbC;hypothetical protein	0.00	2.60	0.35
3402	215488489	argG;argininosuccinate	0.00	2.55	0.53

3404	215488491	secG;preprotein translocase membrane subunit	0.00	2.88	0.37
3405	215488492	glmM;phosphoglucosamine	0.00	3.66	0.39
3409	215488496	yhbY;predicted RNA-binding	0.00	13.29	0.37
3410	215488497	greA;transcription elongation	0.01	2.05	0.04
3414	215488501	rpmA;50S ribosomal subunit	0.00	2.30	0.45
3415	215488502	rplU;50S ribosomal subunit	0.00	3.54	0.58
3416	215488503	ispB;octaprenyl diphosphate	0.00	3.80	0.31
3419	215488506	yrbA;predicted DNA-binding transcriptional regulator	0.00	3.91	0.43
3424	215488511	yrbF;predicted toluene transporter subunit: ATP-binding component of ABC superfamily	0.00	-2.59	0.70
3425	215488512	yrbG;predicted calcium/sodium: proton antiporter	0.00	3.07	0.37
3426	215488513	kdsD;D-arabinose 5-phosphate isomerase	0.00	2.29	0.23
3431	215488518	rpoN;RNA polymerase, sigma 54 (sigma N) factor	0.00	-2.53	0.37
3432	215488519	hpf;predicted ribosome-associated, sigma 54	0.00	-2.97	0.67
3433	215488520	ptsN;sugar-specific enzyme IIA component of PTS	0.00	-2.60	0.20
3435	215488522	npr;phosphohistidinoprotein-hexose phosphotransferase component of N-regulated PTS	0.00	3.79	0.33
3436	215488523	yrbL;hypothetical protein	0.00	4.05	0.32
3440	215488527	yhcC;predicted Fe-S	0.00	-3.16	0.32
3445	215488532	nanE;predicted N-acetylmannosamine-6-P	0.00	-3.01	0.25
3448	215488535	nanR;DNA-binding transcriptional dual regulator	0.00	5.26	0.65
3449	215488536	sspB;ClpXP protease specificity-enhancing factor	0.00	-3.59	0.24
3450	215488537	sspA;stringent starvation protein	0.00	2.58	0.23
3452	215488539	rplM;50S ribosomal subunit	0.01	2.03	0.47
3454	215488541	yhcB;hypothetical protein	0.01	2.20	0.36
3456	215488543	degS;serine endoprotease,	0.00	5.18	0.13
3457	215488544	mdh;malate dehydrogenase, NAD(P)-binding	0.00	3.14	0.51
3459	215488546	yhcN;hypothetical protein	0.00	6.12	0.53
3465	215488553	tldD;predicted peptidase	0.02	-2.06	0.14
3466	215488554	yhdP;conserved membrane protein, predicted transporter	0.00	-2.73	0.51
3468	215488556	yhdE;hypothetical protein	0.00	2.67	0.31
3471	215488559	mreB;cell wall structural complex MreBCD, actin-like component	0.01	2.05	0.12
3472	215488560	csrD;conserved inner membrane protein	0.00	4.70	0.12
3477	215488565	panF;pantothenate: sodium	0.00	-2.59	0.72
3480	215488568	fis;global DNA-binding transcriptional dual regulator	0.00	2.14	0.52
3482	215488570	yhdU;predicted membrane	0.00	11.75	0.51
3483	215488571	envR;DNA-binding transcriptional regulator	0.01	-2.07	0.89
3503	215488591	yhdL;hypothetical protein	0.00	4.00	0.25
3513	215488601	rplO;50S ribosomal subunit	0.01	2.09	0.29
3514	215488602	rpmD;50S ribosomal subunit	0.01	2.06	0.28
3515	215488603	rpsE;30S ribosomal subunit	0.01	2.22	0.08
3516	215488604	rplR;50S ribosomal subunit	0.00	2.12	0.43
3517	215488605	rplF;50S ribosomal subunit	0.02	2.04	0.23
3520	215488608	rplE;50S ribosomal subunit	0.01	2.14	0.34
3521	215488609	rplX;50S ribosomal subunit	0.00	2.58	0.39
3522	215488610	rplN;50S ribosomal subunit	0.00	3.77	0.24
3533	215488621	rpsJ;30S ribosomal subunit	0.01	2.06	0.43
3535	215488623	bfr;bacterioferritin, iron storage and detoxification protein	0.00	6.41	0.92
3536	215488624	bfd;bacterioferritin-associated ferredoxin	0.00	13.37	0.67
3543	215488632	yheO;predicted DNA-binding transcriptional regulator	0.00	5.38	0.04

3544	215488633	fkpA;FKBP-type peptidyl-prolyl cis-trans isomerase (rotamase)	0.00	2.34	0.43
3545	215488634	slyX;hypothetical protein	0.00	8.32	0.61
3546	215488635	slyD;FKBP-type peptidyl prolyl cis-trans isomerase (rotamase)	0.00	2.68	0.25
3547	215488636	yheV;hypothetical protein	0.00	-2.64	0.44
3552	215488641	yheU;hypothetical protein	0.00	2.53	0.37
3554	215488643	yhfA;hypothetical protein	0.00	4.90	0.31
3555	215488644	crp;DNA-binding transcriptional dual regulator	0.00	4.34	0.22
3561	215488650	ppiA;peptidyl-prolyl cis-trans isomerase A (rotamase A)	0.00	7.52	0.24
3562	215488651	tsgA;predicted transporter	0.00	-2.55	0.34
3563	215488652	nirB;nitrite reductase, large subunit, NAD(P)H-binding	0.01	-2.04	0.62
3565	215488654	nirC;nitrite transporter	0.00	-2.22	0.44
3568	215488657	yhfS;hypothetical protein	0.00	-2.47	0.44
3570	215488659	yhfU;hypothetical protein	0.00	-3.33	0.43
3572	215488661	yhfW;predicted mutase	0.00	-2.22	0.35
3576	215488665	trpS;tryptophanyl-tRNA	0.00	3.71	0.28
3581	215488670	aroB;3-dehydroquinate synthase	0.00	-2.71	0.61
3588	215488677	nudE;ADP-ribose diphosphatase	0.00	5.37	0.41
3590	215488679	yrfG;predicted hydrolase	0.00	4.91	0.73
3591	215488680	hslR;ribosome-associated heat shock protein Hsp15	0.00	2.63	0.97
3594	215488683	pck;phosphoenolpyruvate carboxykinase	0.00	3.54	0.52
3598	215488687	greB;transcription elongation	0.00	2.33	0.66
3600	215488689	feoA;ferrous iron transporter,	0.00	5.13	0.68
3604	215488693	bioH;carboxylesterase of pimeloyl-CoA synthesis	0.00	3.10	0.26
3606	215488695	gntY;predicted gluconate transport associated protein	0.00	4.76	0.03
3615	215488704	glpG;predicted intramembrane serine protease	0.00	2.71	0.29
3616	215488705	glpE;thiosulfate: cyanide sulfurtransferase (rhodanese)	0.00	2.48	0.32
3617	215488706	glpD;sn-glycerol-3-phosphate dehydrogenase, aerobic, FAD/NAD(P)-binding	0.00	-2.45	0.24
3620	215488709	glgP;glycogen phosphorylase	0.00	-2.30	0.12
3624	215488713	glgB;1,4-alpha-glucan branching enzyme GlgB	0.00	-2.52	0.23
3630	215488719	yhhW;hypothetical protein	0.00	3.24	0.57
3635	215488724	ugpQ;glycerophosphodiester phosphodiesterase UgpQ,	0.01	-2.28	0.88
3636	215488725	ugpC;ATP-binding component of sn-glycerol 3-phosphate	0.00	-2.58	0.47
3637	215488726	ugpE;glycerol-3-phosphate transporter subunit	0.00	-2.94	0.16
3641	215488730	livG;leucine/isoleucine/valine transporter subunit	0.00	-4.40	0.72
3642	215488731	livM;high-affinity branched-chain amino acid transport; membrane component of ABC superfamily	0.02	-2.03	0.51
3643	215488732	livH;high-affinity branched-chain amino acid transport system; membrane component; membrane component of ABC	0.01	-2.01	0.96
3644	215488733	livK;leucine transporter subunit	0.01	-2.09	0.26
3645	215488734	yhhK;hypothetical protein	0.00	2.27	0.42
3647	215488736	rpoH;RNA polymerase, sigma 32 (sigma H) factor	0.00	2.34	0.28
3649	215488738	ftsE;predicted transporter subunit: ATP-binding component of ABC superfamily FtsE	0.00	2.90	0.09
3653	215488742	yhhM;hypothetical protein	0.00	5.25	0.45
3655	215488744	zntA;zinc, cobalt and lead efflux system	0.00	-3.99	0.97
3656	215488746	sirA;conserved protein required for cell growth	0.00	10.90	1.08

3658	215488748	dcrB;periplasmic protein	0.00	6.46	0.09
3660	215488750	yhhT;predicted inner membrane	0.01	−2.17	0.50
3664	215488754	nikC;transport of nickel, membrane protein NikC; membrane component of ABC	0.00	−2.42	0.43
3667	215488757	nikR;DNA−binding transcriptional repressor, Ni−binding	0.00	3.38	0.77
3669	215488759	−;HicA−like protein	0.00	−2.58	0.74
3672	215488762	yhiI;predicted HlyD family secretion protein	0.00	3.81	0.27
3673	215488763	yhiM;conserved inner membrane protein	0.01	−2.22	0.02
3674	215488764	yhiN;predicted oxidoreductase with FAD/NAD(P)−binding	0.00	2.53	0.16
3675	215488765	pitA;phosphate transporter, low−	0.00	3.58	0.12
3678	215488768	yhiP;predicted transporter	0.00	2.23	0.75
3679	215488769	yhiQ;predicted SAM−dependent methyltransferase	0.00	−3.57	0.58
3681	215488771	yhiR;predicted DNA (exogenous) processing protein	0.00	2.53	0.44
3685	215488775	slp;outer membrane lipoprotein	0.00	−2.58	0.31
3686	215488776	yhiF;predicted DNA−binding transcriptional regulator	0.00	−9.15	0.25
3689	215488779	chuT;putative hemin binding	0.00	−2.10	0.35
3695	215488785	yhiD;predicted Mg(2+) transport ATPase inner membrane protein	0.00	−3.51	0.39
3698	215488788	hdeD;acid−resistance membrane protein	0.00	−6.28	0.47
3699	215488789	gadE;DNA−binding transcriptional activator	0.00	−2.46	0.20
3701	215488791	mdtF;multidrug transporter, RpoS−dependent	0.00	−4.69	0.24
3704	215488794	gadA;glutamate decarboxylase A, PLP−dependent	0.00	−9.29	0.94
3706	215488796	treF;cytoplasmic trehalase	0.00	−3.48	0.23
3715	215488805	dctA;C4−dicarboxylic acid, orotate and citrate transporter	0.00	7.93	0.34
3718	215488808	bcsZ;endo−1,4−D−glucanase	0.00	−2.25	0.44
3719	215488809	bcsB;regulator of cellulose synthase, cyclic di−GMP binding	0.00	−3.05	0.45
3721	215488811	yhjQ;cell division protein (chromosome partitioning	0.00	2.55	0.61
3722	215488812	yhjR;hypothetical protein	0.01	2.07	0.85
3723	215488813	bcsE;hypothetical protein	0.00	−2.64	0.71
3724	215488814	bcsF;hypothetical protein	0.00	−2.06	0.30
3730	215488820	−;hypothetical protein	0.00	−2.72	0.40
3735	215488825	dppC;dipeptide transporter	0.02	−2.01	0.47
3737	215488827	dppA;dipeptide transporter	0.01	−2.09	0.19
3740	215488830	lpfD;predicted fimbrial protein	0.02	−2.19	0.46
3741	215488831	lpfC;predicted fimbrial usher	0.00	−2.06	0.28
3749	215488839	yiaF;hypothetical protein	0.00	7.71	0.33
3753	215488843	glyS;glycine tRNA synthetase, beta subunit	0.00	−2.15	0.07
3755	215488845	ysaB;hypothetical protein	0.00	3.43	0.20
3757	215488847	yiaA;conserved inner membrane protein	0.00	−2.76	0.44
3775	215488865	yiaO;predicted transporter, periplasmic component	0.00	−4.22	0.67
3790	215488880	mtlR;DNA−binding repressor MtlR	0.01	2.04	0.03
3792	215488882	yibL;hypothetical protein	0.00	6.25	0.85
3796	215488886	yibK;predicted rRNA methylase	0.00	3.18	0.11
3797	215488887	cysE;serine acetyltransferase	0.00	2.23	0.21
3800	215488890	grxC;glutaredoxin 3	0.00	3.17	0.16
3801	215488891	yibN;predicted rhodanese−related sulfurtransferase	0.00	2.33	0.55
3804	215488894	yibQ;predicted polysaccharide deacetylase	0.00	7.95	0.36
3821	215488911	rfaQ;lipopolysaccharide core biosynthesis glycosyl transferase	0.01	2.09	0.50
3823	215488913	coaD;pantetheine−phosphate adenylyltransferase	0.00	2.50	0.35

3825	215488915	rpmG;50S ribosomal subunit	0.01	2.17	0.43
3826	215488916	rpmB;50S ribosomal subunit	0.00	2.63	0.24
3827	215488917	yicR;protein associated with replication fork, possible DNA	0.00	5.16	0.36
3828	215488918	dfp;fused 4'-phosphopantothenoylcysteine decarboxylase/phosphopantothenoylcysteine synthetase, FMN-	0.00	3.01	0.73
3829	215488919	dut;deoxyuridinetriphosphatase	0.00	6.10	0.10
3830	215488920	slmA;division inhibitor	0.00	2.41	0.03
3831	215488921	-;predicted RNA-directed DNA polymerase	0.00	2.44	0.59
3834	215488924	yicC;hypothetical protein	0.00	2.95	0.10
3841	215488931	-;hypothetical protein	0.00	3.49	0.92
3845	215488935	-;hypothetical protein	0.00	-2.62	0.37
3846	215488936	-;hypothetical protein	0.00	-2.95	0.60
3847	215488937	-;hypothetical protein	0.00	-4.05	0.87
3850	215488940	dinD;DNA-damage-inducible	0.00	2.93	0.76
3858	215488948	gltS;glutamate transporter	0.00	4.42	0.25
3859	215488949	yicE;predicted transporter	0.00	-2.91	0.48
3860	215488950	yicH;hypothetical protein	0.01	-2.10	0.39
3876	215488966	espD;translocon EspD	0.00	2.29	0.21
3879	215488969	escD;T3SS structure protein	0.00	-5.30	0.45
3883	215488973	map;LEE-encoded effector Map	0.00	4.34	0.49
3888	215488978	-;hypothetical protein	0.00	-4.21	0.37
3890	215488980	escV;translocator EscV	0.00	-2.25	0.14
3892	215488982	espZ;LEE-encoded effector	0.00	2.76	1.33
3895	215488985	sepD;secretion switching protein	0.03	2.36	0.39
3896	215488986	escC;T3SS structure protein	0.00	-2.22	0.46
3900	215488990	-;hypothetical protein	0.00	-2.35	0.46
3903	215488993	escS;T3SS structure protein	0.00	-3.02	0.94
3904	215488994	escR;T3SS structure protein	0.00	-2.97	1.12
3905	215488995	-;component of T3SS	0.01	-2.20	0.93
3907	215488997	-;component of T3SS	0.00	-2.62	1.07
3915	215489005	yicL;predicted inner membrane	0.02	-2.08	0.17
3917	215489007	-;hypothetical protein	0.01	2.13	0.64
3924	215489014	uhpA;DNA-binding response regulator UhpA in two-component regulatory system	0.00	2.38	0.36
3928	215489018	emrD;multidrug efflux system	0.00	3.35	0.28
3929	215489019	yidF;predicted DNA-binding transcriptional regulator	0.00	2.31	0.22
3930	215489020	yidG;predicted inner membrane	0.00	3.85	0.73
3932	215489022	yidJ;predicted	0.03	2.17	0.28
3933	215489023	yidK;predicted transporter	0.00	-2.94	0.82
3936	215489026	ibpB;heat shock chaperone	0.00	8.03	0.61
3937	215489027	ibpA;heat shock chaperone	0.00	3.96	0.47
3938	215489028	yidQ;conserved outer membrane protein	0.00	6.23	0.39
3941	215489031	dgoT;D-galactonate transporter	0.01	2.30	0.17
3947	215489038	yidB;hypothetical protein	0.00	2.46	0.59
3949	215489040	recF;gap repair protein	0.00	-2.57	0.62
3950	215489041	dnaN;DNA polymerase III, beta	0.00	-2.93	0.37
3951	215489042	dnaA;chromosomal replication initiator protein DnaA, DNA-binding transcriptional dual	0.00	3.04	0.10
3952	215489043	rpmH;50S ribosomal subunit	0.00	2.83	1.16
3953	215489044	rnpA;protein C5 component of	0.02	2.18	0.58
3955	215489046	mnmE;GTPase	0.00	2.73	0.52
3956	215489047	tanC;tryptophanase leader peptide TnaC	0.00	6.12	2.05
3959	215489050	mdtL;multidrug efflux system	0.00	-2.24	0.25
3970	215489061	bglG;transcriptional antiterminator of the bgl operon	0.01	2.65	0.39
3971	215489062	phoU;negative regulator of PhoR/PhoB two-component	0.00	2.91	0.34
3972	215489063	pstB;ATP-binding component of high-affinity phosphate-specific transport system; ATP-binding component PstB of ABC	0.00	2.41	0.50

3973	215489064	pstA;high-affinity phosphate-specific transport system, cytoplasmic membrane component; membrane	0.00	2.49	0.40
3975	215489066	pstS;high-affinity phosphate-specific transport system; periplasmic phosphate-binding protein; periplasmic-binding component PstS of ABC	0.00	2.28	0.17
3978	215489069	atpC;F1 sector of membrane-bound ATP synthase, epsilon	0.01	2.03	0.48
3982	215489073	atpH;F1 sector of membrane-bound ATP synthase, delta	0.00	3.50	0.18
3983	215489074	atpF;F0 sector of membrane-bound ATP synthase, subunit b	0.00	4.21	0.18
3984	215489075	atpE;F0 sector of membrane-bound ATP synthase, subunit c	0.00	3.90	0.33
3985	215489076	atpB;F0 sector of membrane-bound ATP synthase, subunit a	0.00	4.86	0.15
3986	215489077	atpI;ATP synthase, membrane-bound accesory subunit	0.00	10.36	0.91
3989	215489080	mioC;FMN-binding protein MioC	0.00	6.78	0.75
3997	215489088	rbsC;D-ribose transporter	0.00	2.25	0.61
3998	215489089	rbsB;D-ribose transporter	0.00	4.74	0.08
3999	215489090	rbsK;ribokinase	0.00	7.84	0.48
4000	215489091	rbsR;DNA-binding transcriptional repressor of ribose metabolism	0.00	4.00	0.14
4004	215489095	yifE;hypothetical protein	0.00	7.20	0.75
4005	215489096	yifB;predicted bifunctional enzyme and transcriptional	0.00	-2.11	0.02
4007	215489098	ilvG;acetolactate synthase II, large subunit IlvG	0.00	-2.03	0.42
4008	215489099	ilvM;acetolactate synthase II, small subunit IlvM	0.00	-2.91	0.67
4010	215489101	ilvD;dihydroxyacid dehydratase	0.00	-4.03	0.70
4011	215489102	ilvA;threonine deaminase	0.00	-2.92	0.58
4014	215489105	ppiC;peptidyl-prolyl cis-trans isomerase C (rotamase C)	0.00	2.54	0.78
4017	215489108	rhlB;ATP-dependent RNA	0.01	2.02	0.09
4018	215489109	trxA;thioredoxin 1	0.00	3.30	0.43
4020	215489111	rho;transcription termination	0.00	2.13	0.22
4022	215489113	rfe;UDP-GlcNAc: undecaprenylphosphate GlcNAc-1-phosphate transferase	0.00	3.05	0.27
4023	215489114	wzzE;entobacterial common antigen (ECA) polysaccharide chain length modulation protein	0.01	2.07	0.12
4024	215489115	rffE;UDP-N-acetyl glucosamine-2-epimerase	0.00	2.52	0.08
4025	215489116	rffD;UDP-N-acetyl-D-mannosaminuronic acid dehydrogenase	0.00	-2.76	0.46
4032	215489123	wzyE;predicted WzyE protein involved in ECA polysaccharide chain elongation	0.00	-3.56	0.27
4035	215489126	aslB;predicted regulator of arylsulfatase activity	0.00	3.80	0.35
4038	215489129	hemX;predicted uroporphyrinogen III methylase	0.00	5.54	0.72
4039	215489130	hemD;uroporphyrinogen III	0.00	3.07	0.52
4040	215489131	hemC;hydroxymethylbilane synthase HemC	0.00	3.04	0.27
4042	215489133	cyaY;frataxin, iron-binding and oxidizing protein	0.01	2.13	0.15
4043	215489134	-;hypothetical protein	0.00	-2.79	0.71
4045	215489136	yifL;predicted lipoprotein	0.00	2.48	0.85
4046	215489137	dapF;diaminopimelate	0.00	3.02	0.73
4049	215489140	yigB;predicted hydrolase	0.00	2.67	0.07
4051	215489142	-;hypothetical protein	0.00	6.38	0.16
4057	215489148	yigI;hypothetical protein	0.00	5.75	0.73
4058	215489149	pldA;outer membrane	0.00	8.14	0.31

4070	215489161	–;transketolase	0.00	–2.47	0.51
4078	215489170	–;cysteine hydrolases superfamily protein	0.00	–3.31	0.47
4082	215489174	ubiE;bifunctional 2–octaprenyl–6–methoxy–1,4–benzoquinone methylase/S–adenosylmethionine: 2–DMK	0.00	2.33	0.11
4085	215489177	tatA;TatABCE protein translocation system subunit	0.00	4.49	0.27
4086	215489178	tatB;TatABCE protein translocation system subunit	0.00	2.87	0.55
4089	215489181	rfaH;DNA–binding transcriptional antiterminator RfaH	0.00	3.95	0.56
4093	215489185	fadB;fused 3–hydroxybutyryl–CoAepimerase/delta(3)–cis–delta(2)–trans–enoyl–CoAisomerase/enoyl–CoA hydratase/3–hydroxyacyl–	0.00	2.36	0.42
4094	215489186	pepQ;proline dipeptidase	0.00	2.31	0.25
4096	215489188	trkH;potassium transporter	0.01	–2.14	0.16
4106	215489198	yihA;GTP–binding protein	0.00	10.67	0.08
4111	215489203	glnA;glutamine synthetase	0.00	–2.31	0.79
4115	215489207	–;predicted oxidoreductase with NAD(P)–binding Rossmann–fold domain	0.00	–3.84	0.58
4117	215489209	–;predicted dehydrogenase	0.00	–6.89	0.66
4125	215489217	–;predicted lipase	0.00	5.33	0.58
4126	215489218	–;hypothetical protein	0.00	2.39	0.44
4127	215489219	–;hypothetical protein	0.00	3.24	0.42
4131	215489223	fdoH;formate dehydrogenase–O, Fe–S subunit FdoH	0.00	–2.43	0.35
4132	215489224	fdoG;formate dehydrogenase–O, large subunit	0.00	–2.36	0.35
4133	215489225	fdhD;formate dehydrogenase formation protein	0.00	3.50	0.45
4134	215489226	–;predicted heat shock protein (Hsp90–family)	0.00	–2.59	0.16
4136	215489228	frvR;predicted regulator	0.00	–2.33	0.22
4148	215489240	sodA;superoxide dismutase, Mn	0.01	2.20	0.51
4153	215489245	cpxP;periplasmic protein combats stress	0.00	2.59	0.77
4154	215489246	fieF;zinc transporter	0.00	2.72	0.24
4160	215489252	yiiR;conserved inner membrane	0.00	2.96	0.60
4161	215489253	yiiS;hypothetical protein	0.00	–2.63	0.43
4163	215489255	fpr;ferredoxin–NADP reductase	0.00	2.98	0.42
4167	215489259	yiiU;hypothetical protein	0.00	2.81	0.60
4170	215489262	hslU;molecular chaperone and ATPase component of HslUV	0.00	–2.35	0.06
4173	215489265	cytR;DNA–binding transcriptional dual regulator	0.00	2.43	0.97
4179	215489271	metL;fused aspartokinase II/homoserine dehydrogenase II	0.00	2.89	0.30
4183	215489275	–;hypothetical protein	0.00	3.26	0.54
4186	215489278	katG;catalase/hydroperoxidase	0.01	–2.29	0.43
4187	215489279	yijE;predicted permease	0.00	2.50	0.29
4188	215489280	yijF;hypothetical protein	0.00	–2.43	0.47
4193	215489286	frwC;predicted enzyme IIC component of PTS	0.00	–3.16	0.82
4196	215489289	pflC;pyruvate formate lyase II	0.00	–3.57	0.44
4199	215489292	yijP;conserved inner membrane	0.01	2.28	0.40
4204	215489297	argH;argininosuccinate lyase	0.00	–4.89	0.67
4206	215489299	–;predicted transporter	0.00	–2.92	0.43
4209	215489302	–;predicted amino acid	0.00	–2.09	0.48
4211	215489304	fabR;DNA–binding transcriptional repressor	0.00	3.82	0.17
4212	215489305	yijD;conserved predicted inner membrane protein	0.00	3.65	0.34
4215	215489308	murI;glutamate racemase MurI	0.00	2.63	0.19

4217	215489310	birA;bifunctional protein BirA biotin-[acetylCoA carboxylase] holoenzyme synthetase and DNA-binding transcriptional repressor, bio-5'-AMP-binding	0.00	2.17	0.10
4218	215489311	coaA;pantothenate kinase	0.00	11.20	0.53
4220	215489313	secE;preprotein translocase membrane subunit	0.01	2.17	0.18
4221	215489314	nusG;transcription termination	0.00	3.78	0.67
4222	215489315	rplK;50S ribosomal subunit	0.00	3.47	0.24
4223	215489316	rplA;50S ribosomal subunit	0.00	2.48	0.36
4225	215489318	rplL;50S ribosomal subunit protein L7/L12	0.01	2.11	0.28
4226	215489319	rpoB;RNA polymerase, beta	0.00	-4.36	0.28
4227	215489320	rpoC;RNA polymerase, beta prime subunit	0.00	-2.39	0.45
4230	215489323	thiS;sulphur carrier protein	0.01	-2.16	0.54
4238	215489331	yjaG;hypothetical protein	0.00	2.78	0.09
4240	215489333	yjaH;hypothetical protein	0.01	2.08	0.24
4241	215489334	zraP;Zn-binding periplasmic	0.00	8.92	0.67
4244	215489337	purD;phosphoribosylglycinamide synthetase phosphoribosylamine-glycine	0.00	-2.40	0.48
4249	215489342	aceB;malate synthase A	0.00	-7.03	0.49
4250	215489343	aceA;isocitrate lyase	0.00	-15.31	0.76
4251	215489344	aceK;isocitrate dehydrogenase kinase/phosphatase	0.00	-4.57	0.24
4254	215489347	-;hypothetical protein	0.00	3.62	0.25
4256	215489349	yjbB;predicted transporter	0.00	-3.66	0.26
4259	215489352	pepE;(alpha)-aspartyl	0.00	2.93	0.60
4262	215489355	-;sorbose-permease PTS system IIC component	0.00	-2.38	0.14
4264	215489357	-;sorbose-permease PTS system IIA component	0.01	-2.04	0.29
4265	215489358	-;predicted sorbitol-6-phosphate 2-dehydrogenase	0.00	-2.30	0.24
4267	215489360	rluF;23S rRNA pseudouridine	0.00	4.60	0.14
4268	215489361	pagB;hypothetical protein	0.00	2.46	0.57
4270	215489363	pgi;glucosephosphate isomerase	0.00	-2.71	0.37
4273	215489366	yjbG;hypothetical protein	0.01	-2.00	0.48
4275	215489368	yjbT;hypothetical protein	0.01	-2.12	0.58
4279	215489372	malE;maltose transporter subunit	0.00	3.61	1.32
4282	215489375	-;transposase of ISEc13	0.01	2.03	0.65
4284	215489377	ubiC;chorismate pyruvate lyase	0.00	3.08	0.54
4288	215489381	lexA;DNA-binding transcriptional repressor of SOS regulon	0.00	2.60	0.62
4291	215489384	zur;DNA-binding transcriptional repressor, Zn(II)-binding	0.00	3.81	0.69
4296	215489389	-;predicted nicotinamide mononucleotide transporter	0.00	-2.49	0.56
4308	215489401	aphA;acid phosphatase/phosphotransferase, class B, non-specific	0.00	4.44	0.68
4312	215489405	ssb;single-stranded DNA-binding protein	0.00	3.44	0.18
4314	215489407	yjcC;predicted signal transduction protein (EAL domain containing protein)	0.01	-2.07	0.37
4316	215489409	soxR;DNA-binding transcriptional dual regulator, Fe-Scenter for redox-sensing	0.00	7.30	0.59
4319	215489412	actP;acetate transporter	0.02	-2.07	0.32
4321	215489414	acs;acetyl-CoA synthetase	0.02	-2.03	0.67
4330	215489423	yjcO;hypothetical protein	0.00	5.11	0.20
4331	215489424	-;predicted ABC transporter ATP-binding protein	0.00	-2.23	0.23
4332	215489425	-;predicted ABC transporter, ATP-binding protein	0.00	-2.51	1.00
4333	215489426	-;predicted ABC transporter, permease protein	0.00	-2.13	0.38

4335	215489428	–;predicted ABC–type dipeptide transport system, periplasmic component	0.00	–2.35	0.29
4336	215489429	fdhF;formate dehydrogenase–H, selenopolypeptide subunit	0.02	–2.26	1.12
4344	215489437	alsC;D–allose transporter subunit	0.00	–2.76	0.23
4345	215489438	alsA;fused D–allose transporter subunits of ABC superfamily: ATP binding components	0.00	–3.24	0.39
4349	215489442	yjdP;hypothetical protein	0.00	–2.67	0.27
4351	215489444	phnO;predicted acyltransferase with acyl–CoA N–acyltransferase	0.00	3.76	0.42
4357	215489450	phnI;carbon–phosphorus lyase complex subunit PhnI	0.01	–2.10	1.25
4361	215489454	phnE;phosphonate/organophosphate ester transporter subunit PhnE, membrane component of ABC superfamily	0.01	–2.16	0.38
4364	215489457	yjdN;hypothetical protein	0.00	2.55	0.64
4365	215489458	yjdM;hypothetical protein	0.00	–2.42	0.21
4371	215489464	eptA;predicted metal dependent hydrolase EptA	0.00	3.63	0.48
4383	215489476	yjdJ;predicted acyltransferase with acyl–CoAN–acyltransferase	0.00	–2.33	1.15
4386	215489479	yjdL;predicted transporter	0.00	–2.80	0.55
4389	215489482	yjdC;predicted transcriptional	0.00	2.99	0.54
4393	215489486	aspA;aspartate ammonia–lyase	0.01	2.17	0.87
4398	215489491	yjeI;hypothetical protein	0.00	6.42	0.80
4400	215489493	yjeK;predicted lysine	0.00	4.33	0.59
4401	215489494	efp;Elongation factor EF–P	0.01	2.26	0.16
4402	215489495	ecnA;entericidin A membrane lipoprotein, antidote to	0.00	3.49	0.59
4403	215489496	ecnB;entericidin B membrane lipoprotein	0.00	–2.60	0.90
4404	215489497	sugE;multidrug efflux system	0.00	3.06	0.18
4405	215489498	blc;outer membrane lipoprotein (lipocalin) Blc	0.00	2.38	0.82
4407	215489500	frdD;fumarate reductase (anaerobic), membrane anchor	0.00	3.19	0.60
4413	215489506	yjeP;predicted mechanosensitive channel	0.00	–3.46	0.22
4415	215489508	rsgA;ribosome small subunit–dependent GTPase A	0.00	2.63	0.24
4416	215489509	orn;oligoribonuclease	0.00	5.59	0.44
4420	215489513	amiB;N–acetylmuramoyl–L–alanine amidase II	0.00	2.71	0.63
4425	215489518	hflK;modulator for HflB protease specific for phage lambda cII	0.00	–3.13	0.90
4427	215489520	yjeT;conserved inner membrane	0.00	–2.82	0.30
4433	215489526	yjfJ;predicted transcriptional regulator effectorprotein	0.01	–2.00	0.10
4434	215489527	yjfK;hypothetical protein	0.00	–2.64	0.61
4438	215489531	aidB;isovaleryl CoA	0.00	–10.95	0.87
4439	215489532	yjfN;hypothetical protein	0.00	2.86	0.49
4444	215489537	ulaA;L–ascorbate–specific enzyme IIC component of PTS	0.00	–6.45	0.53
4445	215489538	ulaB;L–ascorbate–specific enzyme IIB component of PTS	0.00	–3.78	0.43
4446	215489539	ulaC;L–ascorbate–specific enzyme IIA component of PTS	0.00	–4.68	0.29
4450	215489543	yjfY;hypothetical protein	0.00	2.48	0.52
4451	215489544	rpsF;30S ribosomal subunit	0.01	2.02	0.22
4452	215489545	priB;primosomal protein N	0.01	2.08	0.99
4459	215489552	–;predicted 3–hydroxybutyryl–CoA dehydratase	0.00	–2.22	0.86
4463	215489556	ytfB;predicted cell envelope opacity–associated protein	0.00	4.86	0.16
4470	215489563	cpdB;2': 3'–cyclic–nucleotide 2'–phosphodiesterase	0.00	2.74	0.53
4472	215489565	ytfJ;predicted transcriptional	0.00	2.83	0.09
4474	215489567	ytfL;predicted inner membrane	0.00	3.78	0.22

4478	215489571	ytfP;hypothetical protein	0.00	4.08	0.33
4479	215489572	ppa;inorganic pyrophosphatase	0.00	5.54	0.30
4480	215489573	ytfQ;predicted sugar transporter subunit: periplasmic-binding component of ABC superfamily	0.00	7.20	0.57
4482	215489575	ytfT;predicted sugar transporter subunit: membrane component of ABC superfamily	0.00	-2.79	0.48
4483	215489576	yjfF;predicted sugar transporter subunit: membrane component of ABC superfamily	0.01	-2.04	0.57
4485	215489578	mpl;UDP-N-acetylmuramate: L-alanyl-gamma-D-glutamyl-meso-diaminopimelate ligase	0.00	2.75	0.19
4490	215489583	nrdD;anaerobic ribonucleoside-triphosphate reductase	0.00	-2.41	0.15
4495	215489588	yjgF;ketoacid-binding protein	0.00	2.73	0.29
4499	215489592	yjgH;predicted mRNA endoribonuclease	0.00	2.35	0.25
4500	215489593	yjgI;predicted oxidoreductase with NAD(P)-binding Rossmann-fold domain	0.01	-2.15	0.13
4503	215489596	argI;ornithine	0.01	-2.02	0.19
4507	215489600	valS;valyl-tRNA synthetase	0.02	-2.02	0.72
4509	215489602	pepA;aminopeptidase A, a cyteinylglycinase	0.01	-2.14	0.14
4512	215489605	yjgR;predicted ATPase	0.00	3.31	0.48
4515	215489608	-;hypothetical protein	0.00	3.32	0.09
4522	215489615	-;hypothetical protein	0.00	-3.46	0.63
4526	215489619	-;predicted capsid protein	0.00	-2.77	0.26
4527	215489620	-;predicted endonuclease, HNH	0.00	-3.65	0.50
4531	215489624	-;hypothetical protein	0.00	4.10	0.16
4534	215489627	-;predicted type I restriction-modification enzyme S subunit	0.00	-2.34	0.16
4537	215489630	-;McrB family protein, predicted GTPase subunit of restriction endonuclease	0.00	-2.84	0.62
4538	215489631	-;McrC family protein, predicted McrBC 5-methylcytosine restriction system component	0.00	-2.52	0.35
4539	215489632	-;predicted restriction	0.00	3.53	0.26
4541	215489634	yjhT;hypothetical protein	0.00	2.73	0.50
4545	215489638	fimA;major type 1 subunit fimbrin	0.00	-3.21	0.85
4548	215489641	fimD;outer membrane usher protein, type 1 fimbrial synthesis	0.00	-2.75	0.46
4551	215489644	fimH;minor component of type 1	0.00	2.81	0.23
4552	215489645	uxuA;mannonate hydrolase	0.00	2.70	0.66
4576	215489669	-;predicted Na+/H+ and K+/H+ transporter	0.00	-3.81	0.20
4580	215489673	yjjA;hypothetical protein	0.01	2.12	0.21
4582	215489675	dnaT;DNA biosynthesis protein (primosomal protein I)	0.01	2.03	0.25
4584	215489677	yjjP;predicted inner membrane	0.00	3.77	0.45
4585	215489678	yjjQ;predicted DNA-binding transcriptional regulator	0.01	-2.95	0.98
4586	215489679	bglJ;DNA-binding transcriptional activator	0.01	-2.04	0.77
4587	215489680	fhuF;ferric iron reductase involved in ferrichydroximate	0.01	2.07	0.27
4589	215489682	rsmC;16S rRNA m2G1207	0.00	2.73	0.08
4591	215489684	rimI;acetylase for 30S ribosomal subunit protein S18	0.00	5.61	0.35
4597	215489690	yjjV;predicted DNase	0.00	-2.42	0.25
4613	215489706	slt;lytic murein transglycosylase, soluble	0.00	-2.66	0.48
4617	215489710	creA;hypothetical protein	0.00	2.24	0.09
4619	215489712	creC;sensory histidine kinase CreC in two-component regulatory system with CreB or PhoB, regulator of the CreBC	0.00	-2.61	0.61
