# Supplementary material for: Mutating both relA and spoT of enteropathogenic Escherichia coli E2348/69 attenuates its virulence and induces interleukin 6 in vivo
Source: Front Microbiol. 2023 Mar 2;14:1121715. doi: 10.3389/fmicb.2023.1121715 (PMC10017862; doi:10.3389/fmicb.2023.1121715)
Supplement: Supplementary file 7 [file Table_4.DOCX]

**Supplementary Table 4. Functional categorization of differentially expressed genes in 3D4/31 infected with a Δ*relA*Δ*spoT* EPEC**

| Functional category | Number of genes | Gene names |
| --- | --- | --- |
| Up-regulated genes | 77 |  |
| Transcription factor | 6 | *CEBPB, CEBPD, TCIM, JUNB, NFKB1, NFKB2* |
| Cytokine | 5 | *IL-6, IL-8, GM-CSF, MCP-1, MIP2-A* |
| Stress-inducible protein | 3 | *GADD45A, TIPARP, , TXNIP* |
| Plasminogen activator | 3 | *PLAU, PLAUR, PLAT* |
| Zinc binding protein | 1 | *MT-2B* |
| Epidermal growth factor | 1 | *AREG* |
| Metabolic enzyme | 1 | *SAT1* |
| Others | 55 | *ICAM1, F3, SDC4, CYR61, etc.* |
| Down-regulated genes | 5 |  |
| Transcription factor | 1 | *ID1* |
| Heat shock protein | 1 | *HSPA8* |
| Others | 3 | *RRM2, SRSF6, MAT2A* |
