# Supplementary material for: Mutating both relA and spoT of enteropathogenic Escherichia coli E2348/69 attenuates its virulence and induces interleukin 6 in vivo
Source: Front Microbiol. 2023 Mar 2;14:1121715. doi: 10.3389/fmicb.2023.1121715 (PMC10017862; doi:10.3389/fmicb.2023.1121715)
Supplement: Supplementary file 8 [file Table_5.DOCX]

**Supplementary Table 5. Validation of transcriptional patterns in 3D4/31 infected with a Δ*relA*Δ*spoT* EPEC.**

| Gene | RNA-seq | qRT-PCR | |
| --- | --- | --- | --- |
|  | Fold change (Δ*relA*Δ*spoT* /WT) | Fold change^a^ (Δ*relA*Δ*spoT* /WT) | *p*-value |
| *HSPA8* | -4.0 | 0.5±0.0 | < 0.001 |
| *PLAU* | 4.3 | 5.7±0.8 | 0.009 |
| *AREG* | 2.2 | 4.9±0.9 | 0.010 |
| *GM-CSF* | 3.7 | 5.0±0.9 | 0.006 |
| *F3* | 7.5 | 8.4±0.9 | 0.010 |
| *MCP-1* | 2.5 | 5.6±0.8 | 0.004 |
| *MT-2B* | 8.3 | 11.2±3.1 | 0.019 |
| *CEBPB* | 2.2 | 6.9±0.7 | 0.010 |
| *SDC4* | 3.3 | 2.2±0.5 | 0.043 |
| *MIP2-A* | 2.6 | 4.3±0.2 | 0.008 |
| *ICAM1* | 2.9 | 2.1±0.9 | 0.320 |

^a^ All data are shown as mean ± standard error from three independent experiments.
