# Supplementary material for: Mutating both relA and spoT of enteropathogenic Escherichia coli E2348/69 attenuates its virulence and induces interleukin 6 in vivo
Source: Front Microbiol. 2023 Mar 2;14:1121715. doi: 10.3389/fmicb.2023.1121715 (PMC10017862; doi:10.3389/fmicb.2023.1121715)
Supplement: Supplementary file 9 [file Table_6.docx]

**Supplementary Table 6. Differentially expressed cytokine genes in 3D4/31 infected with the Δ*relA*Δ*spoT* EPEC.**

| Gene | Product | Fold change^a^ (Δ*relA*Δ*spoT* /WT) | Function |
| --- | --- | --- | --- |
| *IL-8* | Interleukin 8 | 5.4 | Angiogenesis, chemotaxis of neutrophil |
| *GM-CSF* | Granulocyte-macrophage colony-stimulating factor | 3.7 | Proliferation of granulocytes and macrophages |
| *IL-6* | Interleukin 6 | 2.8 | Pleotropic effects on both innate and adaptive immunity |
| *MIP2-A* | Macrophage inflammatory protein 2 alpha | 2.6 | Angiogenesis, chemotaxis of neutrophil |
| *MCP-1* | Monocyte chemoattractant protein 1 | 2.5 | Chemotaxis of monocytes and macrophages |

^a^The datasets presented in this study can be found in the NCBI Sequence Read Archive (<https://www.ncbi.nlm.nih.gov/bioproject>) under accession number PRJNA917101. Raw reads of the RNA-seq from two samples were deposited in the NCBI SRA (<http://www.ncbi.nlm.nih.gov/Traces/sra>) under accession numbers SRR23329177 and SRR23329178.
